# *Cis*–*Trans* Isomerism and Hydride Thermodynamics Govern H_2_ Activation and Hydrogenation Activity in Indium(III) Pincer Complexes

**DOI:** 10.1021/acs.inorgchem.6c02395

**Published:** 2026-07-15

**Authors:** Pritha Saha, Gabriela Gastelu, Leandro D. Mena, Róbert Gyepes, Jorge G. Uranga, Martin Hulla

**Affiliations:** † Faculty of Science, Department of Inorganic Chemistry, 112302 Charles University, Albertov 6, Praha 2 128 00, Czech Republic; ‡ Departamento de Química Orgánica, Facultad de Ciencias Químicas, Universidad Nacional de Córdoba, Córdoba X5000HUA, Argentina; § Instituto de Investigaciones En Físico-Química Córdoba, Universidad Nacional de Córdoba (INFIQC−CONICET), Córdoba 5000, Argentina; ∥ Department of Chemistry, Faculty of Education, J. Selye University, Bratislavská 3322, Komárno 945 01, Slovakia

## Abstract

Trivalent boron Lewis acids activate H_2_ for catalytic hydrogenations, yet only one trivalent indium analog is known to do so. Nevertheless, heavier main-group elements also form five-, six-, and seven-coordinate compounds. However, complexes of Ga and In generally fail at hydrogenation catalysis, without clear guiding principles for their design. We show that coordination geometry, hydride thermodynamics, and the counteranion govern catalytic imine hydrogenation at In­(III) pincer complexes. Cationic five-coordinate In complexes bearing NNN-, PNN-, and PNP-pincer ligands with [InX_4_]^−^ anions catalyze this reaction under 120 °C and 15 bar of H_2_. Catalytic activity correlated with the Gibbs free energy of H_2_ activation. This endergonic step determined turnover. Rather than classical Lewis acidity descriptors, hydride and fluoride affinity, and Gutmann–Beckett acidity, *cis*–*trans* isomerism of key intermediates controlled H_2_ activation thermodynamics. Indium complexes outperformed lighter group-13 Al and Ga congeners not for their higher Lewis acidity, but for their more favorable hydride formation thermodynamics. The ligands modulated Lewis base binding without overcoming hydride thermodynamic limitations. Finally, the [InX_4_]^−^ anions may activate the imine substrate toward reduction. Our findings explain the hydrogenation activity of indium Lewis acids, establishing coordination geometry, hydride thermodynamics, and counteranion as key parameters for their rational design.

## Graphical abstract

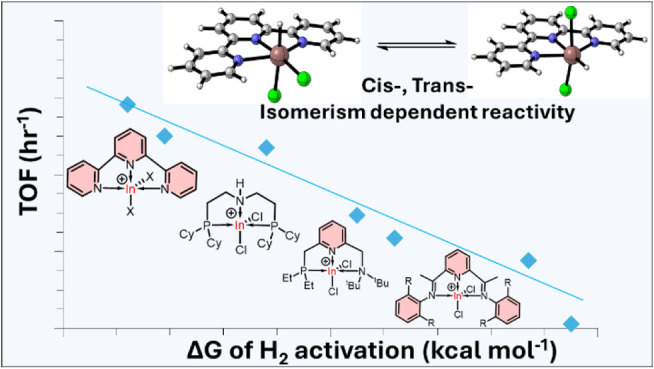

## Introduction

Catalytic hydrogenation of unsaturated functional groups, such as C=N, C=O, and C=C, yields fine chemicals, agrochemicals, and pharmaceutically active compounds. These reactions are usually catalyzed by transition metals, but trisubstituted Lewis acidic boranes and their frustrated Lewis pairs (FLPs) enable metal-free heterolytic cleavage of H_2_ and hydrogenation catalysis, leading to novel hydrogenation reactivity.^1–3^ Other main-group tri- and tetra-substituted Lewis acids (LAs) can also activate H_2_ ^4^ and mediate hydrogenations.^5–10^ In addition to tri- and tetra-substituted Lewis acids, heavier congeners, including group 13 Al, Ga, and In, support five-, six-, and even seven-coordinate environments^11,12^ with a high potential for LA tuning and control in Lewis acidic catalysis. Furthermore, gallium and indium offer in some cases, such as ring-opening polymerizations or the activation of unsaturated C–C bonds, superior reactivity, better water tolerance, or unique selectivity not accessible with the more studied aluminium congeners.^13–21^ For now, though, catalytically active coordination compounds of the heavier p-block elements Ga and In remain elusive in hydrogenation catalysis^22–24^ despite their common use in transfer hydrogenation reactions using alcohols or 1,4-cyclohexadiene as the hydride source instead of H_2_ or the use of heterogeneous In_2_O_3_ in high temperature and pressure CO_2_ hydrogenations (250–350 °C, 50–100 bar).^25–29^

Imine hydrogenation serves as the benchmark for Lewis acid- and FLP-catalyzed hydrogenations, with catalysts reported across a wide range of p-block elements.^1^ Such catalysts include heavier congeners of the archetypical FLP Lewis acid B­(C_6_F_5_)_3_ in the form E­(C_6_F_5_)_3_, where E = Al, Ga, and In,^10^ as well as a few three- and four-coordinate aluminum hydrides, such as di-isobutylaluminum hydride (DIBAL-H),^30^ LiAlH_4_,^31–33^ and [(^Dipp^BIAN)­Al­(μ-H)_2_Li­(OEt_2_)_2_].^34^ Three-coordinate cationic aluminum analogs of boreniums (R_2_BL^+^, where L = neutral electron pair donor)^35^ in the form (R_2_AlL^+^)^36^ also catalyze imine hydrogenation. However, only two five-coordinate group 13 LAs have shown any activity in LA-mediated hydrogenations so far, and even then only under harsh conditions (>180 °C and >50 bar H_2_) that caused catalyst, substrate, and product degradation.^22,23^ Further efforts have been limited by 1) insufficient H_2_ activation, 2) LA instability, or 3) LA-hydride inertness.^7,22,23,37^ Unlike well-studied boron-based systems, the fundamental reasons for the inactivity of five- and six-coordinate group 13 LAs remain unknown. Developing these hydrogenation catalysts requires acquiring systematic data on five- and six-coordinate group-13 LA trends and hydrogenation activity under moderate reaction conditions.

The present study unveils trends of cationic five-coordinate group-13 LAs [E­(terpy)­Cl_2_]­[ECl_4_] (E = Al, Ga, In) and [In­(terpy)­X_2_]­[InX_4_] (X = Cl^–^, Br^–^, I^–^) and extends this platform to a series of indium complexes [In­(L^3^)­Cl_2_]­[InCl_4_] with diverse neutral tridentate pincer-type ligands (L^3^ = terpy, ^Dipp^NNN-, ^Ph^NNN, ^tBu^PN^Et^N-, and ^Cy^PNP-pincer) (Figure 1). When testing these complexes as imine hydrogenation catalysts under 15 bar H_2_ and 120 °C, we found that experimental catalytic activity does not correlate with conventional Lewis acidity metrics, such as the Gutmann–Beckett acceptor number (AN)^38,39^ or hydride (HIA)^40^ and fluoride (FIA)^41^ ion affinity but instead tracks with the Gibb’s free energy of H_2_ activation and hydride formation. Furthermore, we investigated the Lewis acidic sites of the metal complex and explored how the halide (X^–^ = Cl^–^, Br^–^, I^–^) and pincer ligands influence these sites, available molecular orbitals, hydride formation thermodynamics, and the *cis-* and *trans-*configurations of the intermediate LA-LB adducts and resulting hydrides as well as the effect of the [InX_4_]^−^ anion by comparison with [B­(C_6_F_5_)_4_]^−^ and [CHB_11_Cl_11_]^−^. Together, these insights enable us to rationalize reactivity trends across a broad series of five-coordinate cationic indium complexes and their anions.

**Figure 1:**
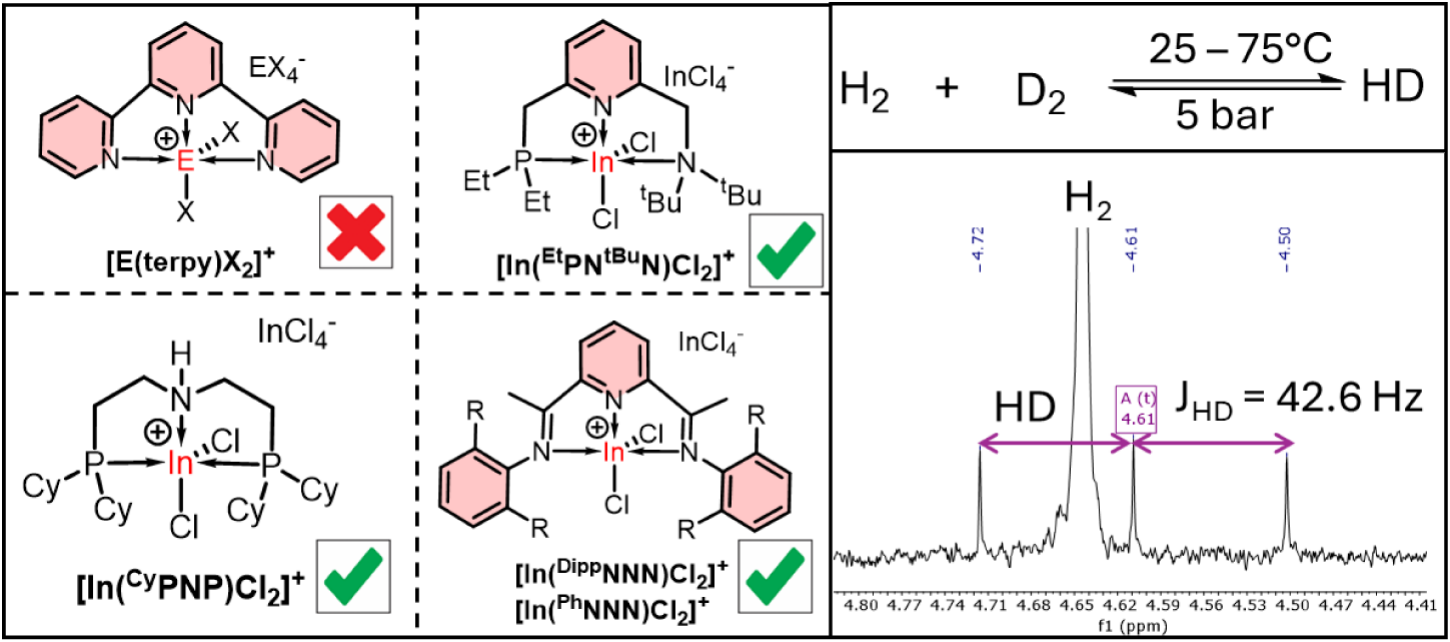
Cationic indium complexes supported by tridentate ligands reversibly activate H_2_, as demonstrated by H/D scrambling, where E = Al, Ga, In and X = Cl^–^, Br^–^, I^–^. H/D exchange experiments were performed with H_2_/D_2_ mixtures (5 bar total pressure) at 25–75 °C. Formation of HD (1:1:1 triplet at 4.61 ppm, *J*_HD_ = 42.6 Hz) confirms reversible dihydrogen activation as observed by ^1^H NMR in CDCl_3_. The complexes include [E­(terpy)­Cl_2_]­[ECl_4_] (E = Al, Ga, In) and [In­(L^3^)­Cl_2_]­[InCl_4_] derivatives with tridentate ligands L^3^.

## Results and Discussion

In combination with various Lewis bases, group 13 complexes [E­(terpy)­Cl_2_]­[ECl_4_] (E = Al, Ga, In) and halides [In­(terpy)­X_2_]­[InX_4_] (X^–^ = Cl^–^, Br^–^, I^–^) failed to activate H_2_, as shown in H/D scrambling experiments, or to form stable hydride complexes (Figure 1). Conversely, [In­(^Dipp^NNN)­Cl_2_]^+^, [In­(^Ph^NNN)­Cl_2_]^+^, [In­(^Cy^PNP)­Cl_2_]^+^, and [In­(^tBu^PN^Et^N)­Cl_2_]^+^ (see SI for synthetic details) led to reversible H_2_ activation between D_2_ and H_2_, yielding the characteristic 1:1:1 triplet of HD gas at 4.61 ppm with *J*_HD_ = 42.6 Hz,^42^ at temperatures ranging from 25 to 75 °C, and at 5 bar of H_2_/D_2_ after several days (Figure 1).

In contrast to the H_2_ activation experiments described above, imine hydrogenation revealed that only the aluminum and gallium complexes remained catalytically inactive (Table 1, entries 1–3). All indium complexes, including [In­(terpy)­X_2_]­[InX_4_], catalyzed imine hydrogenation, reaching 40 to 85% yield of the desired amine product (Table 1) under optimized reaction conditions (120 °C and 15 bar H_2_; see SI for further details) in the presence of pyrrolidine as the reaction base, which reversibly formed an adduct with [In­(terpy)­X_2_]­[InX_4_] of comparable stabilization energy (*vide infra*) as calculated for “encounter complexes” in classical FLPs.^43,44^ The applied conditions were also comparable to those applied for initial boron-based FLPs, which were gradually improved to near-ambient hydrogenations over time.^1,45^ Despite the lack of direct evidence for H_2_ activation, [In­(terpy)­X_2_]­[InX_4_] showed the highest catalytic activity, following the halide trend (I^–^ > Br^–^ > Cl^–^; Table 1, entries 6–8), affording the amine product in 5–22% yield, even at a lower temperature of 100 °C. Using D_2_ instead of H_2_, we confirmed dihydrogen activation in the hydrogenation reaction, which resulted in deuterated amine products. Ultimately, the donor solvent acetonitrile, which is required to solubilize [In­(terpy)­X_2_]­[InX_4_] at low-to-moderate temperatures, was identified as a reaction inhibitor, explaining the absence of H/D scrambling in direct H_2_/D_2_ activation studies.

**Table 1:**
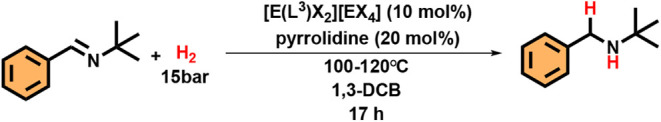
Catalytic Hydrogenation of *N*-*tert*-Butyl-1-phenylmethanimine by Five-Coordinate Indium Lewis Acids in the Presence of Pyrrolidine^a^

Entry	Complex	Temp. (°C)	Yield (%)	TOF (h^–1^)
1	[Al(terpy)Cl_2_]^**+**^	120	NR	0
2	[Ga(terpy)Cl_2_]^**+**^	120	NR	0
3	[Ga(terpy)Br_2_]^**+**^	120	NR	0
4	[In(terpy)Cl_2_]^+^	120	85	0.50
5	[In(terpy)Br_2_]^+^	120	99	≥0.58
6	[In(terpy)Cl_2_]^+^	100	5	0.03
7	[In(terpy)Br_2_]^+^	100	9	0.05
8	[In(terpy)I_2_]^+^	100	22	0.13
9	[In(^Dipp^NNN)Cl_2_]^+^	120	30	0.24
10	[In(^Ph^NNN)Cl_2_]^+^	120	40	0.29
11	[In(^tBu^PN^Et^N)Cl_2_]^+^	120	50	0.29
12	[In(^Cy^PNP)Cl_2_]^+^	120	80	0.47
13	[In(^Cy^PNP)Cl_3_]	120	NR	0

a Reaction conditions: *N*-*tert*-butyl-1-phenylmethanimine (1 mmol), 120 or 100 °C, 15 bar H_2_, 10 mol % LA, 20 mol % LB in 1,3-dichlorobenzene (1,3-DCB), 17 h. All catalytic tests were run in triplicate, and the mean NMR yield was calculated using dibromomethane as the internal standard.

The catalytic activity of the five-coordinate indium complexes in the form [In­(L^3^)­X_2_]­[InX_4_] was not correlated with Lewis acidity descriptors, including the experimental Gutmann–Beckett acidity (AN) and calculated fluoride (FIA) and hydride (HIA) ion affinity (Figure 2) calculated at the DSD-PBEP86/Def2QZVPP//PBEh-3c/mSVP level of theory (SI). This lack of correlation with common Lewis acidity descriptors may stem from inherent methodological limitations, particularly within the Gutmann–Beckett method, such as the poor correlation of the Et_3_PO ^31^P NMR chemical shift with P=O bond weakening, potential binding equilibria (observed by variable temperature ^1^H NMR) including *cis–trans* isomerism, or steric hindrance limiting probe interaction.^39,46^ Furthermore, FIA and HIA values focus on a single favorable interaction,^40^ overlooking the thermodynamic barrier imposed by Lewis base binding prior to H_2_ activation. Instead, hydrogenation activity was inversely correlated with the Gibbs free energy of H_2_ activation by the LA and pyrrolidine. Accordingly, this reaction should be primarily limited by unfavorable H_2_ activation thermodynamics.

**Figure 2:**
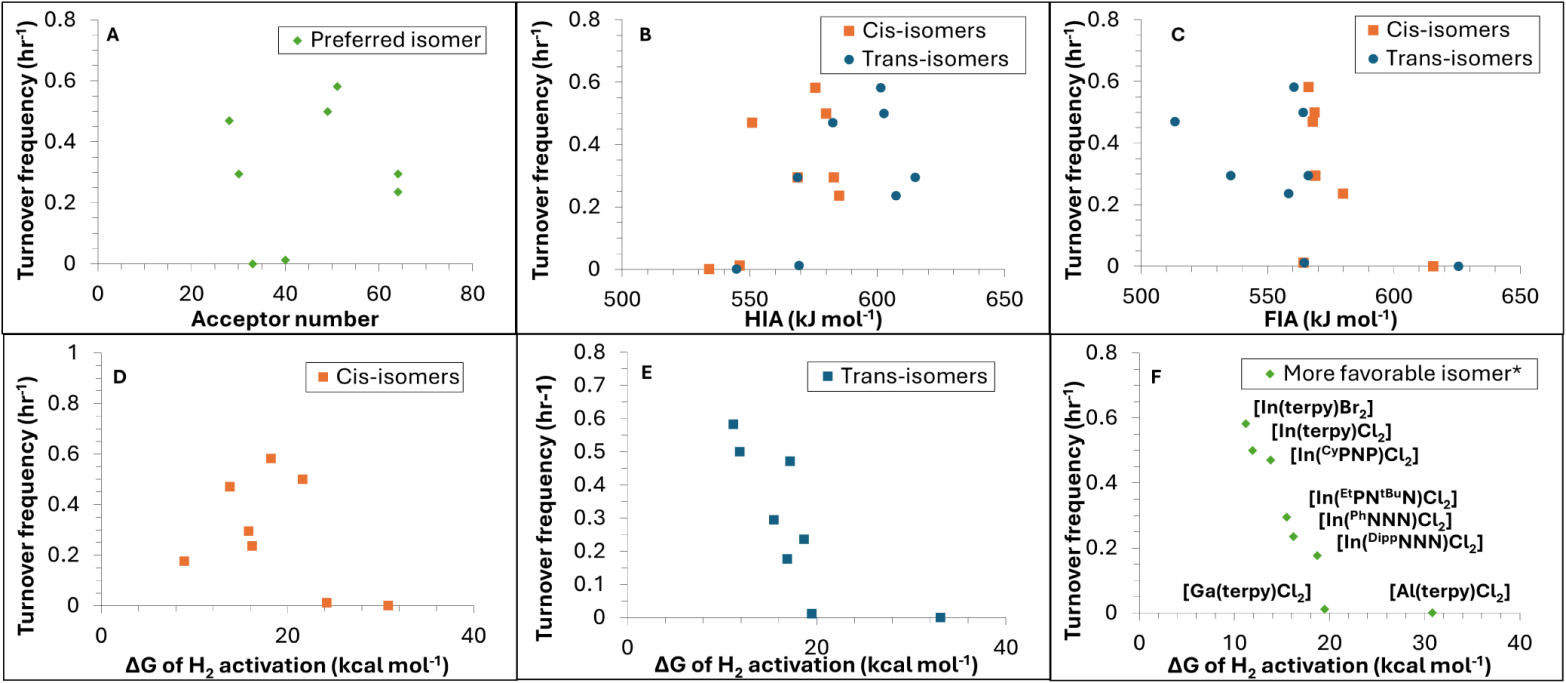
Catalytic activity of cationic five-coordinate group 13 Lewis acids [In­(L^3^)­X_2_]­[InX_4_] correlates with the *Gibbs free energy pathway of dihydrogen activation leading to the thermodynamically more favorable hydride (either *cis-* or *trans-*) rather than with commonly applied acidity descriptors. The plots compare turnover frequency (TOF) with Gutmann–Beckett acceptor number (Plot A), calculated hydride (HIA) and fluoride (FIA) ion affinity values (Plots B and C), and the Gibbs free energy of H_2_ activation (Plots D and E for *cis-* and *trans-*isomers, respectively) with the lowest-energy pathways (either *cis-* or *trans-*isomer, Plot F). Considering that the thermodynamically preferred isomer results in a strong correlation (*R* ^2^ = 0.94) highlights the role of coordination geometry in controlling catalytic activity.

In boron-based hydrogenations, H_2_ activation thermodynamics depend on the combined strength of the LA and LB, the stability of the resulting LA-hydride and protonated base, and their newly formed ionic interactions, with strong LA-LB binding and nonbonding interactions in FLPs suppressing reactivity, as shown computationally.^47,48^ But in the five-coordinate indium catalysts studied here, an additional factor must be considered, that is, *cis–trans* isomerization during LB coordination and hydride generation. Based on the inverse correlation between catalyst turnover frequency (TOF) and the Δ*G* of H_2_ activation, the reaction likely proceeds predominantly via the *trans-*isomer although the *cis-*isomer may be favored in some cases. Taking into account the thermodynamically preferred isomer results in a strong linear correlation (*R* ^2^ = 0.94; Figure 2) when the unreactive Al and Ga congeners are excluded.

Our detailed structural analysis (Figure 3) showed that In-hydride formation is endergonic, even in relation to the free reactants, and becomes more energetically unfavorable after LA-LB adduct formation. Moreover, except for [In­(^Ph/Dipp^NNN)­Cl_2_]^+^, LB binding thermodynamically favors the isomer opposite to that preferred for hydride binding, thus requiring ligand rearrangement before or after hydride formation. Case in point, [E­(terpy)­X_2_]^+^ complexes (E = Al, Ga, In and X = Cl^–^, Br^–^, I^–^) preferentially bind to LB in the *cis-*dihalide configuration, whereas hydride formation is favored in the *trans-*dihalide configuration. As a result, H_2_ activation becomes thermodynamically prohibitive for Al and Ga due to low hydride stability in the *cis*-configuration and highly endergonic LB interactions with LA in the *trans*-configuration.

**Figure 3:**
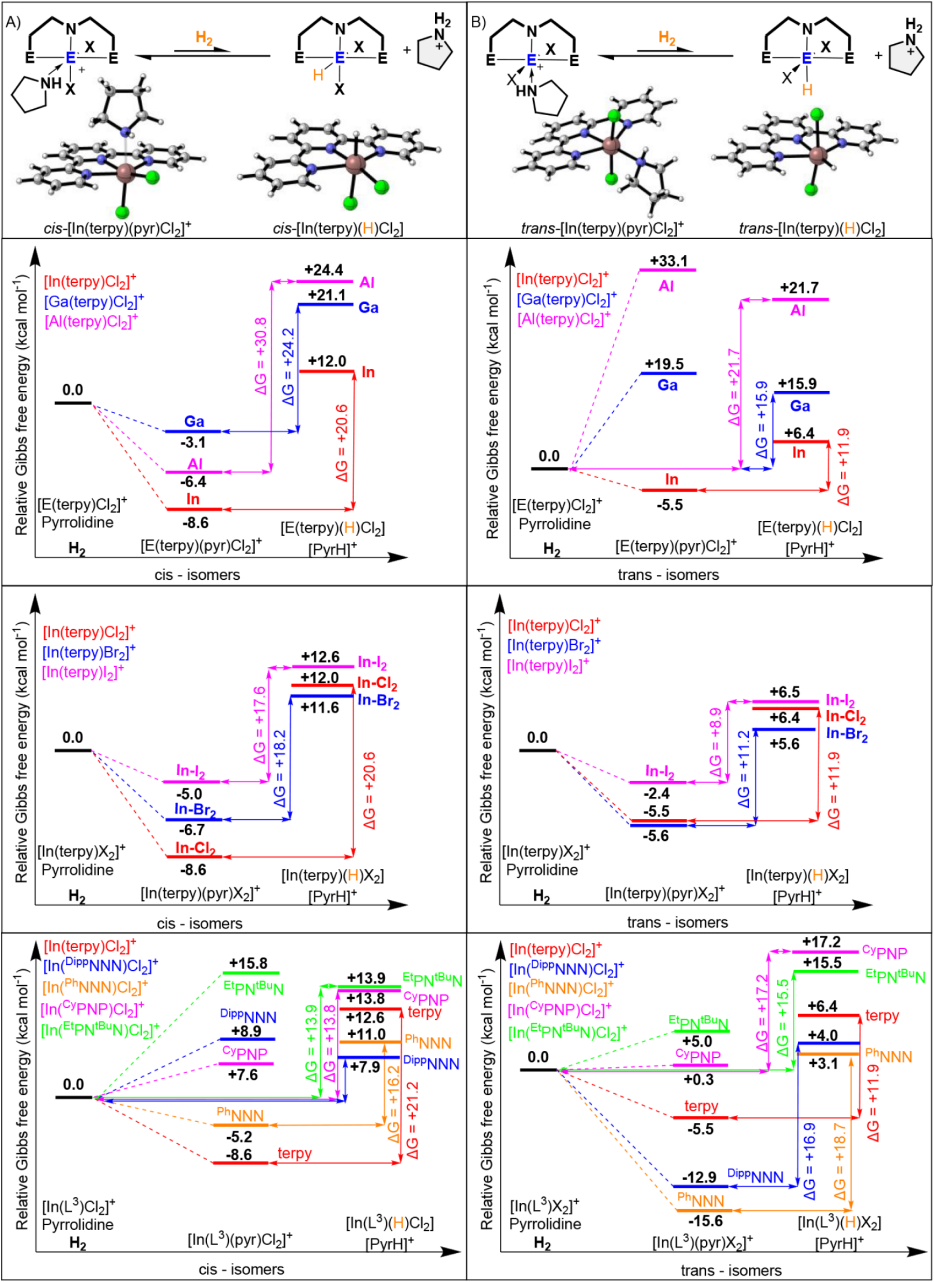
Relative Gibb’s free energy of pyrrolidine binding predominantly favors the opposite isomer to hydride binding. Due to this mismatch, geometric reorganization becomes a necessary step prior to or during H_2_ activation. A) *cis-* and B) *trans-*dihalide isomers. Gibb’s free energy computed at the M06–2X-D3BJ/def2-TZVP level of theory in toluene.

LB interactions with indium complexes ranged from exergonic (−15.6 kcal mol^–1^) to endergonic (+15.8 kcal mol^–1^; Figure 3), varying as a function of both the tridentate ligand and the halide substituents, and only weakly correlating with Gutmann–Beckett AN and even less with HIA or FIA (see SI) demonstrating the limitations of these methods.^39,40,49^ The tridentate ligand modulated the acidity and hydride stability of the complexes, but its dominant effect on LB binding affinity precluded a direct correlation between acidity and hydrogenation performance. Substituting the anionic halide ligands (Cl^–^, Br^–^, I^–^) had little effect on In-hydride stability but destabilized the LA-LB adduct, thereby improving H_2_ activation thermodynamics. Consequently, hydrogenation activity increased following the trend I^–^ > Br^–^ > Cl^–^ indicating that system improvements can be achieved through mild destabilization of the Lewis acid–base adducts and thermodynamic indium-hydride stabilization.

The halide ligands must undergo a considerable rearrangement during LB binding. While all cationic complexes adopt a distorted trigonal bipyramidal configuration (Figure 4), they transition to an octahedral geometry upon ligand binding. Two LA orbitals on the indium center facilitate this coordination, resulting in either *cis-* or *trans-*dihalide isomers (Figure 4). The lowest unoccupied molecular orbital (LUMO), characterized by its primarily *s*-character, directs the ligand approach between the halides, transverse to the tridentate ligand, yielding the *trans-*dihalide geometry. Conversely, the LUMO + *n* orbital (*n* = 1–10), with a predominantly *p*-orbital character, facilitates access over the tridentate ligand, yielding the *cis-*dihalide geometry. But as shown by single-crystal XRD (Figure 4), [In­(^Dipp^NNN)­Cl_2_]^+^ forms a *cis-*dihalide complex with acetonitrile (Figure 4b) even though LB binding in the *trans-*dihalide configuration is thermodynamically preferred (Figure 3). Accordingly, the *cis-*dihalide configuration is the kinetically favored outcome of LB binding. Experiments with tricyclohexylphosphine (PCy_3_) and [In­(terpy)­Br_2_]­[InBr_4_] indeed show rapid formation of a single adduct at 25 °C, yet upon heating, a second adduct is detected by ^31^P NMR together with a broad exchange peak indicative of two complexes and an equilibrium with free PCy_3_. Similar effects are observable at 25 °C and −20 °C with the applied LB pyrrolidine.

**Figure 4:**
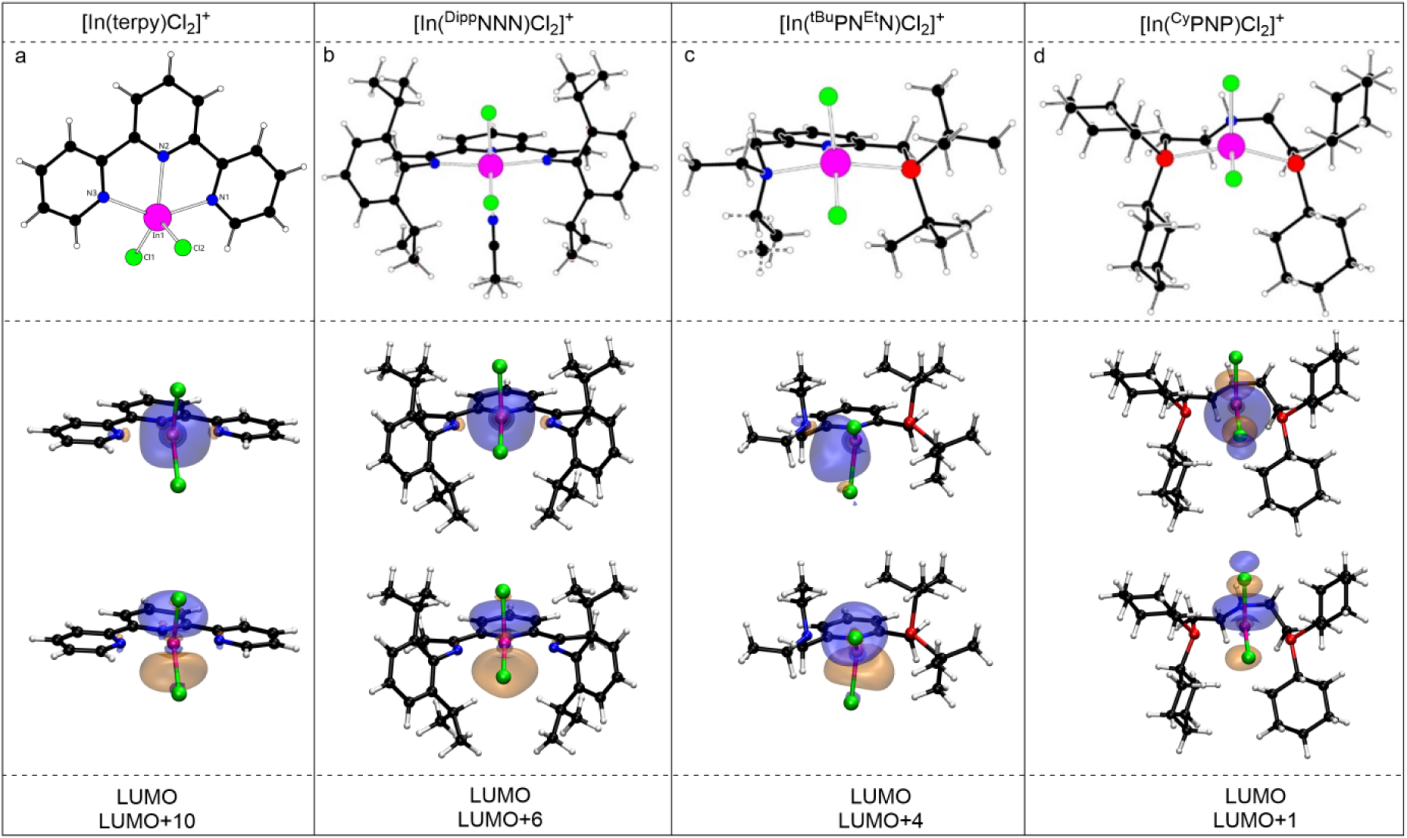
(a-d) Ligands modulate vacant orbital energy and accessibility thereby affecting LA-LB adduct formation, geometry, and stability. Single-crystal XRD (see SI) of representative catalysts is shown alongside their computed indium-centered vacant orbitals (LUMO and LUMO + *n*). Orbital orientation determines the preferred approach of the Lewis base, leading to either *cis*- or *trans*-dihalide configurations and influencing adduct stability and catalytic reactivity.

The exception to this trend is the [In­(^Cy^PNP)­Cl_2_]^+^ complex, where both LUMO and LUMO + 1 are p-type orbitals with lobes between the halides and above the tridentate ligand plane. This electronic structure suggests that the same orbital(s) mediates the formation of both isomers. Nevertheless, the trajectory of ligand approach remains unchanged, whether it is between the halides or over the tridentate ligand. The resulting interaction of the LB with [In­(^Cy^PNP)­Cl_2_]^+^ is endergonic because the cyclohexyl substituents and the flexible linker restrict access over the ligand and the halide substituents sterically hinder access from the open coordination face.

Catalytic turnover requires the vacant sixth coordination site, as evidenced by the inactivity of the neutral [In­(L^3^)­Cl_3_] congeners. Although some derivatives partly undergo “self-ionization”,^22,50^ yielding mildly active mixtures of cationic and neutral species, most of the neutral compounds must be activated by ionization with InCl_3_ or other LAs. For example, the neutral [In­(^Cy^PNP)­Cl_3_] showed no catalytic activity in imine hydrogenations (Table 1, entry 13) but generated the catalytically active five-coordinate [In­(^Cy^PNP)­Cl_2_]^+^ cation (Table 1, entry 12), as confirmed by single-crystal XRD (Figure 5).

**Figure 5:**
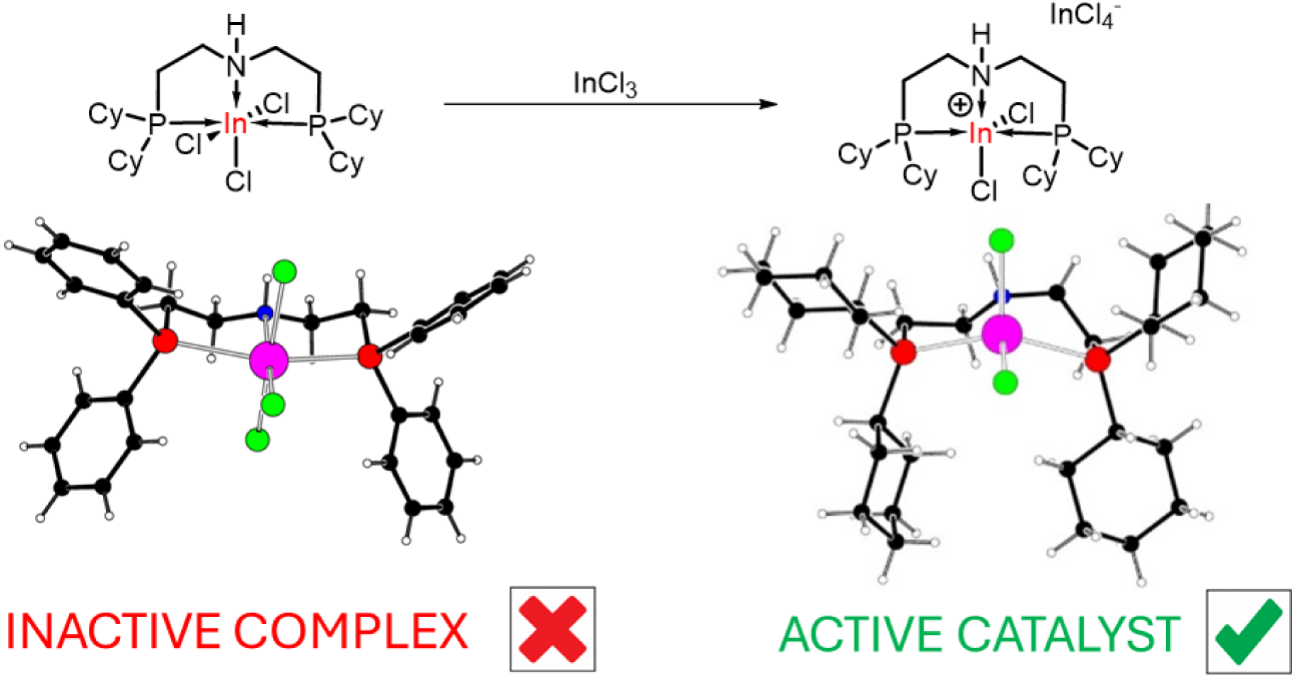
Removing an anionic ligand (Cl^–^, Br^–^, or I^–^) opens a coordination site on the complex, required for H_2_ activation, generating a catalytically active five-coordinate indium cation, as demonstrated by single-crystal XRD of inactive [In­(^Cy^PNP)­Cl_3_] and active [In­(^Cy^PNP)­Cl_2_]^+^.

Activation of the neutral complexes [In­(L^3^)­X_3_] with InX_3_ yields the active cationic species [In­(L^3^)­X_2_]^+^ and the mildly Lewis acidic [InX_4_]^−^ anion. While the [InX_4_]^−^ anion does not promote H_2_ activation nor affect CO_2_ hydrogenation,^22^ they are not innocent in the imine hydrogenation reaction. For instance, [In­(terpy)­Br_2_]­[InBr_4_] quantitatively hydrogenates the model substrate at 120 °C and 15 bar of H_2_ after 17 h (Table 1, entry 5), whereas [In­(terpy)­Br_2_]­[B­(C_6_F_5_)_4_] and [In­(terpy)­Br_2_]­[CHB_11_Cl_11_] only yield the desired product in 24–30%. Considering 1) the mild Lewis acidity of the [InX_4_]^−^ anions,^51^ 2) the absence of anionic effects on H_2_ activation and CO_2_ hydrogenation,^22^ 3) the necessity to activate imines by Brønsted or Lewis acids prior to their hydrogenation,^52^ and 4) the low concentration of protons in the current system, we tentatively propose that the [InX_4_]^−^ anion acts as an imine activator toward hydride transfer instead of or simultaneously to protons produced by H_2_ splitting (Scheme 1).

**Scheme 1:**
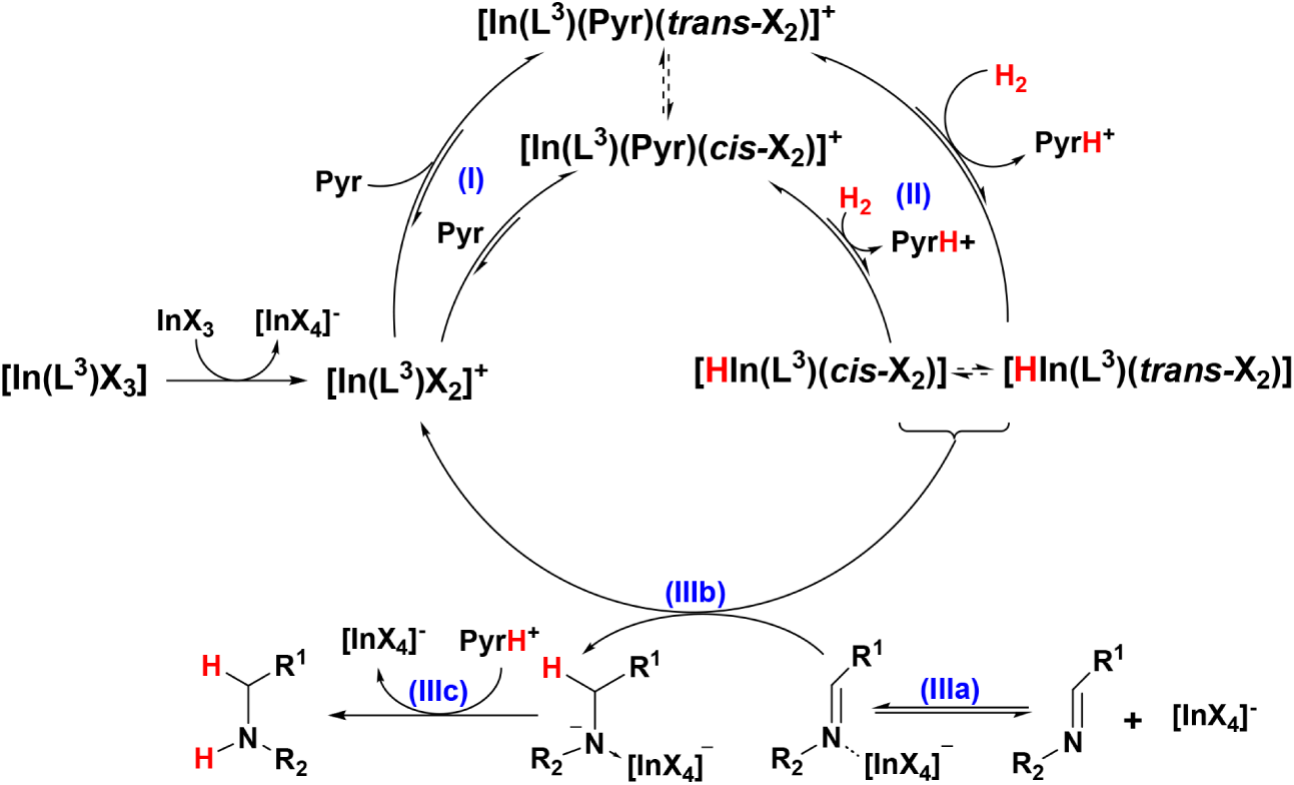
Catalytic Scheme of Imine Hydrogenation over [In­(L^3^)­X_2_]­[InX_4_] Catalysts with Pyrrolidine as the Cocatalyst

Based on our experimental findings on the activation of neutral indium complexes (Figure 5), thermodynamic analysis (Figure 4), the effect of the [InX_4_]^−^ anion, and published literature,^43,48,53–56^ we modified the classical FLP-type reaction mechanism to fit coordination compounds of indium (Scheme 1). Once the cationic complex is formed, pyrrolidine (pyr) reversibly binds to the [In­(L^3^)­X_2_]^+^ complex, yielding a *cis-* or *trans-*dihalide isomer [In­(L^3^)­(pyr)­X_2_]^+^ (Scheme 1, step **I**). Subsequent H_2_ activation (step **II**) is highly endergonic and, therefore, thermodynamically limits the reaction due to the unfavorable equilibrium for hydride formation. Moreover, except for [In­(^Ph/Dipp^NNN)­Cl_2_]^+^, LB coordination thermodynamically favors the binding site opposite to that required for hydride formation. For this reason, either the pyrrolidine-indium adduct or the indium-hydride must undergo isomerization for thermodynamically feasible reactivity. Such isomerization is commonly found in indium complexes by both dissociative and nondissociative twisting mechanisms so the isomers may interconvert during the reaction.^57–62^ In the final steps (**IIIa-c**), the imine substrate is likely activated by [InX_4_]^−^ (**IIIa**) toward hydride transfer (**IIIb**) and finally protonated (**IIIc**) affording the amine product and regenerating the active catalyst. NMR data and product inhibition analysis further indicate that the amine product does not bind to the indium catalyst at 120 °C and, thus, does not impede catalysis.

For its availability on a practical scale, [In­(terpy)­Br_2_]­[InBr_4_] was selected to assess the substrate scope of imine hydrogenation (Figure 6). Products could be isolated by acidic aqueous workup, which yielded the corresponding amines as water-soluble hydrochloride salts (see SI). Under the optimized conditions (120 °C, 15 bar H_2_ in 1,3-DCB), the model substrate *N*-*tert*-butyl-1-phenylmethanimine was hydrogenated to the corresponding amine **1** in 99% yield. However, gradually replacing the bulky *tert*-butyl group with smaller substituents, namely iso-propyl (**2**), phenyl (**3**) and methyl (**4**), decreased the yields to 40%, 40%, and 15%, respectively.

**Figure 6:**
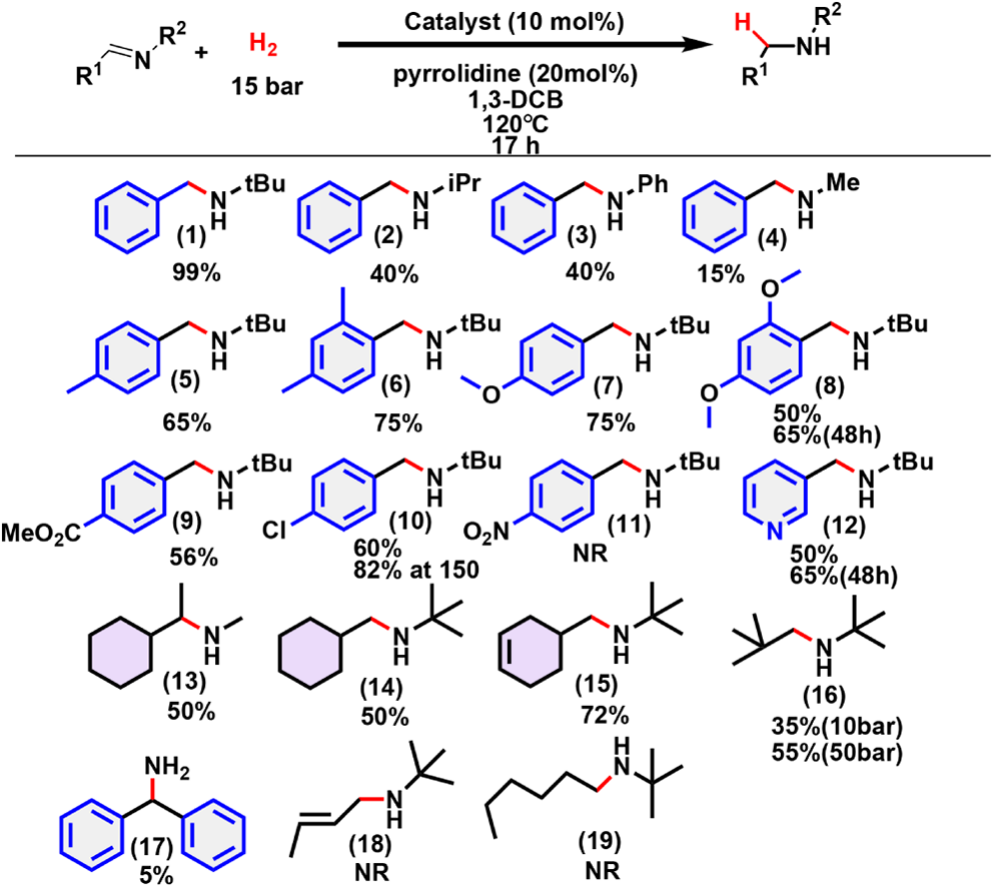
Substrate scope and yield of the target product. Reaction conditions: imine (1 mmol), [In­(terpy)­Br_2_]­[InBr_4_] (10 mol %), pyrrolidine (20 mol %) 1,3-DCB (4 mL), H_2_ (15 bar) for 17 h; yield was quantified by ^1^H NMR using CH_2_Br_2_ as the internal standard. Isolated compounds could be obtained as hydrochloride salts after aqueous acidic workup (see SI).

Introducing electron-donating substituents on the phenyl ring, such as methyl (**5** and **6**) or methoxy (**7**), at *para*- and *ortho*-positions slightly decreased the yields to 65–75%. In particular, the 2,4-dimethoxy substitution (**8**) decreased the yield to 50%. We attribute this decrease to steric hindrance near the imine moiety. Extending the reaction time to 2 days improved the yield of the product (**8**) to 65%.

Electron-withdrawing chloro- (**9**) and methoxycarbonyl (**10**) substituents afforded moderate yields of approximately 60%. This yield increased under more forcing conditions, such as 150 °C. At this temperature, the desired chloro-substituted amine (**10**) was produced in an 82% yield, whereas nitro-substituted substrates (**11**) remained unreactive, even under these conditions.

Heteroaromatic imines, including pyridyl-substituted examples, were also compatible, affording product (**12**) in 50% yield after 17 h and 65% after 2 days. Cyclohexyl- and cyclohexenyl-substituted imines underwent hydrogenation, delivering the corresponding amines in 50% (**13** and **14**) and 72% (**15**) yields, respectively, without concomitant reduction of the alkene moiety. In contrast, open-chain aliphatic imines provided slightly lower yields of the corresponding amines (**16**), and imines with longer open-chain aliphatic substituents proved unreactive (**18** and **19**) presumably due to the steric hindrance imposed by the rotational flexibility of the chain in comparison to the rigid ring substituents of substrates **13–15**.

## Conclusion

In summary, we developed a series of thermally and hydrolytically stable cationic indium complexes in the form [In­(L^3^)­X_2_]­[InX_4_], bearing tridentate NNN-, PNN-, and PNP-pincer ligands, which catalyze imine hydrogenation under 15 bar H_2_ and 120 °C in the presence of pyrrolidine. Their catalytic activity inversely correlates with the Δ*G* of H_2_ activation leading to the more thermodynamically favored hydride formation and strongly depends on the *cis–trans* isomerism of LB and hydride binding. Broadly speaking, LB binding favors the isomer opposite to that required for hydride formation, so the indium-LB or indium-hydride must undergo isomerization for thermodynamically feasible reactivity. Indium complexes outperform their lighter group-13 Al and Ga congeners thanks to more favorable hydride formation thermodynamics rather than higher Lewis acidity. Heavier halide substituents enhance hydrogenation activity (I^–^ > Br^–^ > Cl^–^) by weakening LB binding with minor effects on hydride stability, thereby improving the overall thermodynamics of H_2_ activation. Moreover, the [InX_4_]^−^ anions greatly enhance the catalysts’ activity presumably by activating the imine substrate toward hydride transfer instead of or simultaneously to protons produced by H_2_ splitting. Overall, these results address key limitations associated with the thermal and hydrolytic instability of classical heavier main-group LAs and demonstrate that further development of indium-based hydrogenation catalysts will require stabilizing indium-hydrides while carefully controlling LB access, and interactions with the complex through ligand design while addressing substrate activation toward hydride transfer.

## Experimental Section

### General Procedure for Catalytic Hydrogenation of Imines

**Caution!** Hydrogen is classified as a GHS flammable gas, Category 1. Working with pressurized H_2_ may lead to explosions. All reactions were performed in specialized high-pressure equipment certified for use with H_2_ gas and within blast-resistant enclosures.

In a glovebox, catalyst (0.10 mmol) and imine (1 mmol) were mixed in 1,3-DCB (4 mL) in a steel autoclave. The autoclave was then sealed and purged 3 times with 15 bar of H_2_. The temperature and stirring rate were set using the SpecView program on a Parr 5000 series multireactor system. *T* = 0 was defined as the time when the heating started. The heating was turned off 1 h before the end of the stated reaction time and allowed to cool under pressure over the course of the remaining 1 h of the test; i.e., for a reaction time of 17 h, the heating was turned off after 16 h and the reaction was depressurized after the 17 h mark. Dibromomethane (1 mmol) was added to the reactor, stirred, and an aliquot was taken for ^1^H NMR analysis in CDCl_3_. The conversion of imine and the yield of the product were quantified by ^1^H NMR analysis with the added dibromomethane as the internal standard. Other reaction products were quantified by their respective −CH_2_– signal in ^1^H NMR. The product structures were confirmed by ^1^H NMR and ESI mass spectrometry after product extraction with 1 M aqueous HCl solution.

### General Procedure for DFT Thermochemistry Calculations

Thermochemistry calculations were performed using the Gaussian 16 package,^63^ employing the M06–2X method^64^ together with the def2-SVP basis set for the initial optimization of the relevant structures. Reoptimization at the M06–2X/def2-TZVP level of theory was performed to obtain more precise Gibbs free energies and to confirm the absence of imaginary frequencies for calculated minima. Solvation was included using the self-consistent reaction field (SCRF) approach and employing IEFPCM^65^ with toluene parameters.

## Supplementary Material



## Notes

Catalytic reaction optimization (temperature, pressure, added Lewis base), catalyst characterization (NMR, MALDI-MS, single-crystal XRD data), computational details, substrate scope analysis, and details (PDF)

## ABBREVIATIONS

L^3^: tridentate ligand
terpy: 2,2′;6′,2″-terpyridine
^Dipp^NNN: 2,6-bis­(2,6-diisopropylphenyliminomethyl)­pyridine
^Ph^NNN: 2,6-bis­(2,6-phenyliminomethyl)­pyridine
^tBu^PN^Et^N: 2-((di-*tert*-butylphosphinomethyl)-6-diethylaminomethyl)­pyridine
^Cy^PNP: bis­(2-(dicyclohexylphosphaneyl)­ethyl)­amine
