# Supplementary material for: Cis–Trans Isomerism and Hydride Thermodynamics Govern H2 Activation and Hydrogenation Activity in Indium(III) Pincer Complexes
Source: Inorg Chem. 2026 Jul 15;65(29):17108–17. doi: 10.1021/acs.inorgchem.6c02395 (PMC13418170; doi:10.1021/acs.inorgchem.6c02395)
Supplement: Supplementary file 1 [file ic6c02395_si_001.pdf]

Supporting Information

Cis-Trans Isomerism and Hydride Thermodynamics Govern H_2 Activation and Hydrogenation activity in Indium(III) Pincer Complexes

Pritha Saha,[a] Gabriela Gastelu,[b,c] Leandro Mena,[b,c], Róbert Gyepes,[d] Jorge G. Uranga[b,c] and Martin Hulla*[a]*

[a] Faculty of Science, Department of Inorganic Chemistry, Charles University, Albertov 6, 128 00 Praha 2, Czech Republic. E-mail: Martin.Hulla@natur.cuni.cz

[b] Departamento de Química Orgánica, Facultad de Ciencias Químicas, Universidad Nacional de Córdoba, X5000HUA Córdoba, Argentina. E-mail: jorge.uranga@unc.edu.ar

[c] Instituto de Investigaciones en Físico-Química Córdoba, Universidad Nacional de Córdoba (INFIQC-CONICET), Córdoba, 5000, Argentina.

[d] Department of Chemistry, Faculty of Education, J. Selye University, Bratislavská 3322, 945 01 Komárno, Slovakia

Contents

General procedures ... 4

1. $[E(terpy)X_2][EX_4]$ (E = Al, Ga, In and X = Cl, Br, I) ... 4

  1.1 $[Al(terpy)Cl_2][AlCl_4]$ ... 4

  1.2 $[Ga(terpy)Cl_2][GaCl_4]$ ... 4

  1.3 $[Ga(terpy)Br_2][GaBr_4]$ ... 5

  1.4 $[In(terpy)Cl_2][InCl_4]$ ... 5

  1.5 $[In(terpy)Br_2][InBr_4]$ ... 8

  1.6 $[In(terpy)I_2][InI_4]$ ... 10

2. $[In(^{Cy}PNP)Cl_2][InCl_4]$ ... 11

3. $[In(^{tBu}PN^{Et}N)Cl_2][In_2Cl_6]$ ... 14

4. $[In(^{R}NNN)Cl_2]^+$ ... 16

  4.1 [2,6-Bis[1-(phenylimino)ethyl]pyridine] ligand ... 16

  4.2 $[In(^{Ph}NNN)Cl_2][InCl_4]$ ... 16

  4.3 [2,6-Bis[1-(2,6-diisopropylphenylimino)ethyl]pyridine] ligand ... 18

  4.4 $[In(^{Dipp}NNN)Cl_2][InCl_4]$ ... 18

5. $[In(terpy)Br_2][B(C_6F_5)_4]$ and $[In(terpy)Br_2][CHB_{11}Cl_{11}]$ ... 21

  $[In(terpy)Br_2][CHB_{11}Cl_{11}]$ ... 21

  $[In(terpy)Br_2][B(C_6F_5)_4]$ ... 21

6. GB-acidity measurement ... 21

6.1 ^{31}P NMR shifts for GB-acidity measurements ... 22

  $[In(^{Cy}PNP)Cl_2]^+$ ... 22

  $[In(terpy)Cl_2]^+$ ... 22

  $[Al(terpy)Cl_2]^+$ ... 23

  $[Ga(terpy)Cl_2]^+$ ... 23

  $[In(terpy)Br_2]^+$ ... 24

  $[In(terpy)I_2]^+$ ... 25

  $[In(^{Dipp}NNN)Cl_2]^+$ ... 25

  $[In(^{Ph}NNN)Cl_2]^+$ ... 26

7. Catalyst testing ... 26

  7.1 General test result ... 26

  7.2 Effect of Lewis acid ... 28

  7.3 H_2 pressure ... 29

  7.4 Reaction temperature ... 29

  7.5 Lewis base ... 30

  7.6 Reaction solvent ... 30

8. H/D scrambling experiments 31
9. Crystallographic data 32
9.1 [In(terpy)Cl_2][$InCl_4$] 32
9.2 [In(terpy)Br_2][$InBr_4$] 33
9.3 [In(terpy)I_2][InI_4] 33
9.4 [In(^{Cy}PNP)Cl_2][$InCl_4$] 34
9.5 [In(^{Dipp}NNN)(MeCN)Cl_2][$InCl_4$] 35
9.6 [In(^{tBu}PN^{Et}N)Cl_2][In_2Cl_6] 35
9.7 [In(^{Cy}PNP)Cl_3] 36
10. Computational details 37
10.1 HIA and FIA calculations 37
10.2 Energy profiles for LA-hydride formation 38
10.4 Relevant Molecular Orbitals 40
11. Substrate scope 41
11.1 General procedure for imine synthesis 41
11.2 General procedure for amine extraction after hydrogenation 41
12. References 44

General procedures

Solvents and chemicals were purchased from commercial suppliers (Abcr, Merck, TCI, Lachner, Acros). Unless otherwise specified, solvents were dried using automatic drying system PureSolv MD5, or by storing upon activated molecular sieves and degassed by freeze-pump-thaw before use. Glassware was dried and stored in an oven heated up to 180 °C overnight. NMR spectra were measured on Bruker AVANCEIII (400 MHz) at 25 °C. Chemical shifts for ^{1}H are given in δ relative to tetramethylsilane (TMS) and are referenced to residual proton signal in the NMR solvent ($CDCl_3$: δ = 7.26 ppm, CD_3CN: δ = 1.98). Chemical shifts for ^{13}C are given in δ relative to TMS and are referenced to the carbon resonances in the solvent ($CDCl_3$: δ = 77.0 ppm, CD_3CN: δ = 116.43, 1.89). Ligands bis[2-(dicyclohexylphosphino)ethyl]amine (^{Cy}PNP) , N-((6-((diethylphosphaneyl)methyl)pyridin-2-yl)methyl)-N-ethylethanamine (^{Et}P^{tBu}NN), 2,6-Bis[1-(phenylimino)ethyl]pyridine(L^1) and 2,6-Bis[1-(2,6-di-i-propylphenylimino)ethyl]pyridine(L^1-iPr) were purchased from commercial supplier and used without further purification. Mass spectrometry measurements were provided by Service centrum of Mass spectrometry at the Department of Chemistry, Charles University. Ionization method used was ESI positive and the spectra were measured in MeOH or in the solid state by MALDI in the absence of an ionization matrix. Optimization of catalysts was performed at least in duplicate but generally in triplicate and analyzed by NMR with dibromomethane as the internal standard and structures of amine products confirmed by ESI-MS.

1. [E(terpy)X_2][EX_4] (E = Al, Ga, In and X = Cl, Br, I)

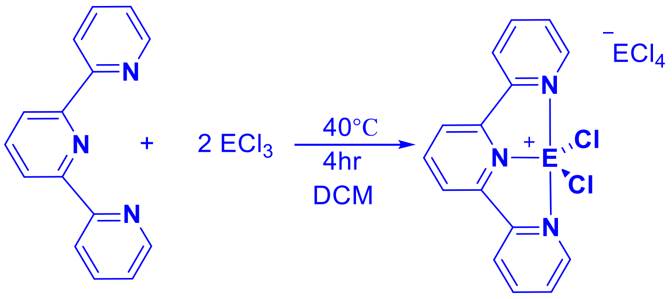

The compounds [E(terpy)Cl_2][ECl_4] (E = Al, Ga, In) were synthesized according to our procedure using $AlCl_3$, $GaCl_3$ or $InCl_3$ respectively.[1] Crystal suitable for single-crystal XRD were grown from acetonitrile solutions by vapor diffusion of heptane (see chapter 9)

1.1 [Al(terpy)Cl_2][$AlCl_4$]

^{1}H NMR (400 MHz, CH_3CN/$CDCl_3$ for lock) δ 9.35 (dt, *J* = 5.4, 1.2 Hz, 2H), 8.60 (dd, *J* = 4.4, 3.4 Hz, 6H), 8.45 (td, *J* = 7.8, 1.6 Hz, 2H), 7.98 (ddd, *J* = 7.7, 5.4, 1.2 Hz, 2H); **^{13}C NMR** (101 MHz, CD_3CN) δ 123.70, 123.78, 128.53, 143.33, 145.15, 147.95; **MS(MALDI-TOF)** = Calculated for $[C_{15}H_{11}AlCl_2N_3]^+$ 330.01, found 330.03 Yield=50%

1.2 [Ga(terpy)Cl_2][$GaCl_4$]

^{1}H NMR (400 MHz, CD_3CN) δ 9.05 (ddd, *J* = 5.4, 1.5, 0.8 Hz, 2H), 8.84 – 8.76 (m, 3H), 8.72 – 8.67 (m, 2H), 8.56 (td, *J* = 7.9, 1.6 Hz, 2H), 8.11 (ddd, *J* = 7.7, 5.3, 1.1 Hz, 2H); **^{13}C NMR** (101 MHz, CD_3CN) δ 123.66, 124.65, 129.73, 143.38, 144.32, 145.98, 147.53, 147.79; **ESI-MS** = Calculated for aqua adduct [$C_{15}H_{13}Cl_2GaN_3O$] + 391.97, found 391.28. Yield=70%

1.3 [Ga(terpy)Br_2][$GaBr_4$]

^{1}H NMR (400 MHz, CD_3CN) δ 9.07 (d, *J* = 5.4 Hz, 2H), 8.87 – 8.76 (m, 3H), 8.68 (dt, *J* = 8.1, 1.1 Hz, 2H), 8.54 (td, *J* = 7.8, 1.6 Hz, 2H), 8.11 (ddd, *J* = 7.6, 5.4, 1.2 Hz, 2H). **^{13}C NMR** (101 MHz, CD_3CN) δ 147.74, 147.20, 144.23, 143.02, 129.67, 124.71, 123.66; **ESI-MS** = Calculated for aqua adduct [$C_{15}H_{11}Br_2GaN_3$] + 461.86 (isotopes: + 459.86, 463.85), found +461.68 (isotopes +459.83, 463.68).

1.4 [In(terpy)Cl_2][$InCl_4$]

^{1}H NMR (400 MHz, Acetonitrile-d_3) δ 9.01 (dt, *J* = 5.2, 1.3 Hz, 2H), 8.77 (ddd, *J* = 8.1, 2.6, 1.1 Hz, 4H), 8.68 (dd, *J* = 8.8, 7.1 Hz, 1H), 8.55 (td, *J* = 7.9, 1.6 Hz, 2H), 8.08 (ddd, *J* = 7.7, 5.2, 1.1 Hz, 2H). **^{13}C NMR** (101 MHz, Acetonitrile-d_3) δ 148.05, 145.03, 143.64, 129.05, 124.94, 124.78. **MS(MALDI-TOF)**-Calculated for : [$C_{15}H_{11}Cl_2InN_3$]$^+$ 417.94, found 417.93;Yield=77%; **sc-XRD** see chapter 9.1

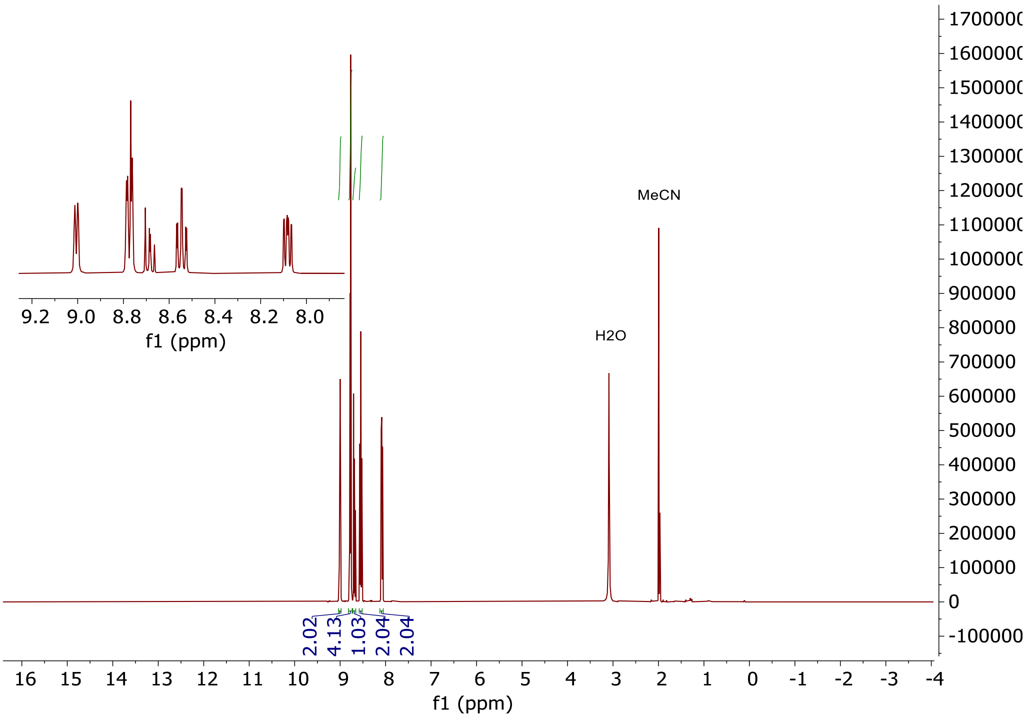

Figure S1: ^{1}H NMR (CD_3CN, 400MHz) of [In(terpy)Cl_2][$InCl_4$]

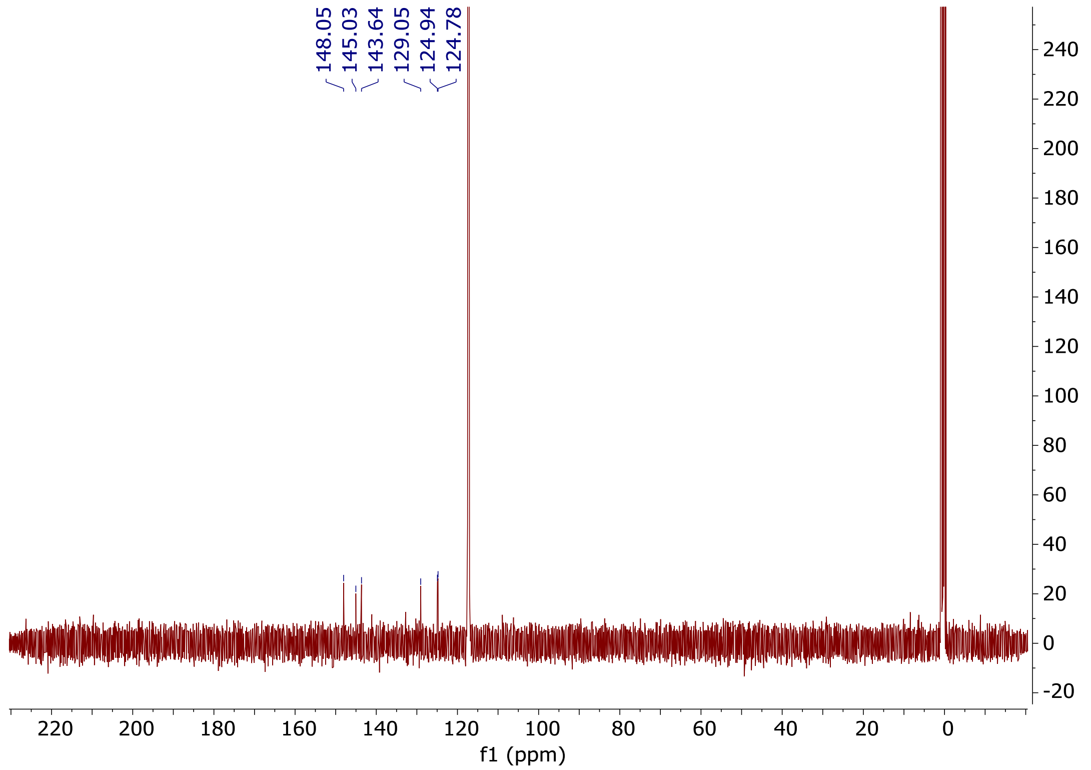

Figure S2 ^{13}C NMR (CD_3CN, 101 MHz) of $[In(terpy)Cl_2][InCl_4]$

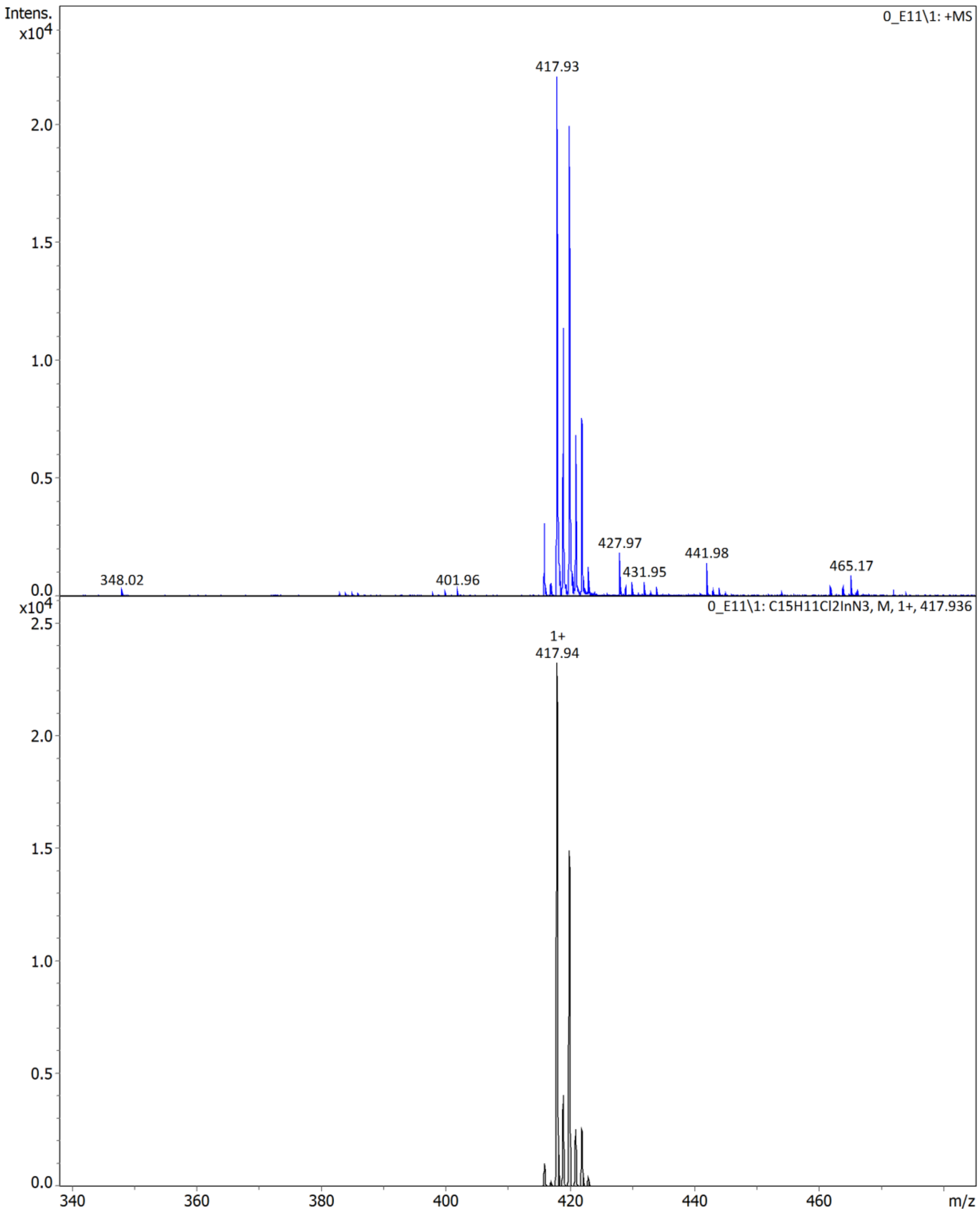

Figure S3: MS(MALDI-TOF) of $[In(terpy)Cl_2][InCl_4]$

1.5 [In(terpy)Br_2][$InBr_4$]

^{1}H NMR (400 MHz, CD_3CN) δ 9.04 (ddd, *J* = 5.2, 1.7, 0.9 Hz, 2H), 8.78 (tt, *J* = 7.3, 1.1 Hz, 4H), 8.70 (dd, *J* = 8.8, 7.0 Hz, 1H), 8.55 (td, *J* = 7.9, 1.6 Hz, 2H), 8.10 (ddd, *J* = 7.7, 5.2, 1.1 Hz, 2H); **^{13}C NMR** (101 MHz, CD_3CN) δ 124.86, 125.15, 129.06, 143.74, 144.43, 144.83, 145.11, 147.79; **MS(MALDI-TOF)** = Calculated for $[C_{15}H_{11}Br_2InN_3]^+$ 507.83 , found 507.82; Yield=75%; **sc-XRD** see chapter 9.2

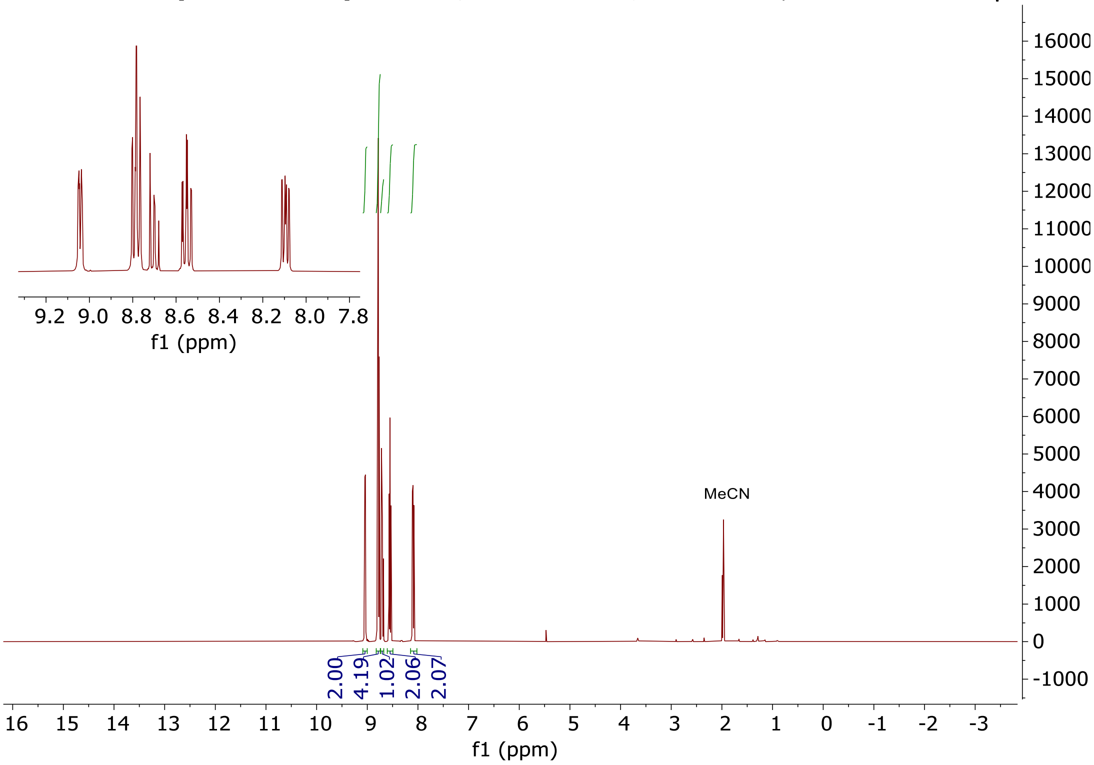

Figure S4: ^{1}H NMR (400 MHz, CD_3CN) of [In(terpy)Br_2][$InBr_4$]

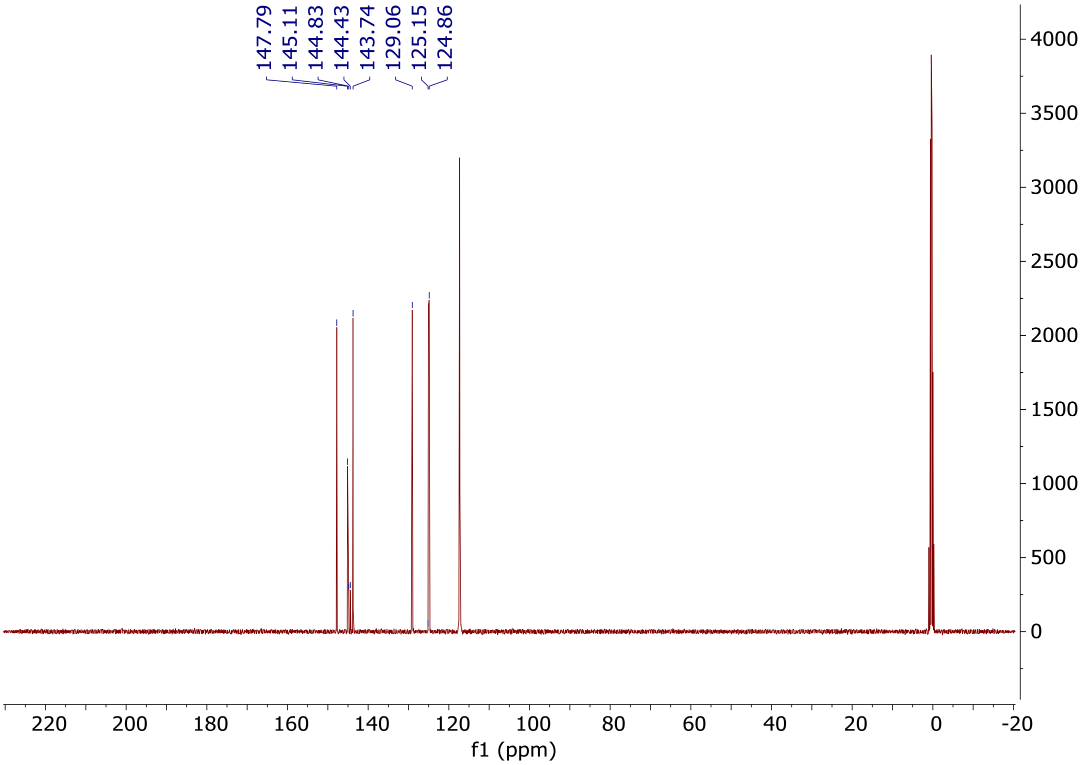

Figure S5: ^{13}C NMR (101 MHz, CD_3CN)of [In(terpy)Br_2][$InBr_4$]

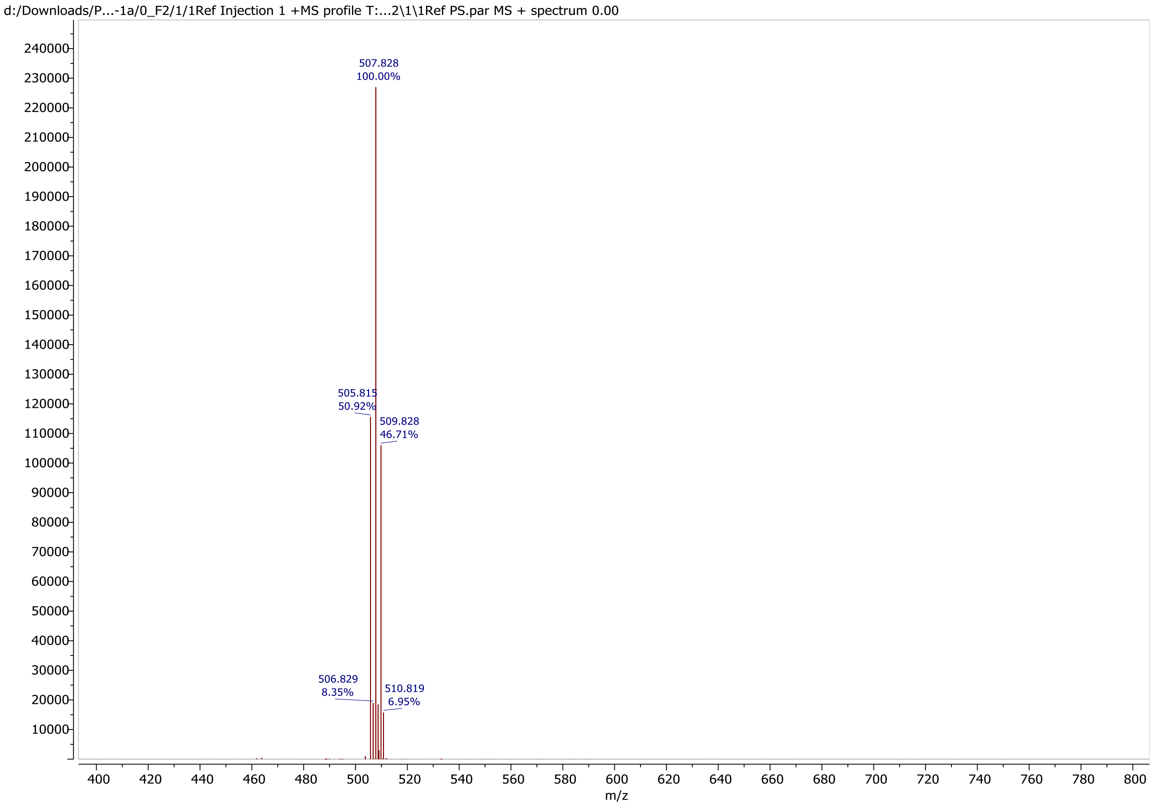

Figure S6: MS(MALDI-TOF) of [In(terpy)Br_2][$InBr_4$]

1.6 [In(terpy)I_2][InI_4]

^{1}H NMR (400 MHz, CD_3CN) δ 9.06 (ddd, *J* = 5.2, 1.6, 0.9 Hz, 1H), 8.83 – 8.67 (m, 3H), 8.54 (td, *J* = 7.9, 1.6 Hz, 1H), 8.10 (ddd, *J* = 7.7, 5.3, 1.2 Hz, 1H); **^{13}C NMR** (101 MHz, CD_3CN) δ 124.78, 125.15, 129.04, 143.80, 144.14, 145.02, 145.24, 147.33; **MS(MALDI-TOF)** - Calculated for $[C_{15}H_{11}I_2InN_3]^+$ 601.81, found 601.81; Yield=75%; **sc-XRD** see chapter 9.3

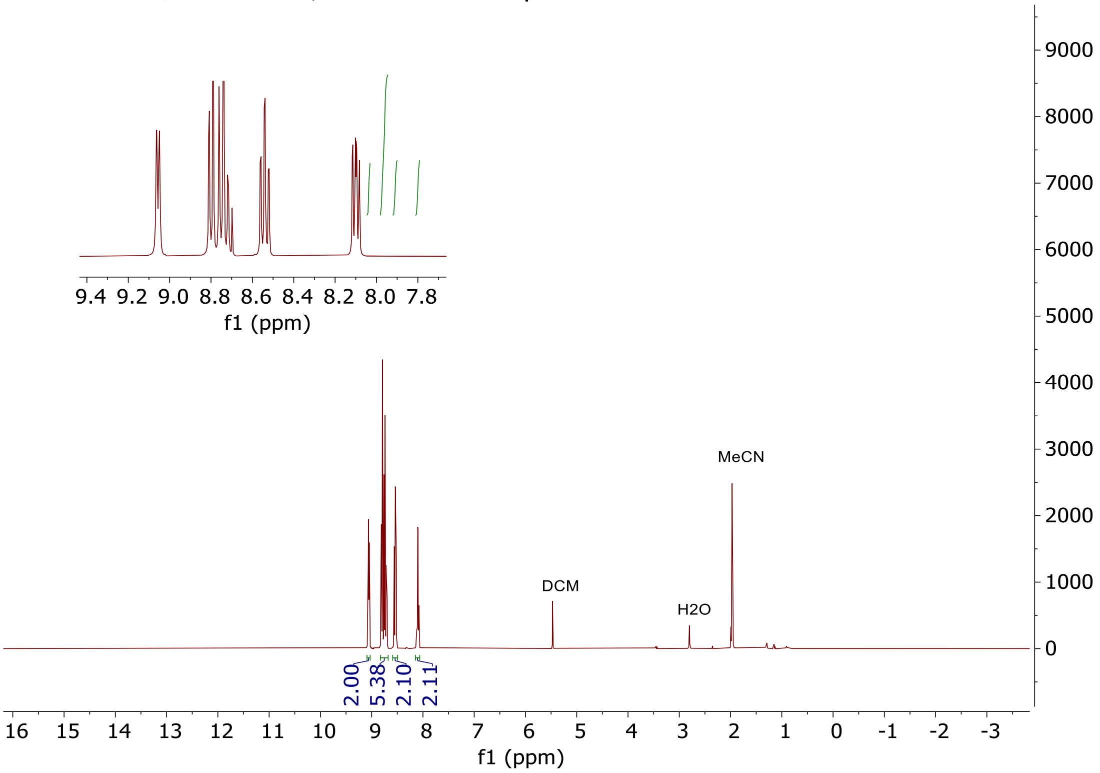

Figure S7: ^{1}H NMR (400 MHz, CD_3CN) of [In(terpy)I_2][InI_4]

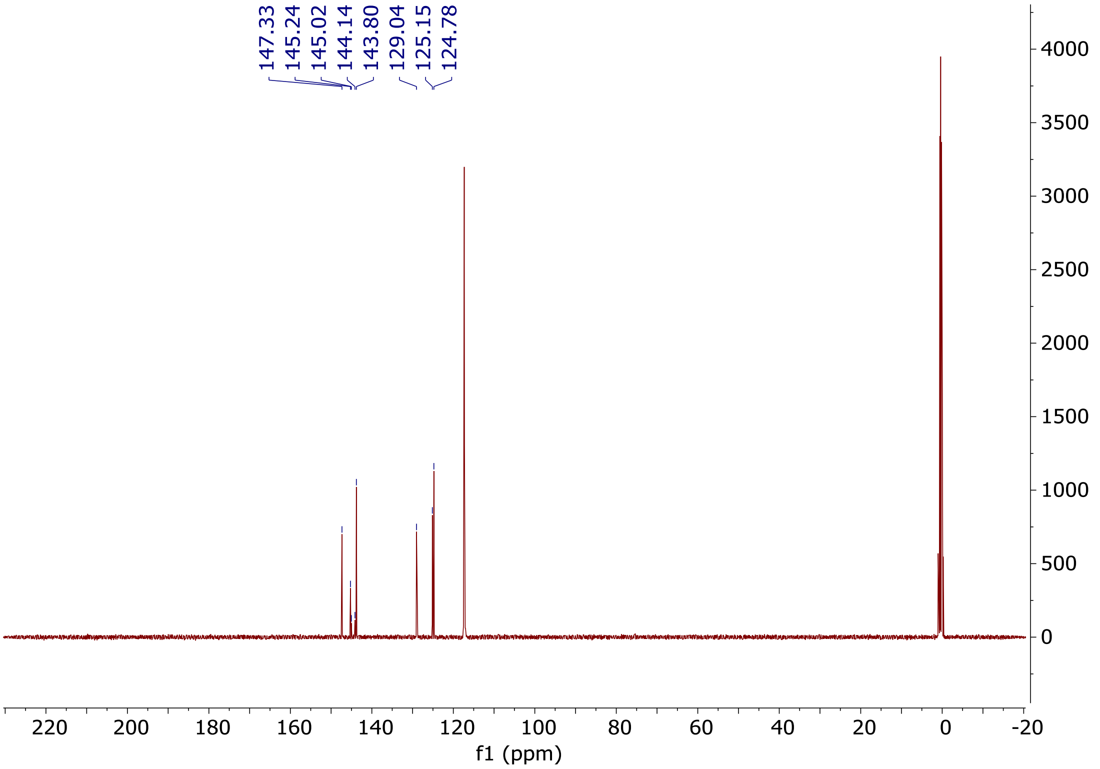

Figure S8: ^{13}C NMR (101 MHz, CD_3CN) of [In(terpy)I_2][InI_4]

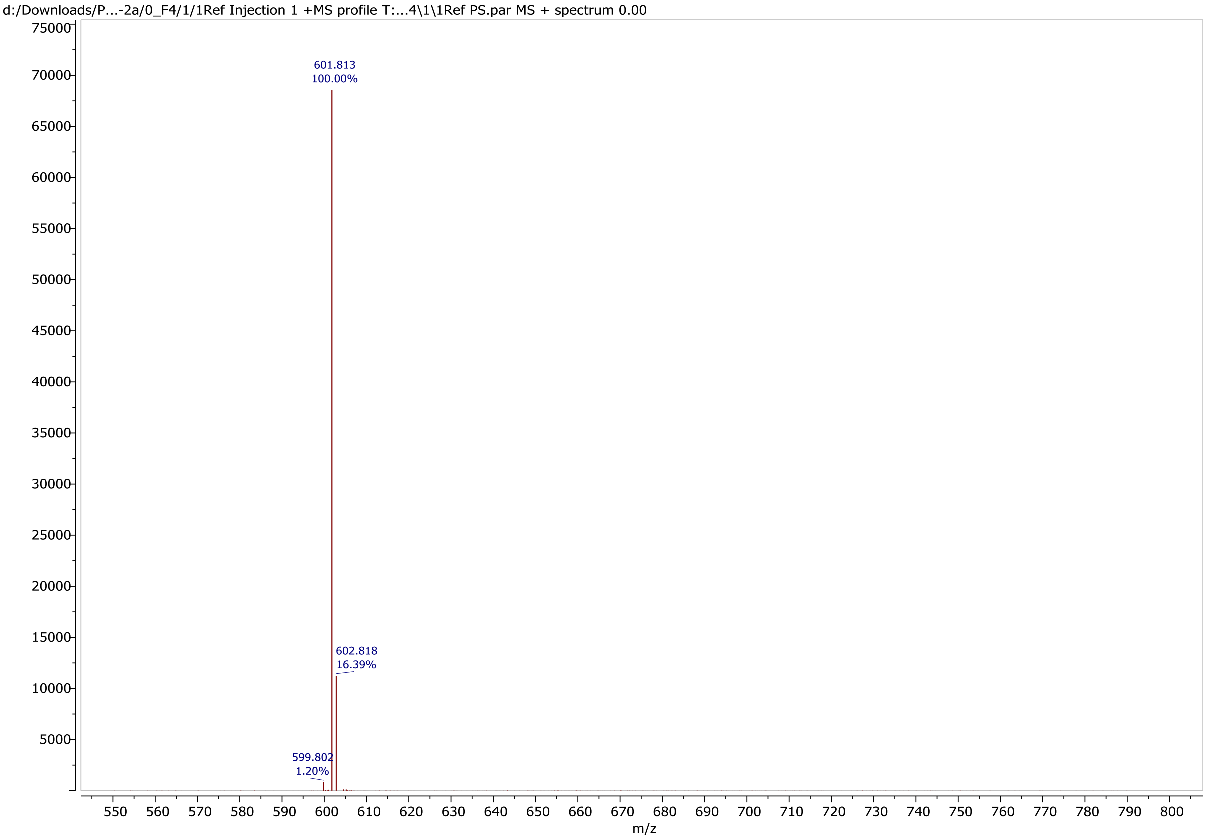

Figure S9: MS(MALDI-TOF) of $[In(terpy)I_2][InI_4]$

2. $[In(^{Cy}PNP)Cl_2][InCl_4]$

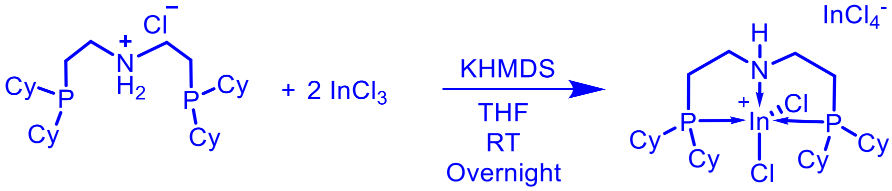

^{Cy}PNP.HCl (184 mg, 0.37 mmol) was dissolved in THF (10 mL) followed by dropwise addition of $InCl_3$ (176 mg, 0.79 mmol) solution in THF (10 mL). After stirring for 5 min KHMDS (152 mg, 0.76 mmol) was added and the reaction mixture was stirred overnight at room temperature. The solvent was removed under reduced pressure and the residue dissolved in DCM, filtered and evaporated. Recrystallization from toluene and washing with pentane afforded the product as a white powder.

^{1}H NMR (300 MHz, tol-d_8) δ 3.62 (m, 4H), 2.79 (m, 4H), 2.46 – 1.73 (m, 41H);**^{13}C NMR** (101 MHz, $CDCl_3$) δ 19.10, 25.94, 27.69, 28.65, 29.10, 29.66, 33.13, 34.74, 42.84; **^{31}P NMR** (121 MHz, $CDCl_3$) δ -16.59; **MS(MALDI-TOF)** = Calculated for $[C_{28}H_{54}Cl_2InNP]^+$ 620.24, found 620.29; Yield=50%; **sc-XRD** see chapter 9.4

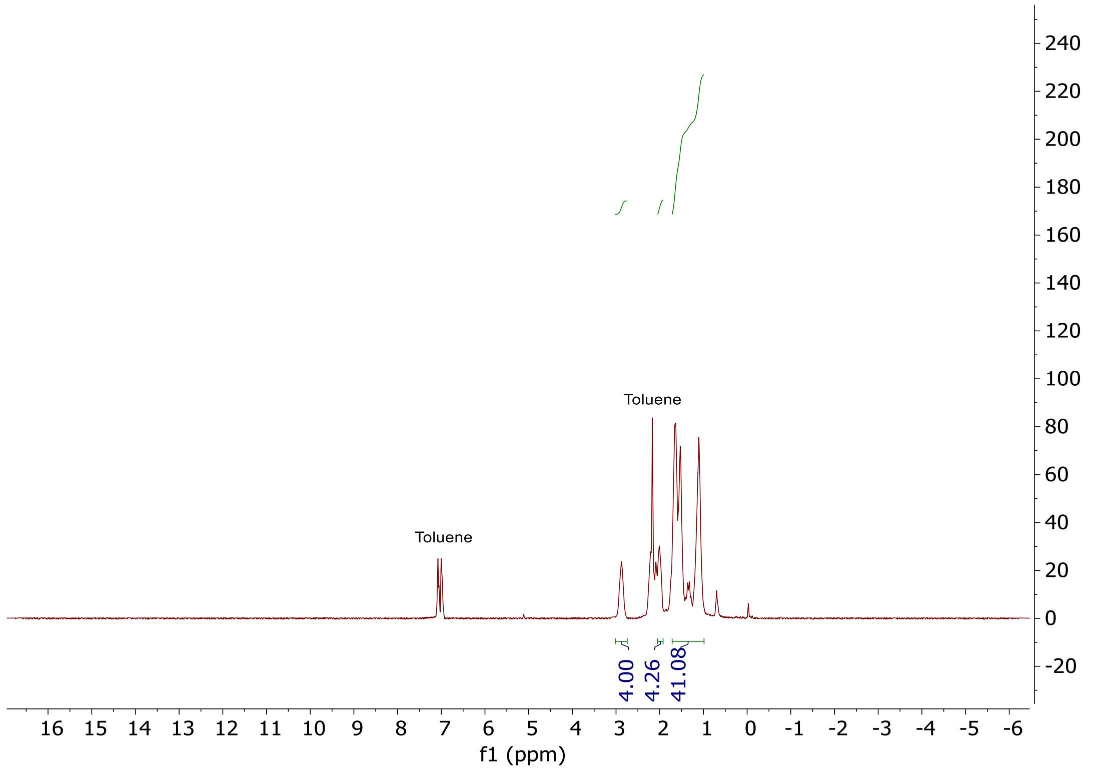

Figure S10: ^{1}H NMR (300 MHz, tol-d_8) of [In(^{Cy}PNP)Cl_2][$InCl_4$]

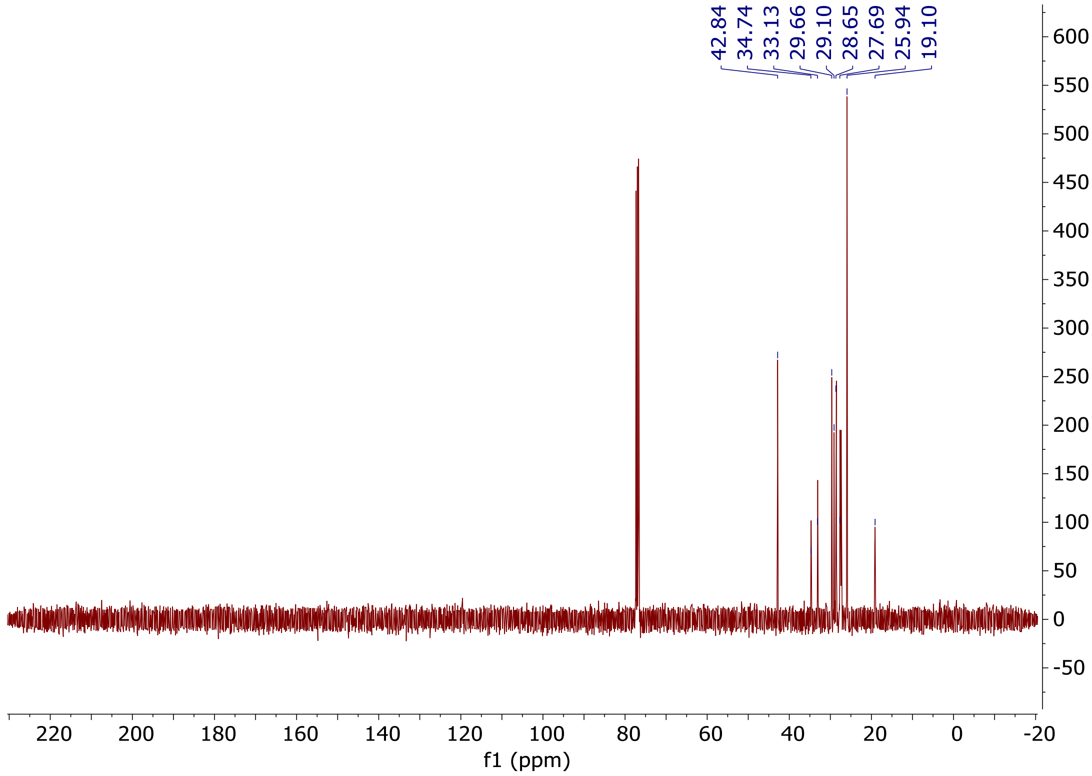

Figure S11: ^{13}C NMR (101 MHz, $CDCl_3$) of [In(^{Cy}PNP)Cl_2][$InCl_4$]

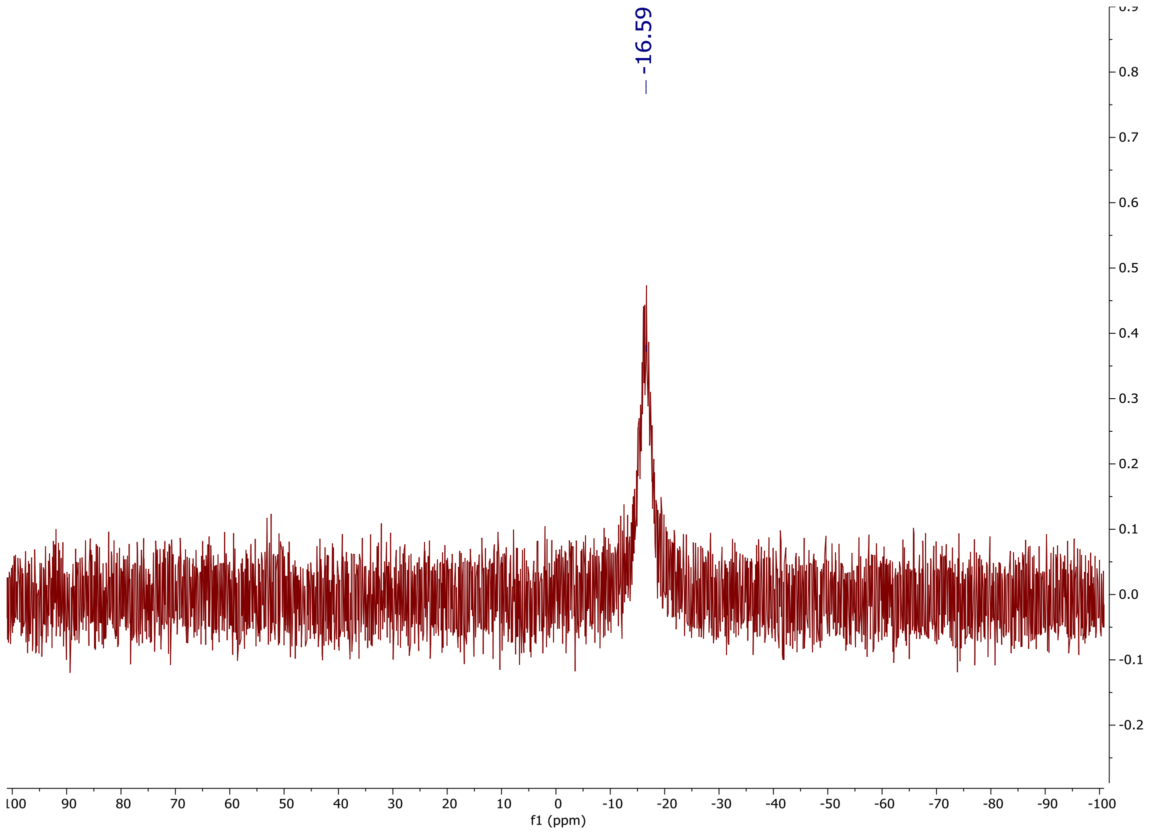

Figure S12: ^{31}P NMR (121 MHz, $CDCl_3$) of [In(^{Cy}PNP)Cl_2][$InCl_4$]

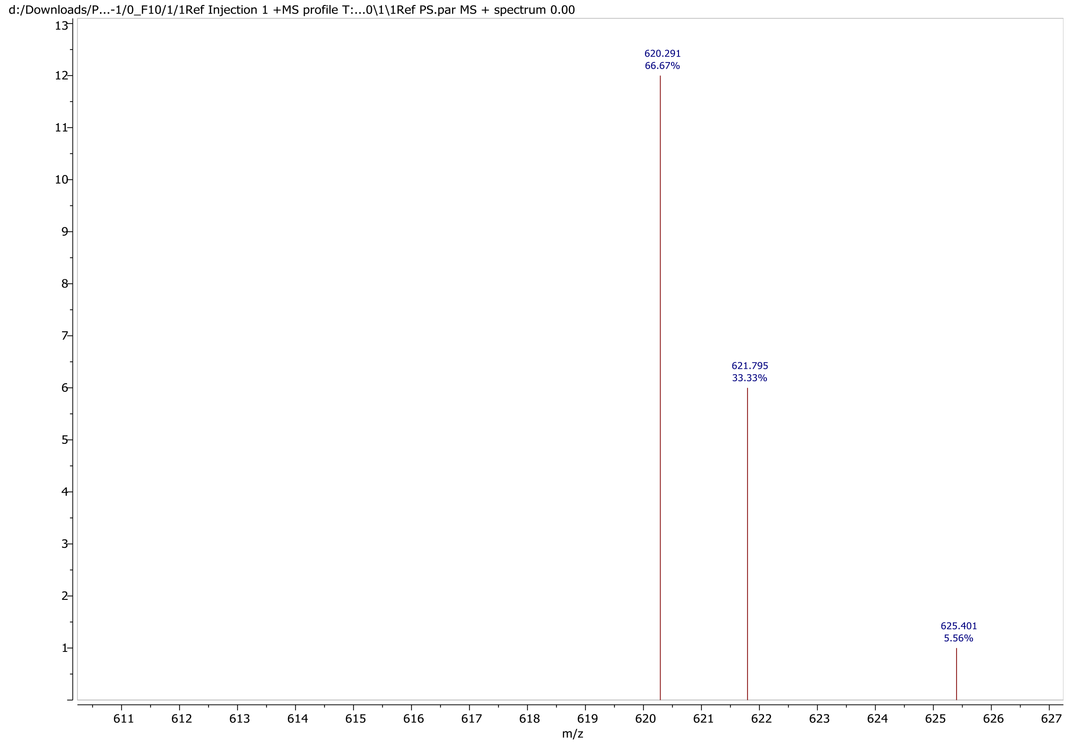

Figure S13: MS(MALDI-TOF) of [In(CyPNP)Cl_2]]$InCl_4$]

3. [In(^{tBu}PN^{Et}N)Cl$_2$][In$_2$Cl$_6$]

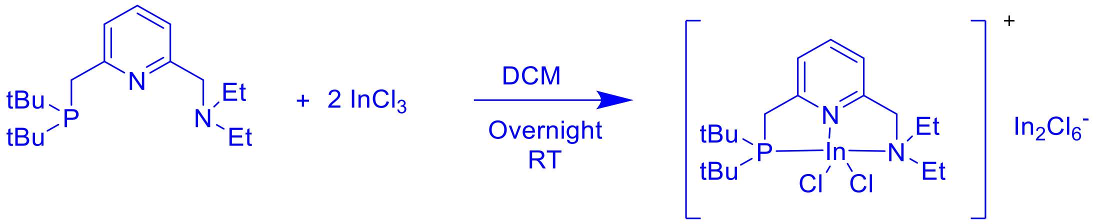

To a solution of the ^{tBu}PN^{Et}N (270 µl, 0.78 mmol) in DCM (10 mL) was added $InCl_3$ (350 mg, 1.58 mmol) resulting immediately in an orange solution. Following overnight stirring, the orange solution was evaporated and washed with pentane and toluene to yield the product in 56% yield.

^{1}H NMR (300 MHz, CD_3CN) δ 8.21 (t, J = 7.8 Hz, 1H), 7.76 (d, J = 7.9 Hz, 1H), 7.60 (d, J = 7.7 Hz, 1H), 4.33 (s, 2H), 3.84 (d, J = 10.3 Hz, 2H), 3.21 (q, J = 7.3 Hz, 4H), 1.49 (dd, J = 15.6, 2.1 Hz, 19H), 1.28 (t, J = 7.2 Hz, 7H); **^{13}C NMR** (101 MHz, $CDCl_3$) δ 9.40, 22.34, 29.94, 35.52, 46.86, 56.84, 123.39, 127.00, 142.15, 151.42; **^{31}P NMR** (121 MHz, CD_3CN) δ 15.57; **ESI-MS+** (m/z) = Calculated for $[C_{19}H_{35}Cl_2InN_2P]^+$ 507.10, found 507.16 (additional signals: 523.08 complex with oxidized ligand $[C_{19}H_{35}Cl_2InN_2PO]^+$, 429.16 hydrolyzed and methanolized complex with partial fragmentation $[C_{16}H_{31}InN_2O_2P]^+$, 339.30 oxidized ligand $[C_{19}H_{36}N_2PO]^+$, and 323.3 free ligand $[C_{19}H_{36}N_2P]^+$); Yield=56%; **sc-XRD** see chapter 9.6

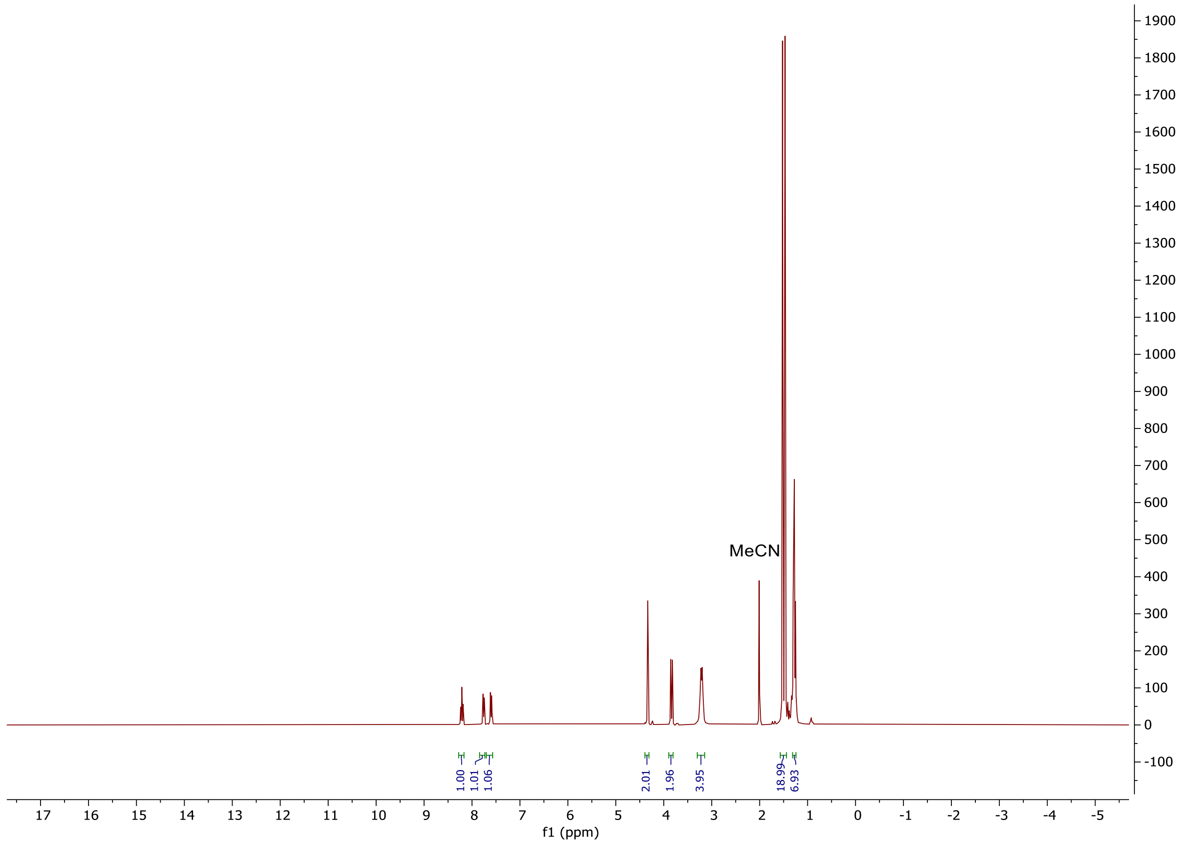

Figure S14: ^{1}H NMR (300 MHz, CD_3CN) of [In(^{tBu}PN^{Et}N)Cl$_2$][In$_2$Cl$_6$]

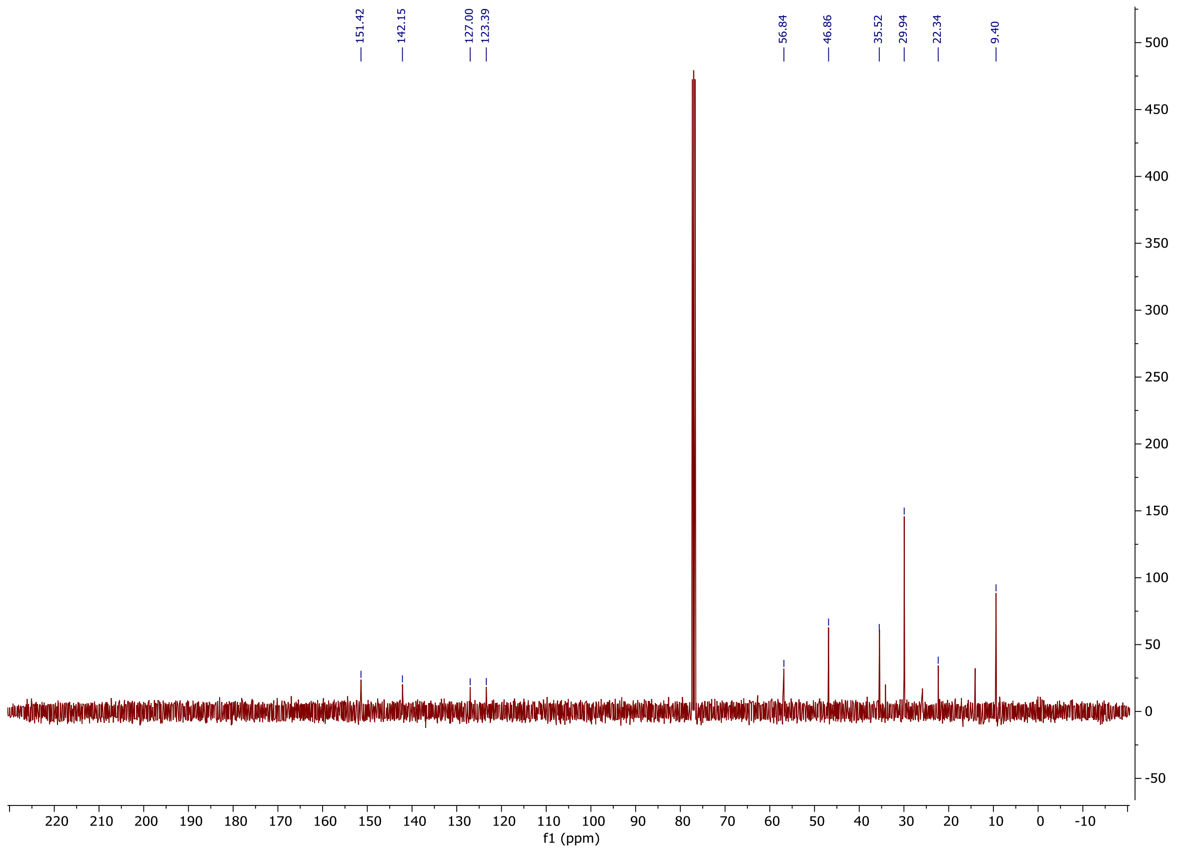

Figure S15: ^{13}C NMR (101 MHz, $CDCl_3$) $[In(^{tBu}PN^{Et}N)Cl_2][In_2Cl_6]$

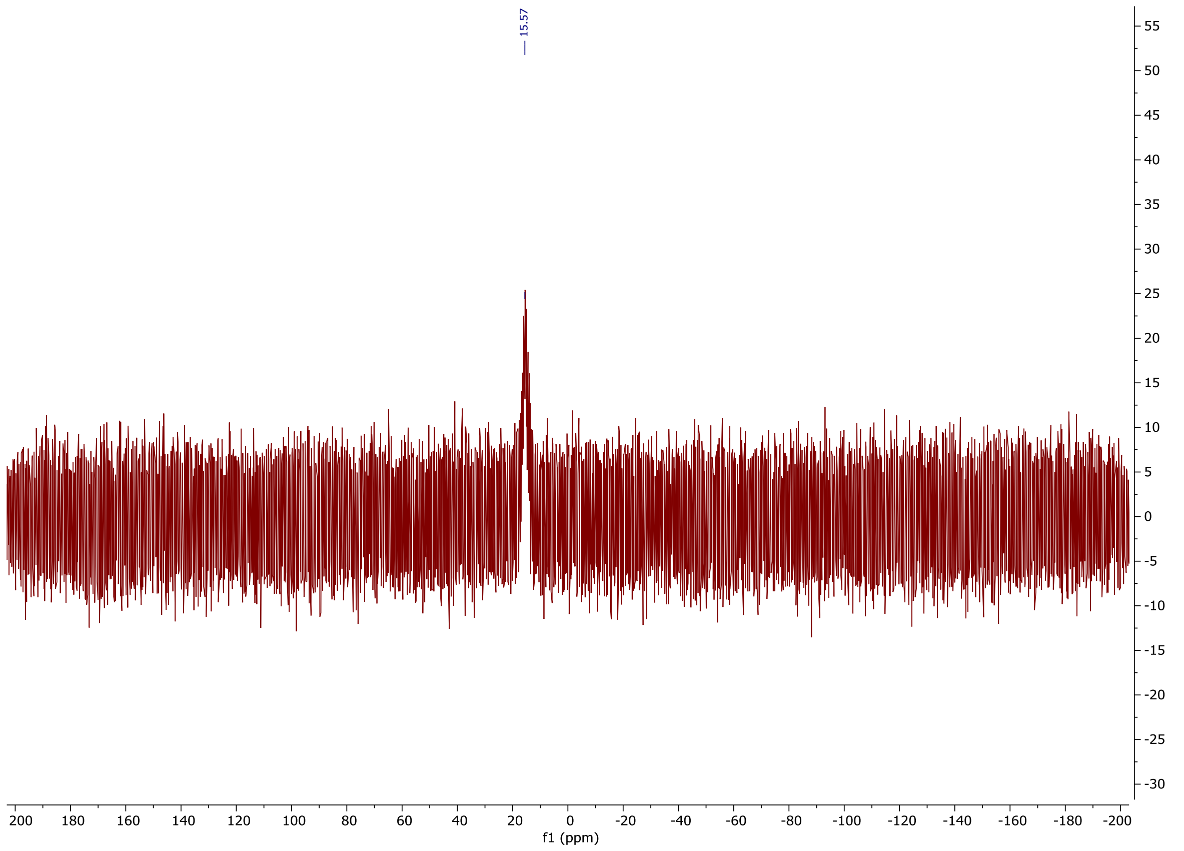

Figure S16: ^{31}P NMR (121 MHz, CD_3CN) of $[In(^{tBu}PN^{Et}N)Cl_2][In_2Cl_6]$

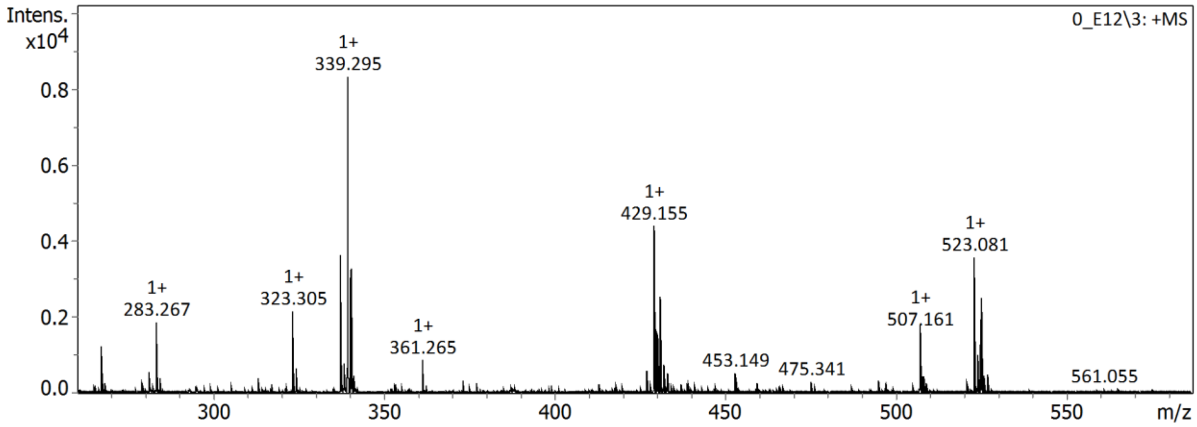

Figure S17: ESI-MS+ (m/z) of [In(^{Et}PN^{Et}N)Cl_2]In_2Cl_6 and its fragments

4. [In(^{R}NNN)Cl_2]$^+$

4.1 [2,6-Bis[1-(phenylimino)ethyl]pyridine] ligand

The ligand was synthesized according to modified literature procedure[2]
Aniline (1.14 mL, 12.5 mmol) was added to a solution of 2,6-diacetylpyridine (816 mg, 5 mmol) in methanol (20 mL). A few drops of formic acid were then introduced, and the reaction mixture was refluxed overnight. After completion, the volatile components were removed, and the residue was recrystallized from methanol at −4 °C. The resulting solid was washed with cold methanol to afford a yellow product in 55% yield.

^{1}H NMR (400 MHz, $CDCl_3$) δ 8.86 (t, *J* = 8.0 Hz, 1H), 8.67 (d, *J* = 7.9 Hz, 2H), 7.62 – 7.44 (m, 6H), 7.29 (s, 3H), 2.80 (d, *J* = 1.2 Hz, 6H); **^{13}C NMR** (101 MHz, $CDCl_3$) δ 18.42, 122.04, 128.82, 129.69, 130.07, 142.24, 144.62, 147.06, 167.51; **ESI-MS+** (m/z)- calculated for [$C_{21}H_{19}N_3$] 313.16, found 314.16 (ligandH$^+$); Yield = 55%

4.2 [In(^{Ph}NNN)Cl_2][$InCl_4$]

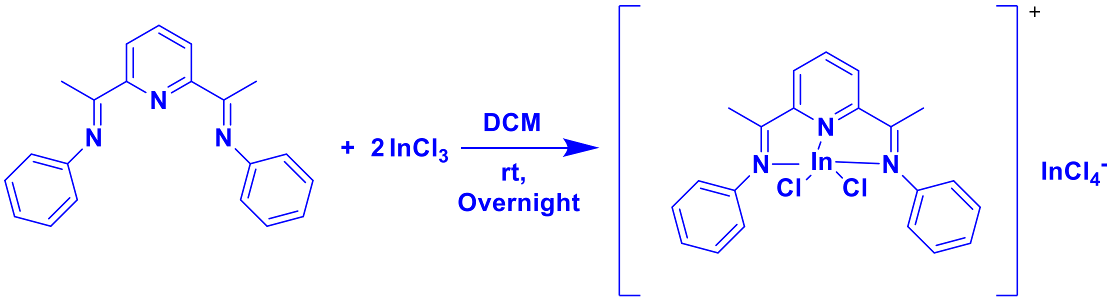

The ligand 2,6-Bis[1-(phenylimino)ethyl]pyridine (313 mg , 1 mmol) in DCM (10 ml) was treated with $InCl_3$ (442 mg, 2 mmol). The mixture was stirred overnight at room temperature, filtered to remove insolubles, and the filtrate was concentrated. The resulting solid was washed with pentane to afford the dark yellow product in 76% yield

^{1}H NMR (400 MHz, $CDCl_3$) δ 8.86 (t, *J* = 8.0 Hz, 1H), 8.67 (d, *J* = 7.9 Hz, 2H), 7.60 – 7.45 (m, 7H), 7.28 – 7.25 (m, 3H), 2.80 (d, *J* = 1.3 Hz, 7H);**^{13}C NMR** (101 MHz, $CDCl_3$) δ 18.48, 122.06, 128.82, 129.83, 130.07,

142.22, 144.62, 147.33, 167.74; **MS(MALDI-TOF)** = Calculated for $[C_{21}H_{19}Cl_2InN_3]^+$ 498.0, found 497.9; Yield = 76%

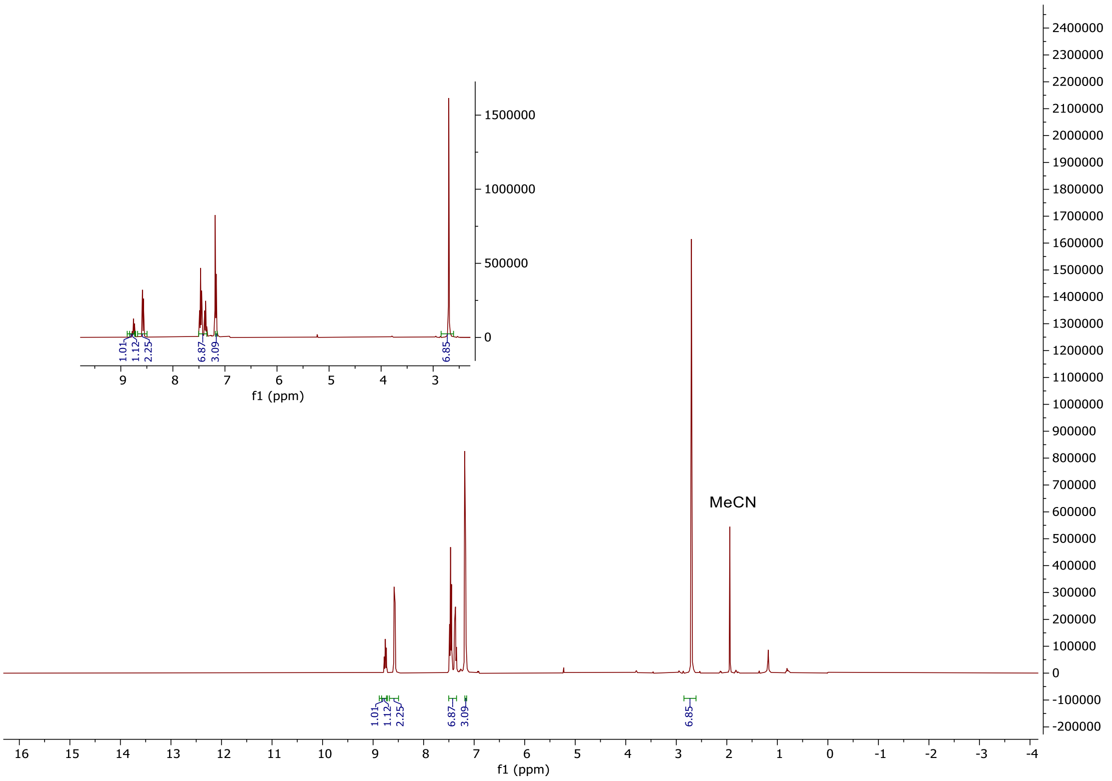

Figure S18: ^{1}H NMR (400 MHz, CD_3CN) of $[In(^{Ph}NNN)Cl_2][InCl_4]$

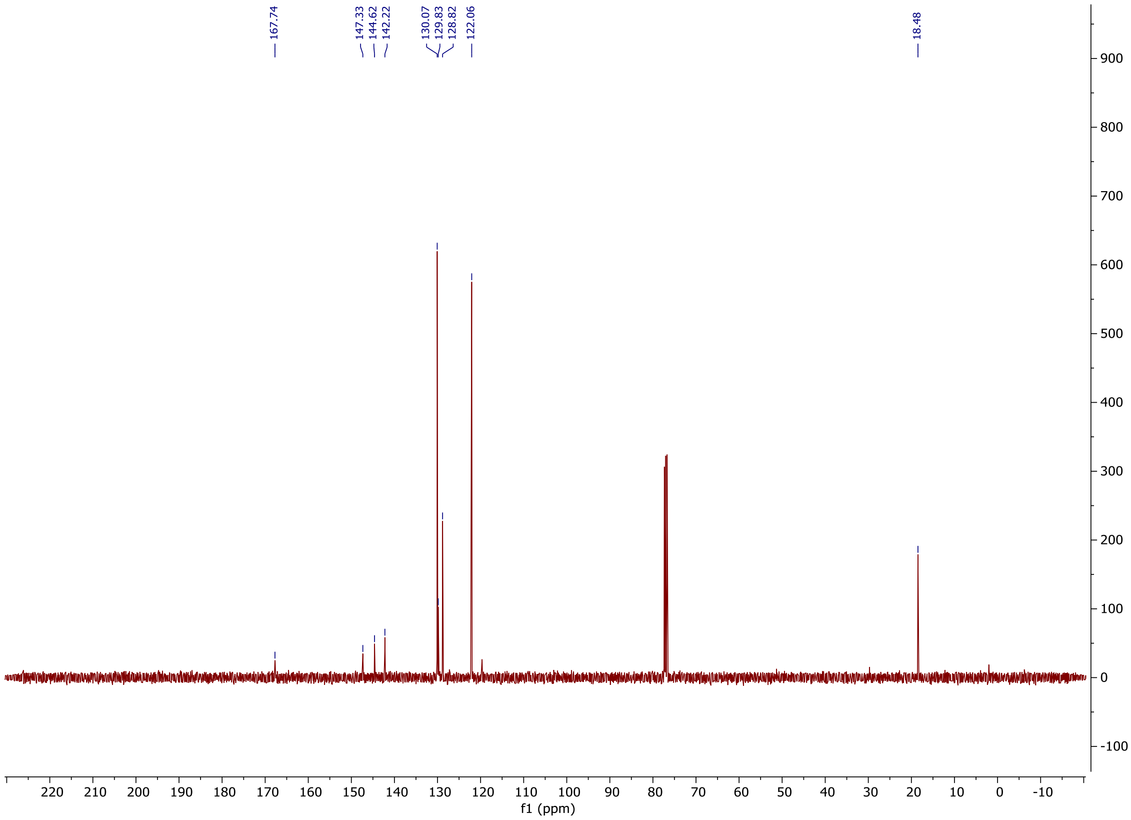

Figure S19: ^{13}C NMR (101 MHz, $CDCl_3$) of $[In(^{Ph}NNN)Cl_2][InCl_4]$

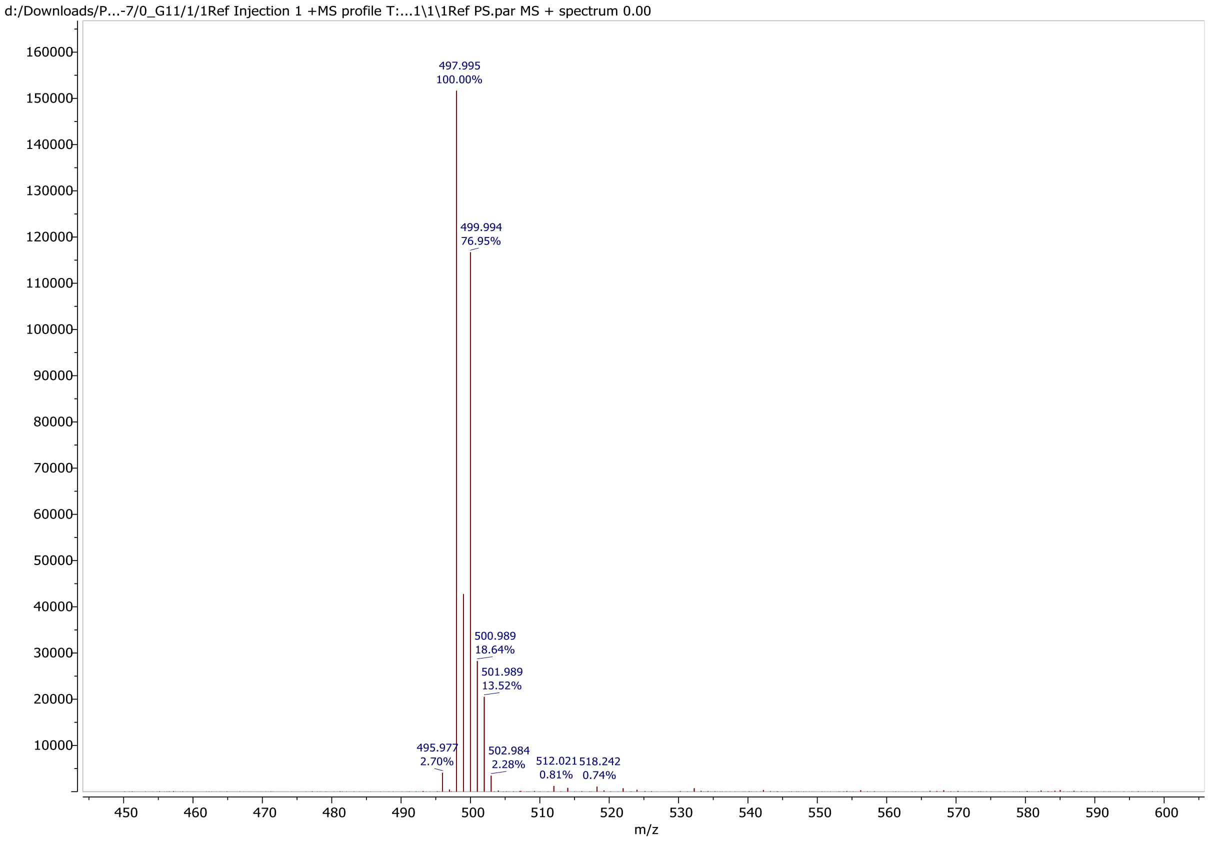

Figure S20: MS(MALDI-TOF) of [In(PhNNN)Cl_2][$InCl_4$]

4.3 [2,6-Bis[1-(2,6-diisopropylphenylimino)ethyl]pyridine] ligand

The ligand was synthesized according to modified literature procedure[2]

2,6-Diisopropylaniline (2.85 mL, 15 mmol) was added to a solution of 2,6-diacetylpyridine (1.16 g, 7.14 mmol) in absolute ethanol (20 mL). A few drops of glacial acetic acid were added, and the mixture was refluxed for overnight. Volatiles were removed under reduced pressure to yield a yellow solid.

^{1}H NMR (400 MHz, $CDCl_3$) δ 8.54 (d, *J* = 7.8 Hz, 2H), 7.97 (t, *J* = 7.8 Hz, 1H), 7.21 (d, *J* = 6.9 Hz, 4H), 7.17 – 7.12 (m, 2H), 2.81 (p, *J* = 7.0 Hz, 4H), 2.31 (s, 6H), 1.20 (d, *J* = 5.8 Hz, 24H); **^{13}C NMR** (101 MHz, $CDCl_3$) δ 17.29, 23.07, 23.38, 28.45, 76.84, 77.16, 77.48, 122.35, 123.15, 123.73, 135.93, 137.01, 146.64, 155.29, 167.07. **ESI-MS+** (m/z) = calculated for [$C_{33}H_{33}N_3$] 481.35, found 482.35 (ligandH^{+}) Yield=55%

4.4 [In(^{Dipp}NNN)Cl_2][$InCl_4$]

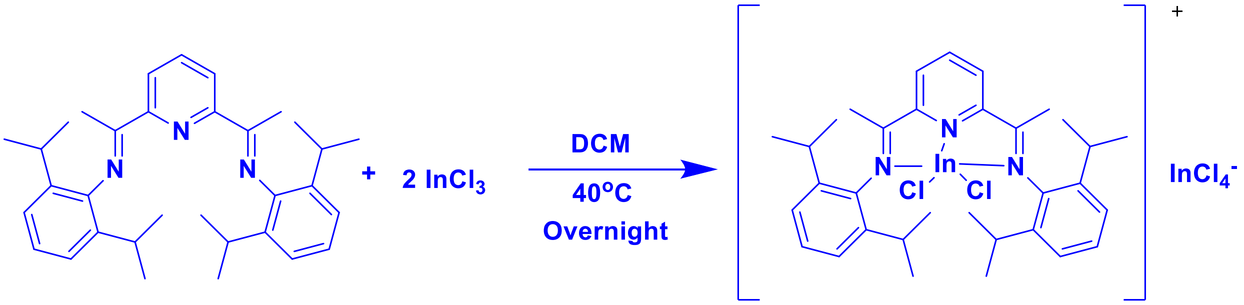

A solution of the ligand (480 mg, 1 mmol) in DCM (10 ml) was treated with $InCl_3$ (442 mg, 2 mmol) and stirred at 40 °C overnight. The solvent was then removed *in vacuo*, and the residue was washed with pentane to afford the yellow product in 72% yield.

^{1}H NMR (400 MHz, CD_3CN) δ 8.97 – 8.77 (m, 3H), 7.56 – 7.30 (m, 6H), 2.99 (h, *J* = 6.7 Hz, 4H), 2.70 (s, 6H), 1.20 (dd, *J* = 68.9, 6.7 Hz, 24H);**^{13}C NMR** (101 MHz, CD_3CN) δ 19.71, 23.68, 24.26, 28.28, 125.04, 128.68, 131.56, 138.31, 140.80, 143.54, 146.26, 171.59; **MS(MALDI-TOF)** = Calculated for $[C_{33}H_{43}Cl_2InN_3]^+$ 666.19, found 666.1; Yield=72%; **sc-XRD** see chapter 9.5

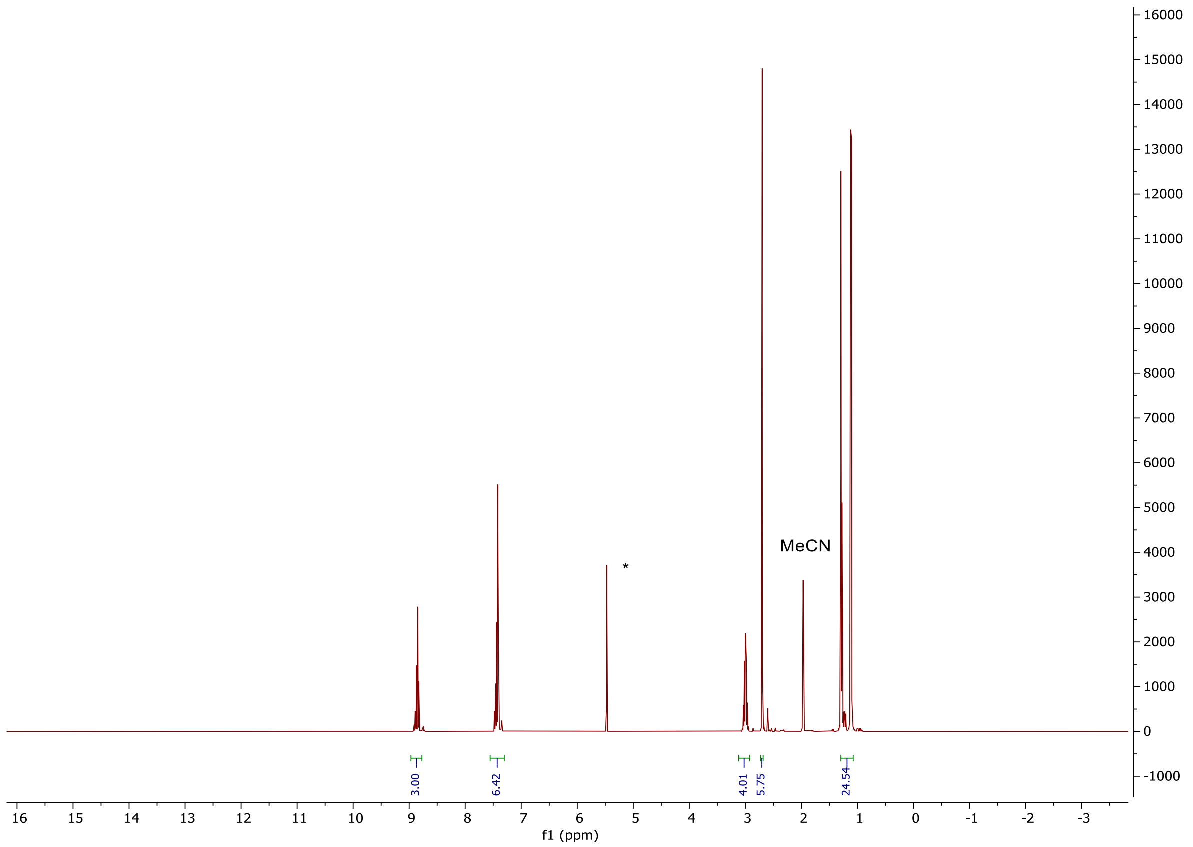

Figure S21: ^{1}H NMR (400 MHz, CD_3CN) of $[In(^{Dipp}NNN)Cl_2][InCl_4]$; * Impurity in MeCN-d3

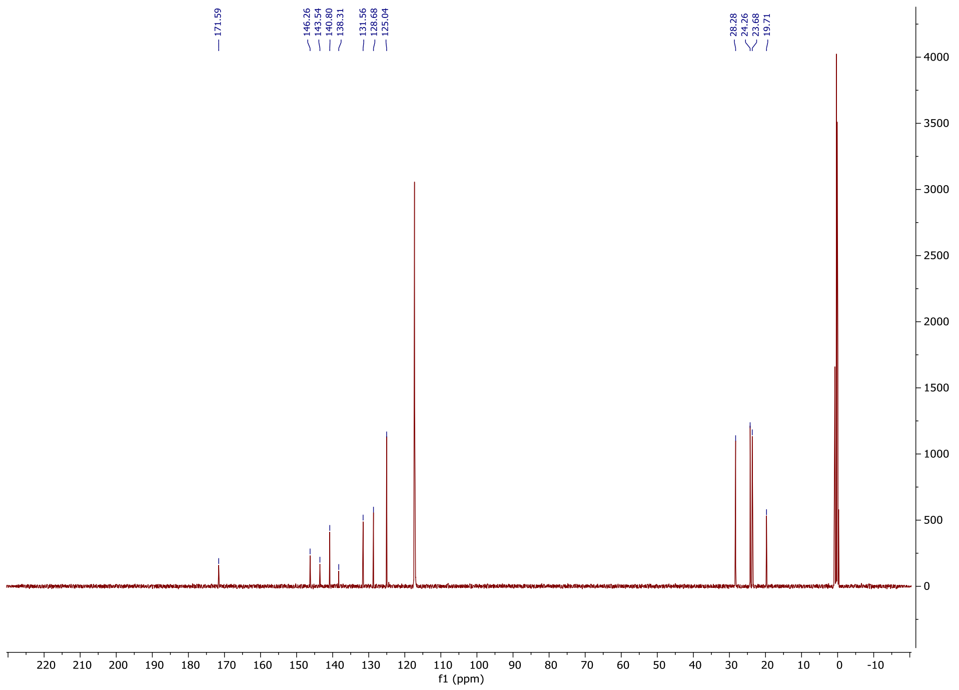

Figure S22: ^{13}C NMR (101 MHz, CD_3CN) of $[In(^{Dipp}NNN)Cl_2][InCl_4]$

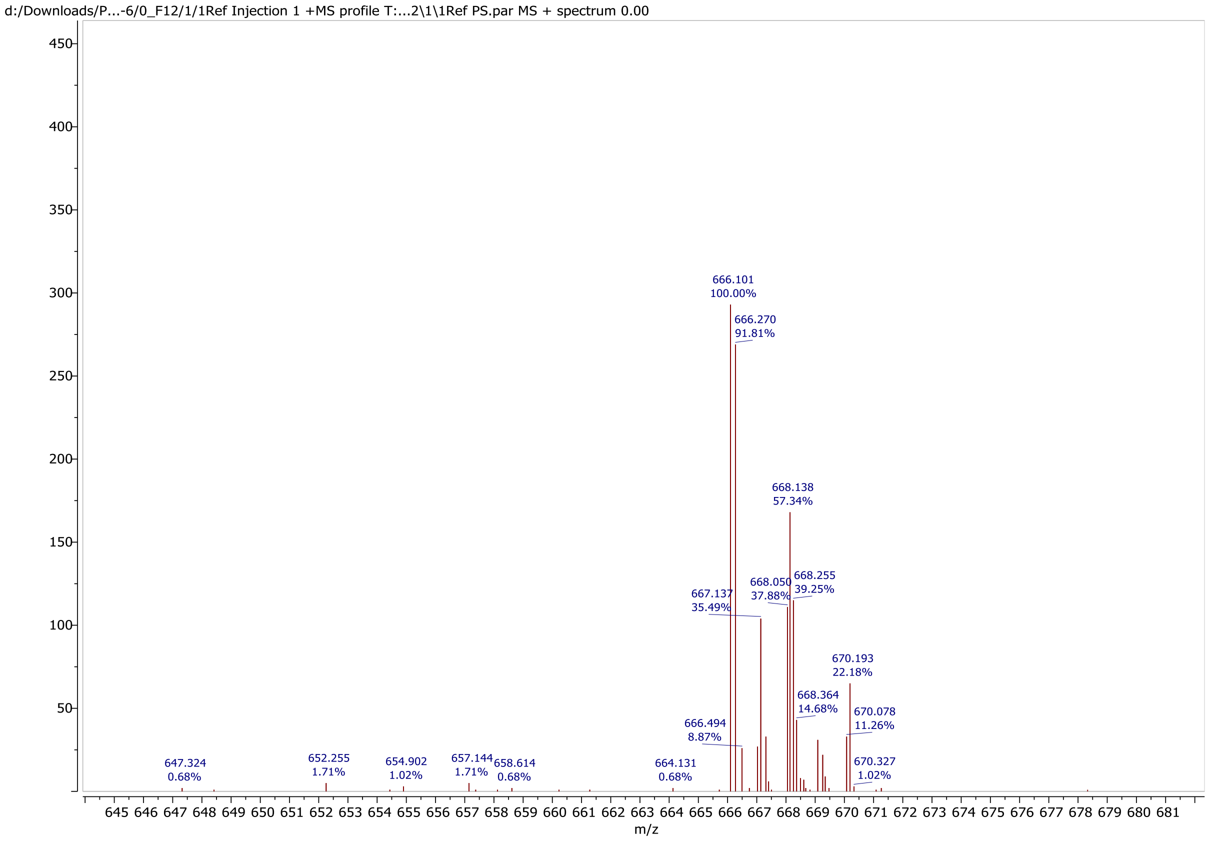

Figure S23: MS(MALDI-TOF) of $[In(^{Dipp}NNN)Cl_2][InCl_4]$

5. $[In(terpy)Br_2][B(C_6F_5)_4]$ and $[In(terpy)Br_2][CHB_{11}Cl_{11}]$

$[In(terpy)Br_3]$ was synthetized according to our published procedure.[1] Under inert atmosphere $[In(terpy)Br_3]$ (mg, mmol) in acetonitrile (mL) was treated with $Ag[B(C_6F_5)_4]$ (mg, mmol) or $Ag[CHB_{11}Cl_{11}]$ (mg, mmol) and stirred for in the dark for 3-days at room temperature. Precipitated AgBr was filtered, and the produced $[In(terpy)Br_2][B(C_6F_5)_4]$ or $[In(terpy)Br_2][CHB_{11}Cl_{11}]$ were obtained after evaporation of the reaction solvent.

$[In(terpy)Br_2][CHB_{11}Cl_{11}]$

^{1}H NMR (400 MHz, CD3CN) δ 9.04 (dt, *J* = 5.3, 1.2 Hz, 2H), 8.82 – 8.66 (m, 5H), 8.55 (td, *J* = 7.9, 1.6 Hz, 2H), 8.09 (ddd, *J* = 7.7, 5.3, 1.2 Hz, 2H), 4.10 (s, 1H); **^{13}C NMR** (101 MHz, CD3CN) δ 147.79, 145.06, 144.83, 144.43, 143.71, 129.05, 125.02, 124.83.**; ^{11}B{H} NMR** (128 MHz, CD3CN) δ -2.49, -11.59 (d, *J* = 383.4 Hz); **ESI-MS** (+) = Calculated for $[C_{15}H_9Br_2InN_3]$ + 507.83, found + 507.84. **ESI-MS** (-) = Calculated $[CHB_{11}Cl_{11}]$ + 521.77, found + 521.77 with the expected isotopic pattern and disctribution

$[In(terpy)Br_2][B(C_6F_5)_4]$

^{1}H NMR (400 MHz, CD3CN) δ 9.01 (d, *J* = 5.2 Hz, 2H), 8.85 – 8.61 (m, 5H), 8.54 (td, *J* = 7.9, 1.6 Hz, 2H), 8.17 – 8.03 (m, 2H); **^{13}C NMR** (101 MHz, CD3CN) δ 124.76, 124.92, 129.04, 143.62, 144.70,145.05, 148.04, 149.28; **^{19}F NMR** (376 MHz, CD3CN) δ -133.78,-163.94,-168.33**; ^{11}B NMR (128 MHz, CD3CN)** δ -16.70; **ESI-MS** (+) = Calculated for $[C_{15}H_9Br_2InN_3]$ + 507.83, found + 507.84.

6. GB-acidity measurement

The ^{31}P NMR experiments were performed under inert atmosphere in dry $CDCl_3$. A stock solution of Et_3PO (c = 0.13 M, 0.1 mL) was added to a solution of complex with each measurement. The measurements were repeated with increasing quantity of the measured complex until the ^{31}P signal of Et_3PO remained constant at the same chemical shift. The acceptor numbers (ANs) were calculated according to Equation 1. Experimental data and calculated values are displayed in Table 1

Equation 1:

$$AN = 2.21(\delta_{\text{sample}} - 41)$$

Table S1: ^{31}P NMR shifts and calculated values for Gutmann-Beckett acidity measurements

Catalyst	*Chemical shift (δ ppm)*	*AN*
$[In(^{tBu}PN^{Et}N)Cl_2]^+$	*54.5*	*30*
$[In(^{Cy}PNP)Cl_2]^+$	*53.5*	*28*
$[In(terpy)Cl_2]^+$ *(in CD3CN)*	*63.0*	*49*
$[Al(terpy)Cl_2]^+$ *(in CD3CN)*	*56.0*	*33*
$[Ga(terpy)Cl_2]^+$ *(in CD3CN)*	*59.0*	*40*
$[In(terpy)Br_2]^+$ *(in CD3CN)*	*64.0*	*51*
$[In(terpy)I_2]^+$ *(in CD3CN)*	*60.0*	*42*
$[In(^{Dipp}NNN)Cl_2]^+$	*70.0*	*64*

$[In(^{Ph}NNN)Cl_2]^+$	*70.1*	*64*

6.1 ^{31}P NMR shifts for GB-acidity measurements

$[In(^{Cy}PNP)Cl_2]^+$

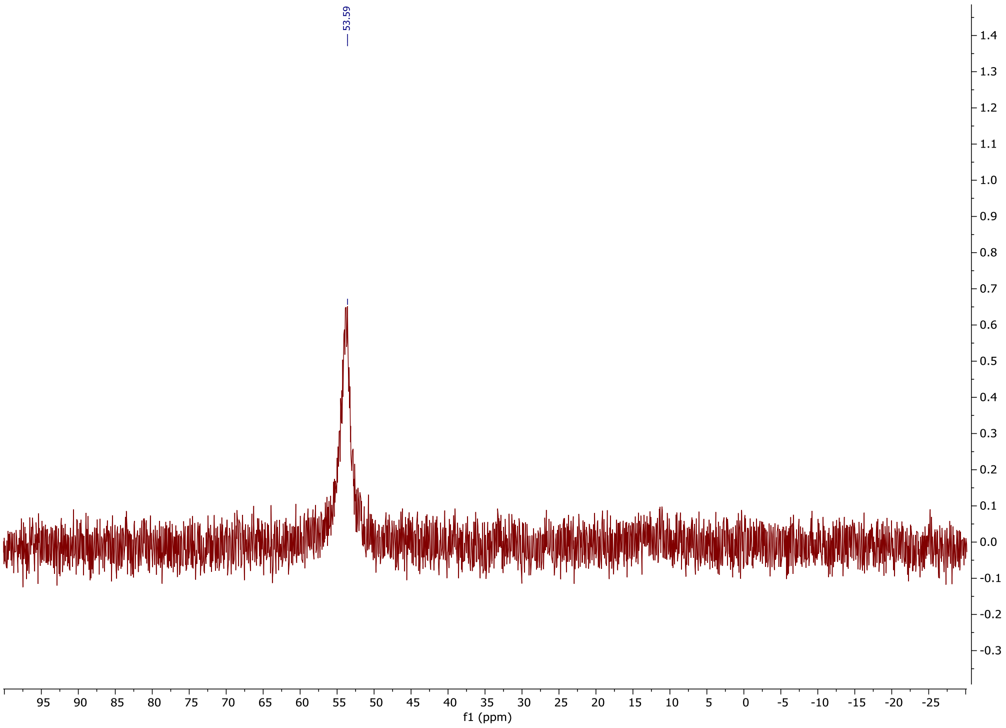

Figure S24: ^{31}P(121 MHz ,$CDCl_3$) spectra of $[In(^{Cy}PNP)Cl_2]^+$ after Et_3PO binding

$[In(terpy)Cl_2]^+$

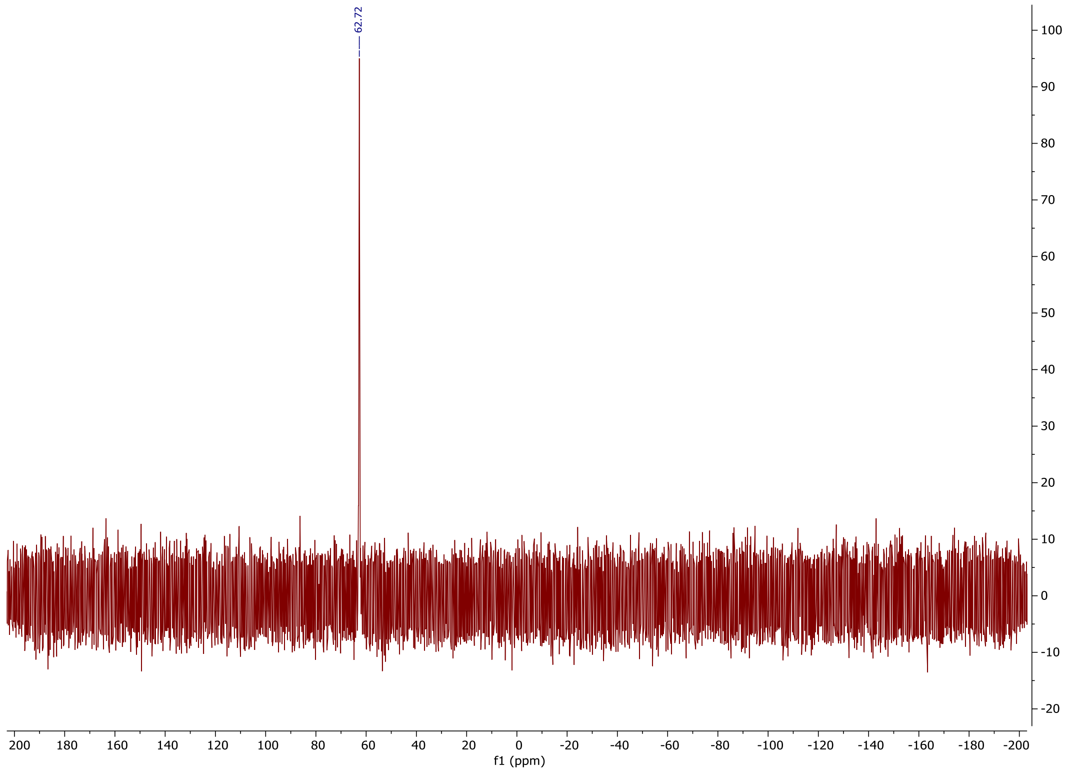

Figure S25 : ^{31}P(162 MHz, CD_3CN) spectra of $[In(terpy)Cl_2]^+$ after Et_3PO binding

$[Al(terpy)Cl_2]^+$

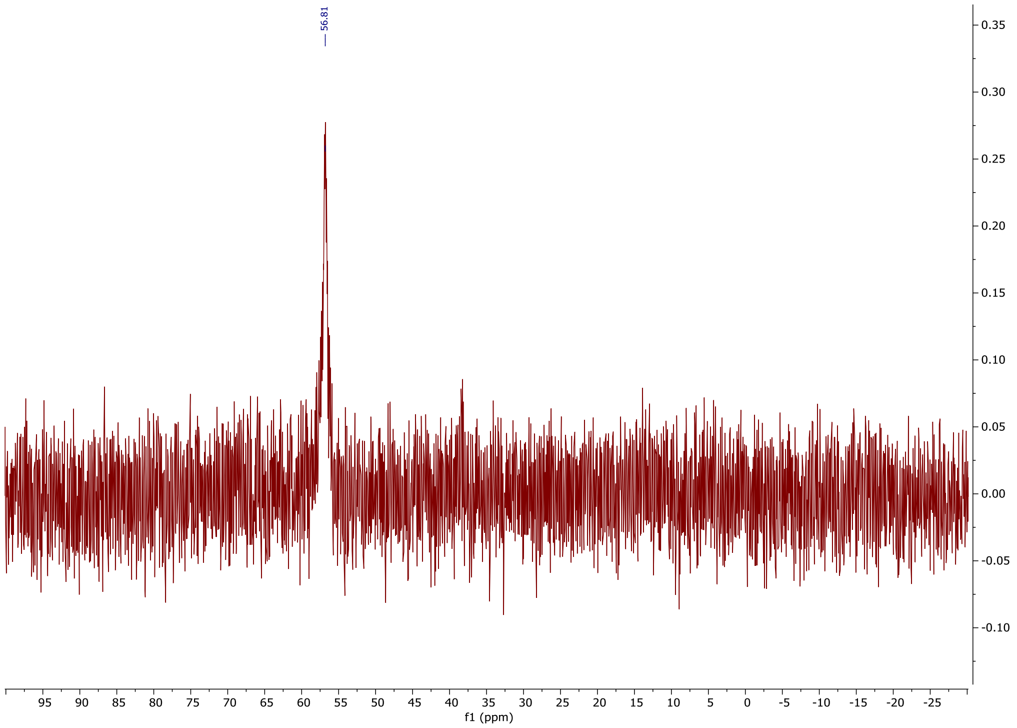

Figure S26 : ^{31}P(121 MHz,CD_3CN) spectra of $[Al(terpy)Cl_2]^+$ after Et_3PO binding

$[Ga(terpy)Cl_2]^+$

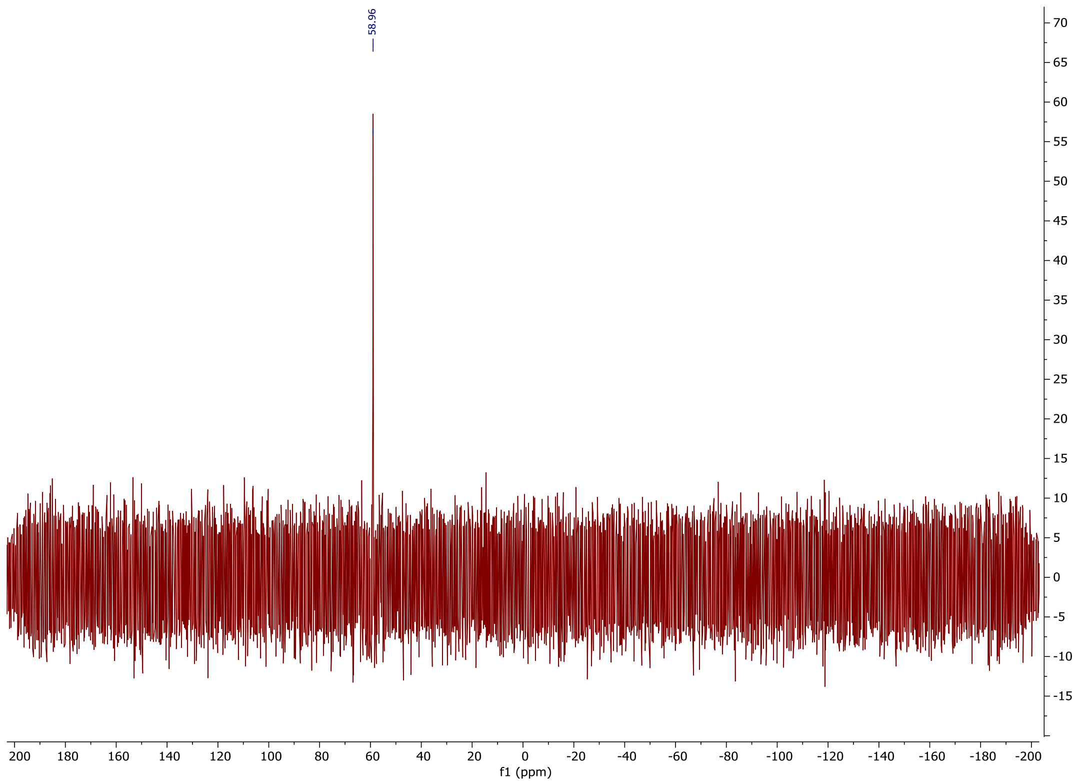

Figure S27 : ^{31}P(162 MHz CD_3CN) spectra of $[Ga(terpy)Cl_2]^+$ after Et_3PO binding

$[In(terpy)Br_2]^+$

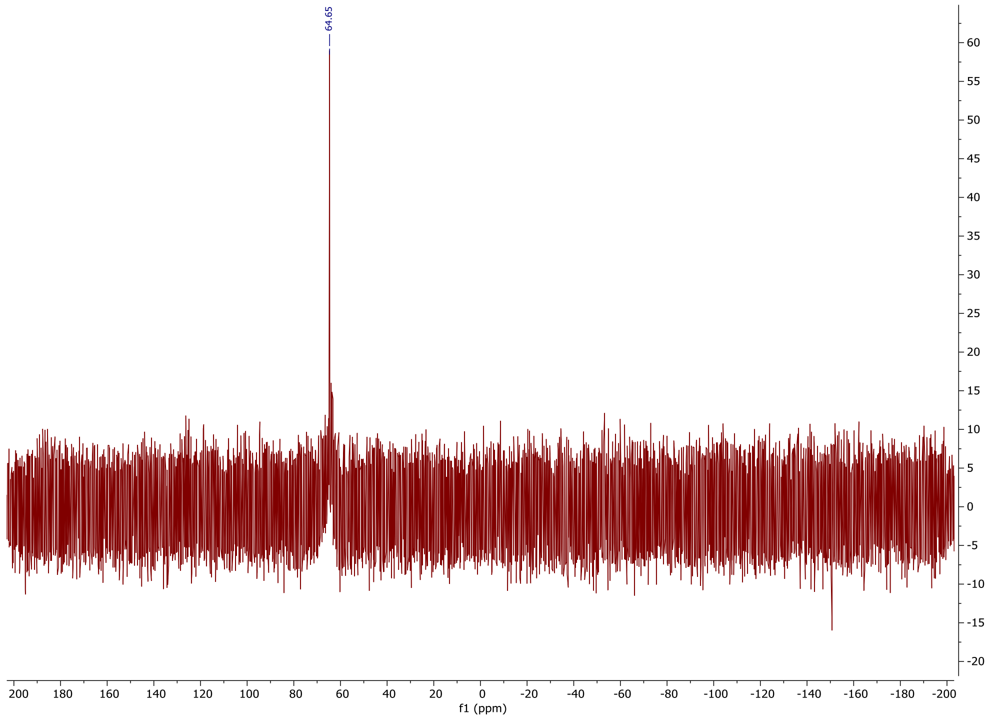

Figure S28 : ^{31}P(162 MHz,CD_3CN) spectra of $[In(terpy)Br_2]^+$ after Et_3PO binding

[In(terpy)I_2]$^+$

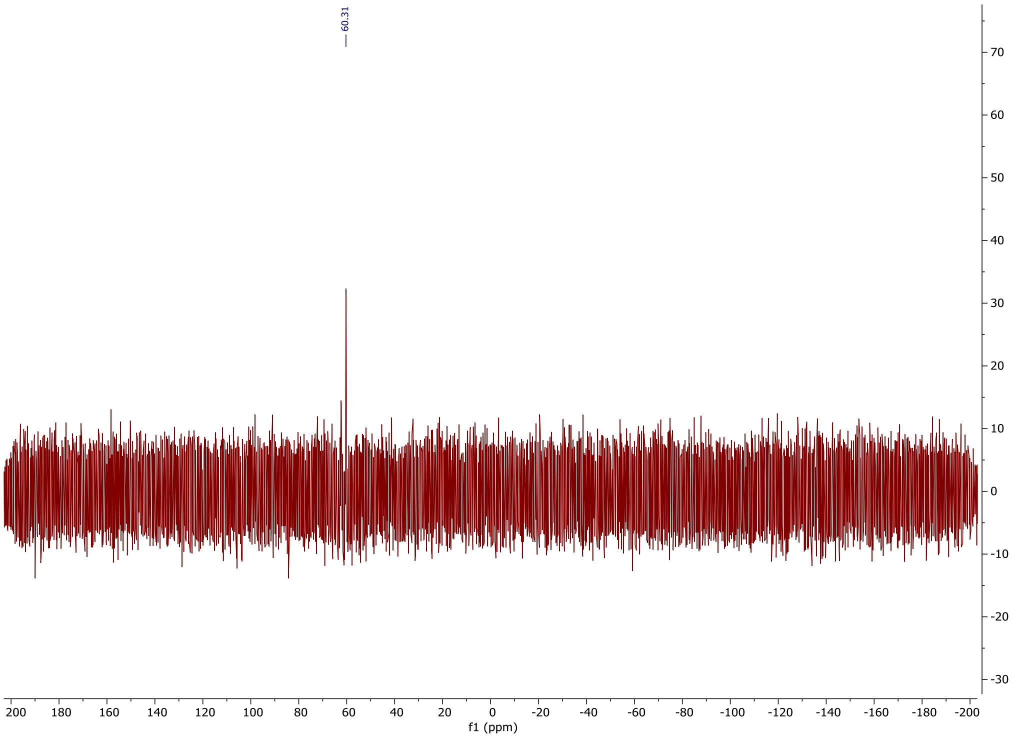

Figure S29 : ^{31}P(162 MHz,CD_3CN) spectra of [In(terpy)I_2]$^+$ after Et_3PO binding

[In(^{Dipp}NNN)Cl_2]$^+$

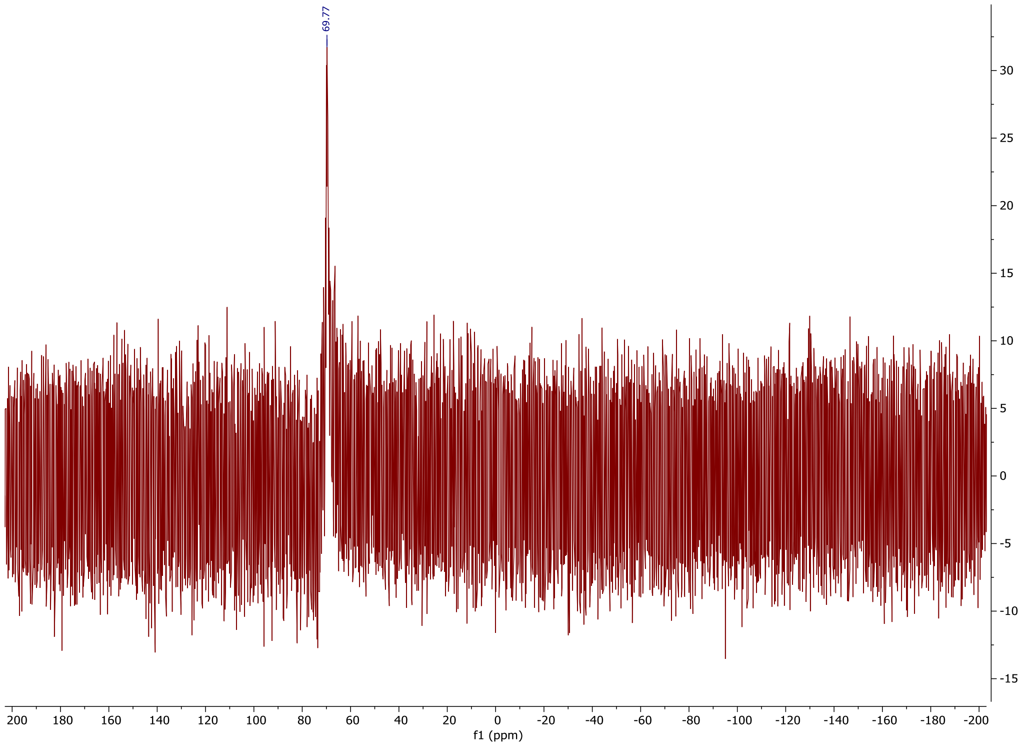

Figure S30 : ^{31}P(162 MHz,$CDCl_3$) spectra of [In(^{Dipp}NNN)Cl_2]$^+$ after Et_3PO binding

[In(^{Ph}NNN)Cl_2]$^+$

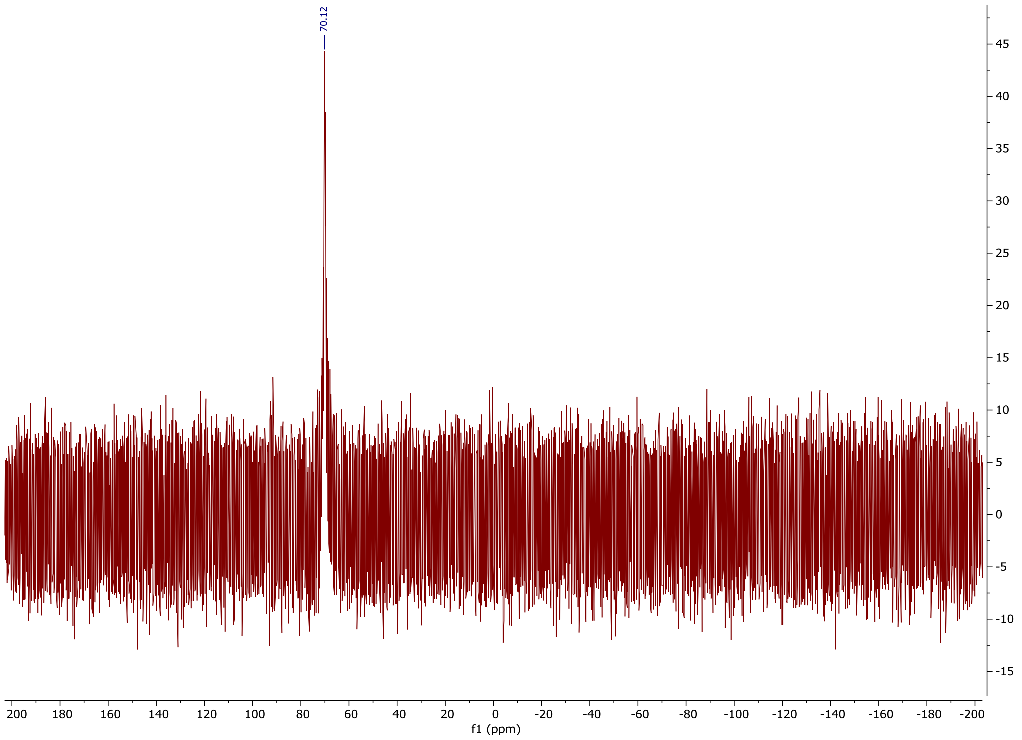

Figure S31 : ^{31}P(162 MHz,$CDCl_3$) spectra of [In(^{Ph}NNN)Cl_2]$^+$ after Et_3PO binding

7. Catalyst testing

Caution! *Hydrogen is classified as a GHS Flammable Gas, Category 1. Working with pressurized H_2 may lead to explosions. All reactions were performed in specialized high-pressure equipment certified for use with H_2 gas and within blast resistant enclosures.*

In a glove box, catalyst (0.10 mmol) and imine (1 mmol) were mixed in 1,3-DCB (4 mL) in a steel autoclave. The autoclave was then sealed and purged 3 times with 15 bar of H_2. The temperature and stirring rate were set using the Spec view program on Parr 5000 series multi reactor system. T = 0 was defined as the time the heating starts. The heating was turned off 1 hour before the end of the stated reaction time and allowed to cool down under pressure over the course of the remaining 1 hour of the test i.e., for a reaction time of 17 hours the heating was turned off after 16 hours and the reaction was depressurized after the 17- hour mark. Dibromomethane (1 mmol) was added to the reactor, stirred and an aliquot was taken for ^{1}H NMR analysis in $CDCl_3$. The conversion of imine and the yield of product were quantified by ^{1}H NMR analysis with the added dibromomethane as the internal standard. Other reaction products were quantified by their respective CH_2 signal in ^{1}H NMR and structures confirmed by ESI spectra.

7.1 General test result

Hydrogenation of the model substrate N-tert-butyl-1-phenylmethanimine to the product N-benzyl-2-methylpropan-2-amine:

^{1}H NMR (300 MHz, $cdcl_3$) δ 7.67 – 7.58 (m, 2H), 7.33 (s, 3H), 3.86 (s, 2H), 1.34 (s, 9H); **ESI(M+H):** expected 163.3 (M+H), found 162.0

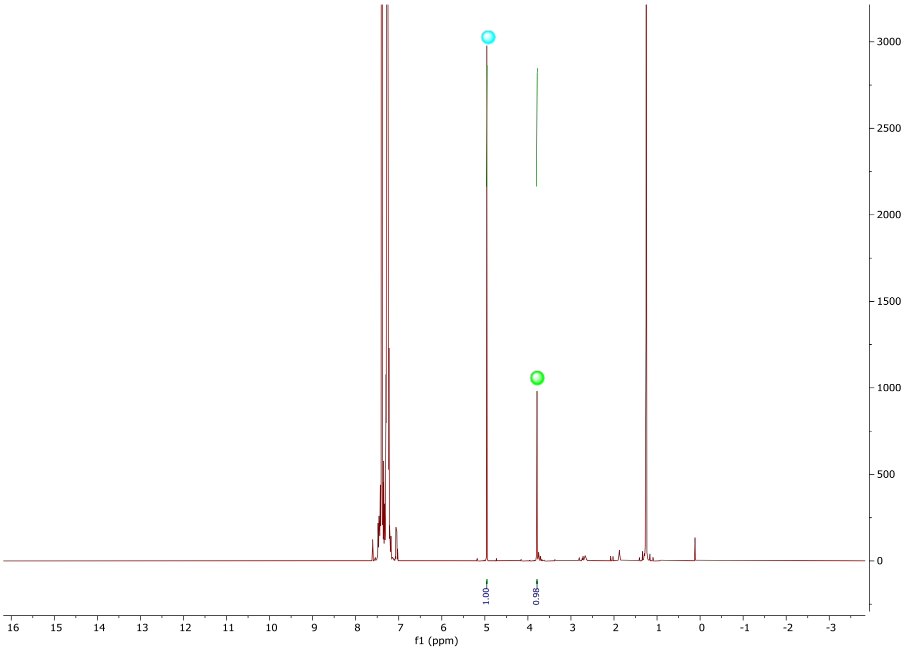

Figure S32: Crude ^{1}H NMR of catalytic test with model substrate *N-tert*-butyl-1-phenylmethanimine (-CH_2- marked green) and dibromomethane (blue) as internal standard

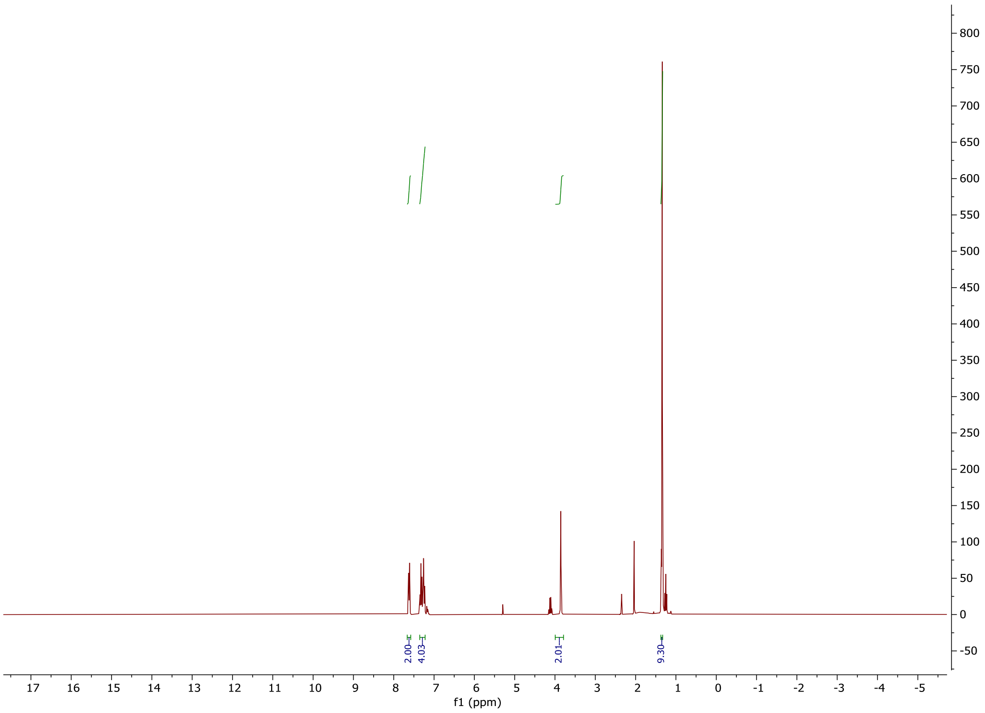

Figure S33: ^{1}H NMR (300 MHz, $CDCl_3$) of N-benzyl-2-methylpropan-2-amine extracted from the catalytic test

7.2 Effect of Lewis acid

Table S2: Catalyst optimization and reproducibility

Lewis acid	***Yield***
$[In(^{Et}P^{tBu}NN)Cl_2]^+$	48%,50%,52%
$[In(^{Cy}PNP)Cl_2]^+$	79%,80%,83%
$[In(terpy)Cl_2]^+$	84%,85%,85%
$[In(terpy)Br_2]^+$	96%,98%,99%
$[In(^{Ph}NNN)Cl_2]^+$	48%,50%,52%*
$[In(^{Dipp}NNN)Cl_2]^+$	39%,40%,40%*
$[In(terpy)Cl_2]^+$	*5%,5%,6%***
$[In(terpy)Br_2]^+$	*8%,10%,10%***
$[In(terpy)I_2]^+$	*20%,22%,25%***

*Reaction conditions: Imine (1 mmol), H_2 (15 Bar), 1,3-DCB (4 mL), Lewis acid (10 mol%), Pyrrolidine (20 mol%), temperature 120**°C** 17h.*Collidine instead of pyrrolidine, **T=100^{0}C*

7.3 H_2 pressure

Table S3: Optimization of reaction pressure with $[In(terpy)Cl_2]^+$

H_2 pressure(bar)	***Yield (%)***
5	9%, 10%, 10%
10	50%, 50%, 52%
15	84%, 85%, 85%
20	85%, 85%, 85%

*Reaction condition: Imine (1 mmol), $[In(terpy)Cl_2]^+$ (10mol%), 1,3-DCB (4 mL), Pyrrolidine(20 mol%), 120**°C** 17h*

7.4 Reaction temperature

Table S4: Optimization of reaction temperature with $[In(terpy)Cl_2]^+$

**Temperature**	**Yield**
160^{0}C	*75%,80%,80*^{a}
120^{0}C	84%,85%,85%
100^{0}C	*5%,5%,6%*

*Reaction conditions: Imine (1 mmol), $[In(terpy)Cl_2]^+$ (10mol%), 1,3-DCB (4 mL), pyrrolidine (20 mol%) , 17h**,***

7.5 Lewis base

Table S5: Optimization of Lewis base with $[In(terpy)Cl_2]^+$

Base	***Yield***
DABCO*	NR
TMPH	19%,20%,20%
DBU	5%,6%,7%
Quinuclidine	NR
pyrrolidine	84%,85%85%
2-methyl pyrrolidine	20%,22%,25%
1,2,2,6,6 pentamethylpiperidine	5%,6%,9%

*Reaction condition: Imine (1 mmole), $[In(terpy)Cl_2]^+$ (10 mol%), 1,3-DCB (4 mL), Lewis base (20mol%) , 120°C 17h, *T=160^{0}C*

7.6 Reaction solvent

Table S6: Optimization of reaction solvent

Solvent	***Yield***
Toluene	6%,7%,9%
1,*3*-DCB	84%,85%85%
DMF	NR
THF	NR

Reaction condition: Imine (1 mmole), $[In(terpy)Cl_2]^+$ (10 mol%), pyrrolidine (20mol%), solvent (4ml), 17h

8. H/D scrambling experiments

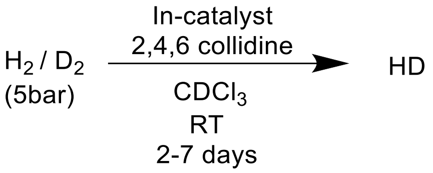

In a high-pressure NMR tube, the catalyst (5 mg) was dissolved in $CDCl_3$ and 2,4,6-collidine (3 µL) was added. The tube was pressurized with H_2/D_2 (5 bar) and left standing at room temperature for 2-7 days. HD gas was observed by ^1H NMR spectroscopy as a 1 : 1 : 1 triplet at 4.61 ppm with the corresponding protonated base peak at 14.4 ppm.

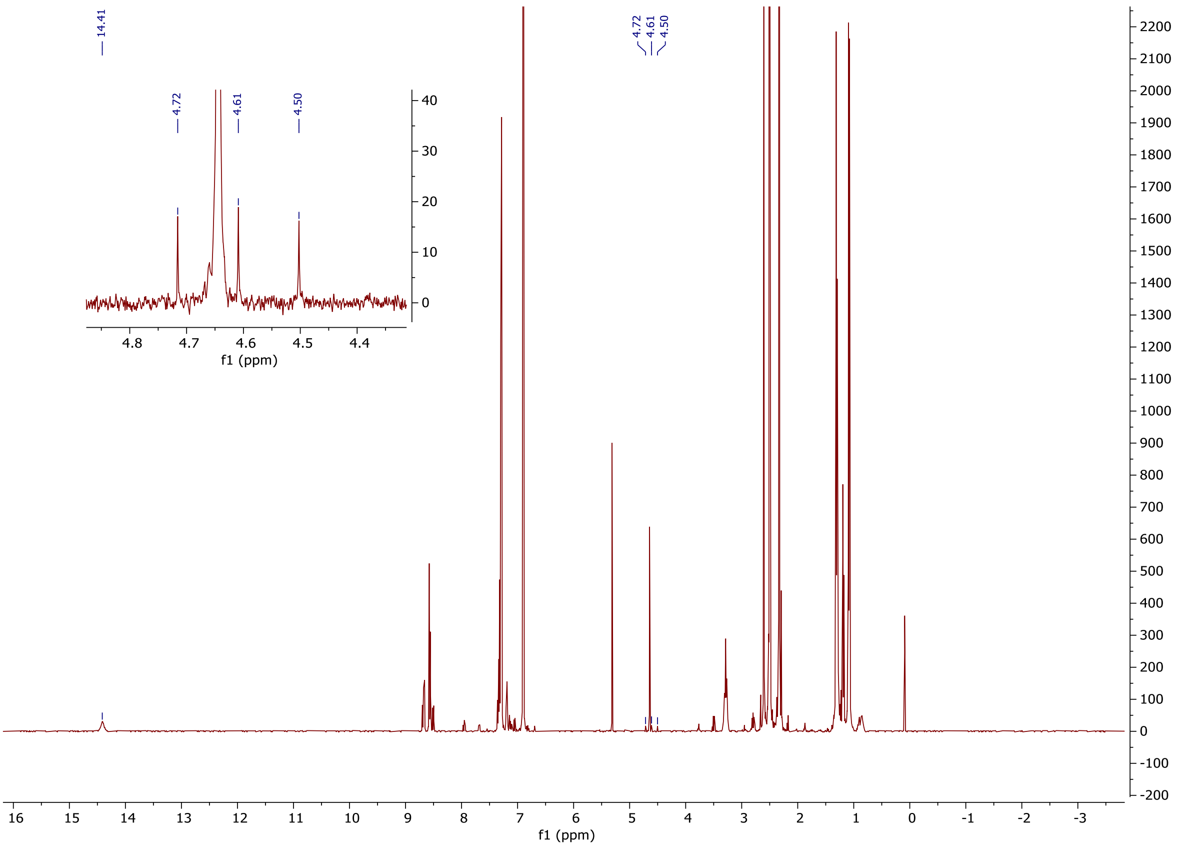

Figure S34: Example of ^1H NMR of H/D scrambling using $[In(^{Dipp}NNN)Cl_2]^+$ as the Lewis acid in $CDCl_3$

9. Crystallographic data

All crystallization experiments were conducted using mixtures of acetonitrile and heptane in a vapour diffusion setup. Single-crystal specimens selected from crystallization vessels under a polarization microscope were mounted on a Bruker D8 VENTURE Kappa Duo diffractometer equipped with a PHOTONIII detector, an Incoatec IμS microfocus sealed tube source and a Cryostream Cooler (Oxford Cryostreams). Diffraction data were collected using monochromated MoK_{α} radiation (λ = 0.71073 Å) at 120 K (**1**, **3** and **6**) or at 150 K (**2**, **4** and **5**). Primary data were processed using the diffractometer software.

The phase problem was solved by Intrinsic Phasing implemented in SHELXT-2018.[3]Structure models were refined using weighted full-matrix least-squares against F^2 (SHELXL-2019[4]). All non-hydrogen atoms were refined with anisotropic thermal displacement parameters; hydrogen atoms were refined isotropically. All carbon hydrogen atoms were refined using the riding model with U_{iso}(H) = 1.2 U_{eq}(C) for CH and CH_2 groups and with U_{iso}(H) = 1.5 U_{eq}(C) for CH_3 groups. The contribution of partial solvents that could not be refined satisfactorily in **4** and **6** were masked using the SQUEEZE procedure implemented in PLATON.[5]

. All geometric parameters and graphics were calculated and plotted using PLATON. Thermal ellipsoid plots are displayed in Figures S27 to S33. The crystallographic information files corresponding to structures can be accessed via Cambridge Crystallographic Data Centre at https://www.ccdc.cam.ac.uk/structures/ under deposition numbers CCDC-2432971, 2540606–2540610 and 2544995.

9.1 [In(terpy)Cl_2][$InCl_4$]

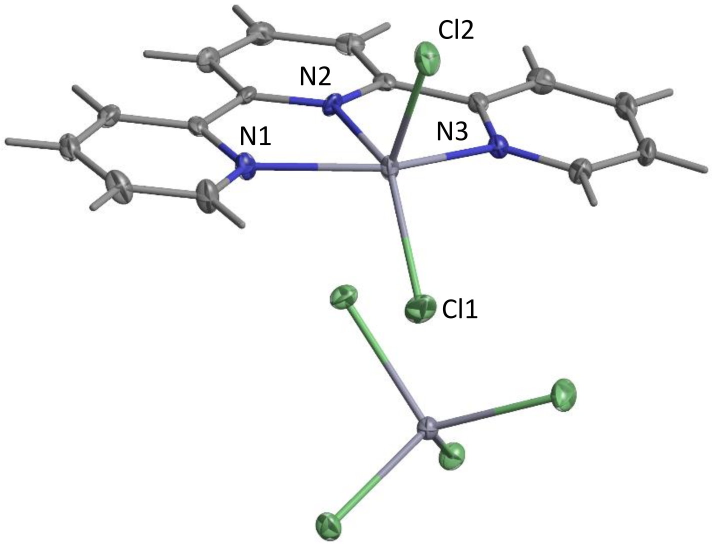

Figure S35: View of the asymmetric unit in the crystal structure [In(terpy)Cl_2][$InCl_4$] with the corresponding atom numbering scheme. Thermal displacement ellipsoids were plotted at the 50 % probability level. Selected bond lengths [Å] and valence angles [°]: In1-Cl1 2.333(1), In1-Cl2 2.350(1), In1-N1 2.254(3), In1-N2 2.222(4), In1-N3 2.239(4), Cl1-In1-Cl2 114.73(4), N1-In1-Cl1 100.36(9), N2-In1-Cl1 137.08(8), N3-In1-Cl1 100.0(1), N1-In1-Cl2 98.13(9), N2-In1-Cl2 108.19(8), N3-In1-Cl2 98.8(1). Applied colours: C – black, In – metallic, Cl – green, N – blue.

9.2 [In(terpy)Br_2][$InBr_4$]

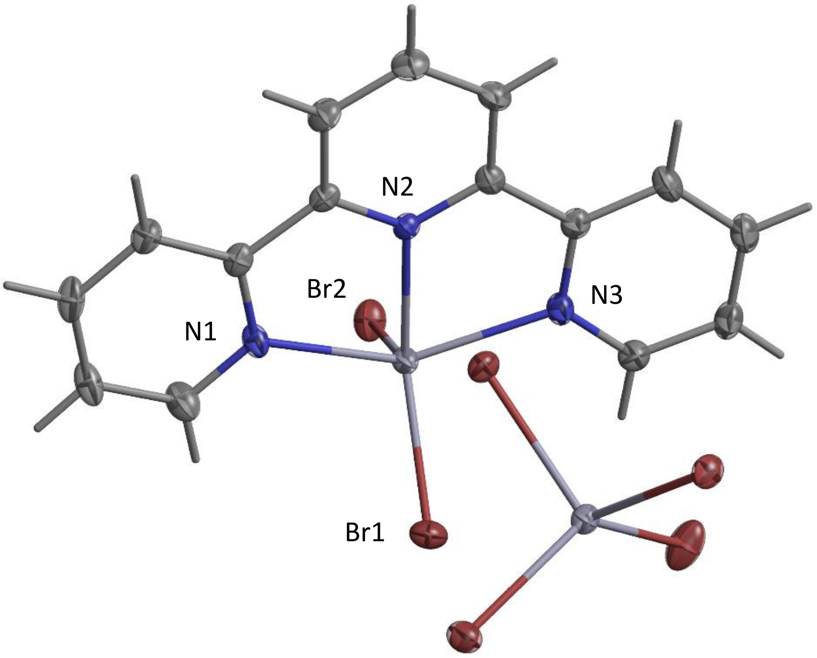

Figure S36: Solid-state structure of [In(terpy)Br_2][$InBr_4$] drawn with thermal ellipsoids on 50% probability level. The solvent dichloromethane was omitted for clarity. Selected bond lengths [Å] and valence angles [°]: In(1)-Br(1) 2.4642(5), In(1)-Br(2) 2.4737(5), In(1)-N(1) 2.262(3), In(1)-N(2) 2.224(3), In(1)-N(3) 2.256(3), Br(1)-In(1)-Br(2) 114.52(2), N(1)-In(1)-Br(1) 99.90(9), N(2)-In(1)-Br(1) 135.04(8), N(3)-In(1)-Br(1) 99.73(9), N(1)-In(1)-Br(2) 99.35(9), N(2)-In(1)-Br(2) 110.44(8), N(3)-In(1)-Br(2) 99.36(9) Colour legend: N – blue, Br – dark red, C – black, In – metallic

9.3 [In(terpy)I_2][InI_4]

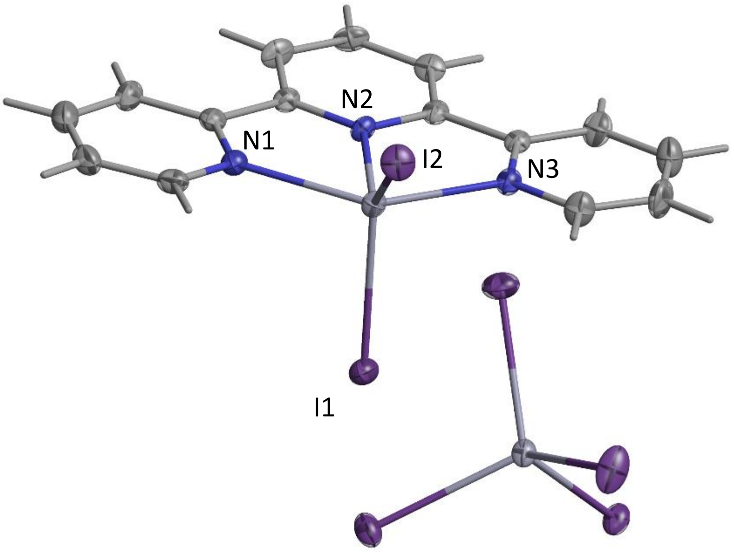

Figure S37: Solid-state structure of [In(terpy)I_2][InI_4] drawn with thermal ellipsoids on 50% probability level. Selected bond lengths [Å] and valence angles [°]: In(1)-N(2) 2.216(4), In(1)-N(3) 2.267(4), In(1)-

N(1) 2.273(4), In(1)-I(2) 2.6578(5), In(1)-I(1) 2.6878(6), N(2)-In(1)-N(3) 72.26(16), N(2)-In(1)-N(1) 71.89(16), N(3)-In(1)-N(1) 143.33(16), N(2)-In(1)-I(2) 133.99(11), N(3)-In(1)-I(2) 99.42(11), N(1)-In(1)-I(2) 99.78(11), N(2)-In(1)-I(1) 107.54(11), N(3)-In(1)-I(1) 96.71(11), N(1)-In(1)-I(1) 101.12(11), I(2)-In(1)-I(1) 118.432(19). Colour legend: N – blue, I – violet, C – grey, In – metallic

9.4 [In(^{Cy}PNP)Cl_2][$InCl_4$]

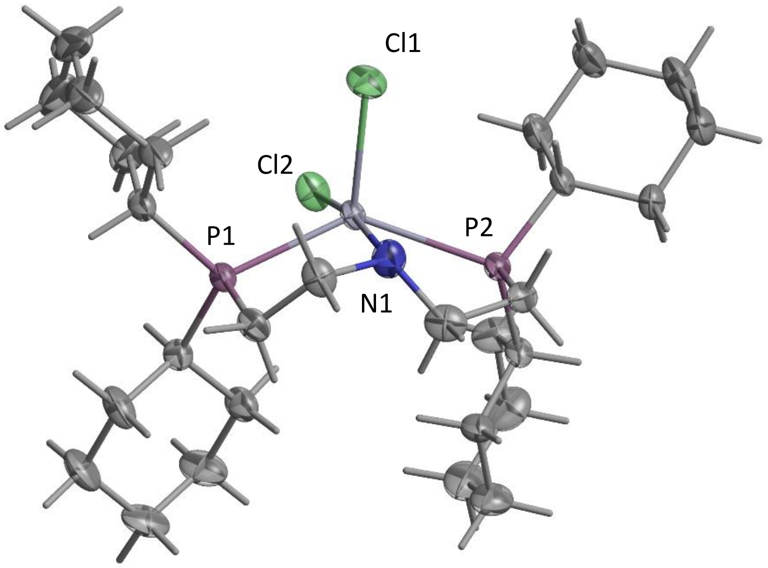

Figure S38: Solid-state structure of [In(^{Cy}PNP)Cl_2][$InCl_4$] drawn with thermal ellipsoids on 50% probability level. There are two symmetrically independent complex molecules located in the asymmetric part of the unit cell. For clarity, only a single molecule is displayed in this figure; the other complex molecule together with anions and solvates are omitted. Selected bond lengths [Å] and valence angles [°]: In(1)-Cl(1) 2.3933(13), In(1)-Cl(2) 2.4108(12), In(1)-N(1) 2.516(4), In(1)-P(2) 2.5549(12), In(1)-P(1) 2.5639(12), Cl(1)-In(1)-Cl(2) 102.50(5), Cl(1)-In(1)-N(1) 84.98(11), Cl(2)-In(1)-N(1) 172.27(11), Cl(1)-In(1)-P(2) 109.61(5), Cl(2)-In(1)-P(2) 98.53(4), N(1)-In(1)-P(2) 76.87(11), Cl(1)-In(1)-P(1) 104.64(4), Cl(2)-In(1)-P(1) 103.30(4), N(1)-In(1)-P(1) 76.30(11), P(2)-In(1)-P(1) 133.84(4).Colour legend: N – blue, P – plum, C – grey, In – metallic

9.5 $[In(^{Dipp}NNN)(MeCN)Cl_2][InCl_4]$

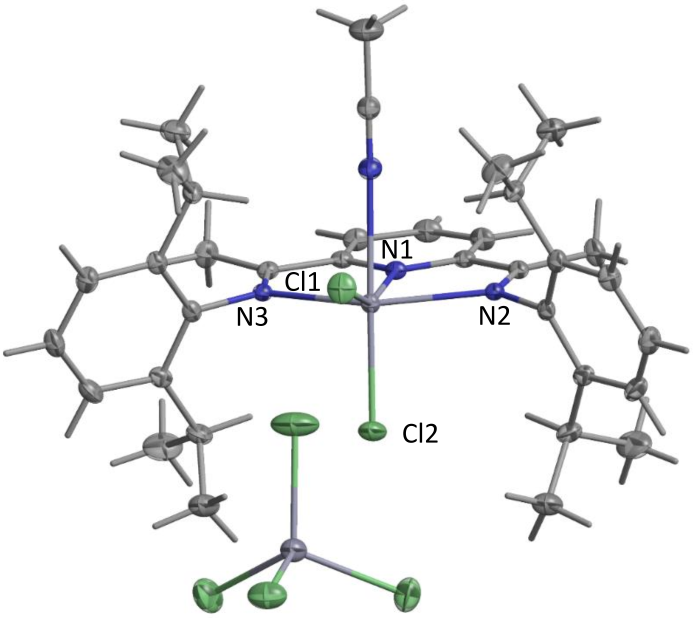

Figure S39: Solid-state structure of $[In(DippNNN)(MeCN)Cl_2][InCl_4]$ drawn with thermal ellipsoids on 50% probability level. The uncoordinated acetonitrile solvate is omitted for clarity. Selected bond lengths [Å] and valence angles [°]: In(1)-Cl(1) 2.3634(6), In(1)-Cl(2) 2.3701(7), In(1)-N(1) 2.2048(19), In(1)-N(2) 2.3202(14), In(1)-N(3) 2.3203(14), N(1)-In(1)-N(2) 72.03(3), N(1)-In(1)-N(3) 72.02(3), N(2)-In(1)-N(3) 144.04(7), N(1)-In(1)-Cl(1) 156.88(5), N(2)-In(1)-Cl(1) 106.33(3), N(3)-In(1)-Cl(1) 106.33(3), N(1)-In(1)-Cl(2) 102.92(5), N(2)-In(1)-Cl(2) 94.33(3), N(3)-In(1)-Cl(2) 94.33(3), Cl(1)-In(1)-Cl(2) 100.20(3). Colour legend: N – blue, P – plum, Cl – green, C – grey, In – metallic

9.6 $[In(^{tBu}PN^{Et}N)Cl_2][In_2Cl_6]$

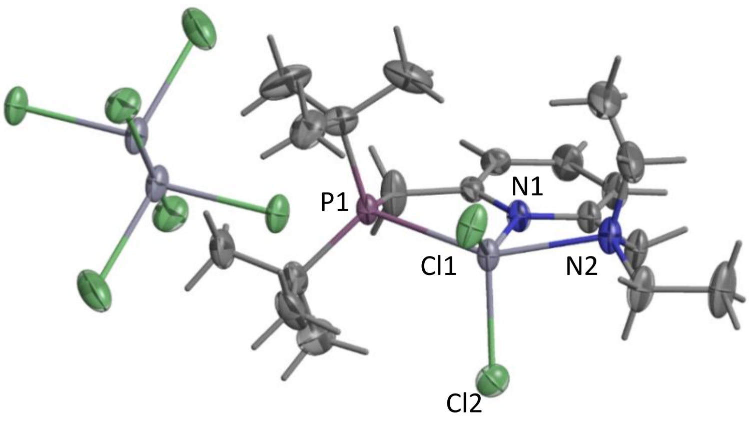

Figure S40: Solid-state structure of $[In(^{tBu}PN^{Et}N)Cl_2][In_2Cl_6]$ drawn with thermal ellipsoids on 50% probability level. Selected bond lengths [Å] and valence angles [°]: In(1)-N(1) 2.267(2), In(1)-N(2) 2.308(2), In(1)-Cl(2) 2.3755(8), In(1)-Cl(1) 2.3942(7), In(1)-P(1) 2.5973(7), N(1)-In(1)-N(2) 74.76(8),

N(1)-In(1)-Cl(2) 100.54(6), N(2)-In(1)-Cl(2) 97.38(6), N(1)-In(1)-Cl(1) 147.34(6), N(2)-In(1)-Cl(1) 93.11(6), Cl(2)-In(1)-Cl(1) 111.17(3), N(1)-In(1)-P(1) 77.51(5), N(2)-In(1)-P(1) 145.39(6), Cl(2)-In(1)-P(1) 107.76(2), Cl(1)-In(1)-P(1) 99.31(2).Colour legend: N – blue, P – plum, Cl – green, C – grey, In – metallic

9.7 [In(^{Cy}PNP)Cl_3]

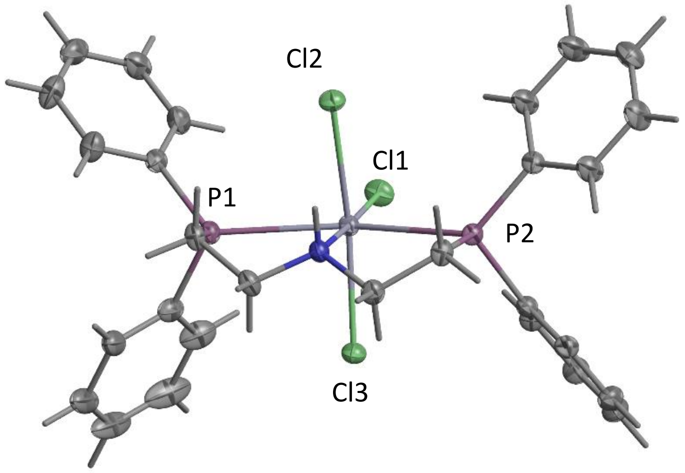

Figure S41: Solid-state structure of [In(^{Cy}PNP)Cl_3] drawn with thermal ellipsoids on 50% probability level. Selected bond lengths [Å] and valence angles [°]: In(1)-N(1) 2.391(2), In(1)-Cl(1) 2.4332(7), In(1)-Cl(2) 2.4690(6), In(1)-Cl(3) 2.5163(6), In(1)-P(2) 2.6719(7), In(1)-P(1) 2.7050(7), N(1)-In(1)-Cl(1) 177.05(5), N(1)-In(1)-Cl(2) 85.35(5), Cl(1)-In(1)-Cl(2) 97.58(2), N(1)-In(1)-Cl(3) 84.21(5), Cl(1)-In(1)-Cl(3) 92.87(2), Cl(2)-In(1)-Cl(3) 169.22(2), N(1)-In(1)-P(2) 76.91(5), Cl(1)-In(1)-P(2) 103.39(2), Cl(2)-In(1)-P(2) 89.21(2), Cl(3)-In(1)-P(2) 85.81(2), N(1)-In(1)-P(1) 75.23(5), Cl(1)-In(1)-P(1) 104.47(2), Cl(2)-In(1)-P(1) 87.71(2), Cl(3)-In(1)-P(1) 92.23(2), P(2)-In(1)-P(1) 152.13(2). Colour legend: N – blue, P – plum, Cl – green, C – grey, In – metallic

10. Computational details

10.1 HIA and FIA calculations

Hydride ion affinity (HIA) and fluoride ion affinity (FIA) values were computed following the method validated by Greb and co-workers[6,7] employing the calculated HIA and FIA values for the TMS cation for isodesmic anchoring. Geometry optimizations of all Lewis acids (LAs) and their corresponding hydride and fluoride adducts ([LA–H] and [LA–F]) in the corresponding *cis* or *trans* configurations were performed using the PBEh-3c/def2-mSVP composite method as implemented in ORCA 6.1[8]. In all cases, the optimized structures were confirmed to be local minima by vibrational frequency analysis. Single point energy calculations were then performed at the DSD-PBEP86/Def2QZVP level, including D3BJ damping. The ion affinities were calculated from the change in enthalpy of the following reactions:

$$[LA]^+ + TMS\text{-}H \rightarrow [TMS]^+ + LA\text{-}H$$

$$[LA]^+ + TMS\text{-}F \rightarrow [TMS]^+ + LA\text{-}F$$

Table S7. Gas-phase enthalpies computed at the DSD-PBEP86/Def2QZVP//PBEh-3c/def2-mSVP level of theory (in hartrees)

	LA	LA-H *cis*	LA-H *trans*	LA-F *cis*	LA-F *trans*
$[In(terpy)Cl_2]^+$	-1850.923010	-1851.665341	-1851.674036	-1950.913587	-1950.911856
$[Ga(terpy)Cl_2]^+$	-3585.191163	-3585.920652	-3585.929542	-3685.180055	-3685.180123
$[Al(terpy)Cl_2]^+$	-1903.326187	-1904.051154	-1904.055227	-2003.334621	-2003.338443
$[In(terpy)Br_2]^+$	-6077.851041	-6078.591849	-6078.601650	-6177.840698	-6177.838508
$[In(terpy)I_2]^+$	-1526.033342	-1526.771428	-1526.781356	-1626.020831	-1626.017621
$[In(^{Dipp}NNN)Cl_2]^+$	-2554.661464	-2555.405797	-2555.414321	-2654.656316	-2654.648125
$[In(^{Ph}NNN)Cl_2]^+$	-2083.903768	-2084.647327	-2084.659483	-2183.894473	-2183.893348
$[In(^{tBu}PN^{EtN})Cl_2]^+$	-2303.667304	-2304.405447	-2304.405476	-2403.657940	-2403.645234
$[In(^{Cy}PNP)Cl_2]^+$	-2943.153390	-2943.884729	-2943.896817	-3043.143715	-3043.122952

Table S8. HIA and FIA values computed at the DSD-PBEP86/Def2QZVP//PBEh-3c/def2-mSVP level of theory (in kJ/mol)

	HIA *cis*	HIA *trans*	FIA *cis*	FIA *trans*
$[In(terpy)Cl_2]^+$	579.8	602.7	568.7	564.2
$[Ga(terpy)Cl_2]^+$	546.1	569.4	564.3	564.5
$[Al(terpy)Cl_2]^+$	534.2	544.9	615.6	626.6
$[In(terpy)Br_2]^+$	575.8	601.6	566.3	560.5
$[In(terpy)I_2]^+$	568.7	594.7	560.6	552.2
$[In(^{Dipp}NNN)Cl_2]^+$	585.1	607.5	579.9	588.4
$[In(^{Ph}NNN)Cl_2]^+$	583.0	615.0	569.0	566.1
$[In(^{tBu}PN^{Et}N)Cl_2]^+$	568.8	568.9	568.9	535.5
$[In(^{Cy}PNP)Cl_2]^+$	551.0	582.7	568.0	513.5

10.2 Energy profiles for LA-hydride formation

For thermochemistry calculations the Gaussian 16 package was employed[9]. The M06-2X method [10] was used together with the def2-SVP basis set for optimization of the relevant structures, and then reoptimizations at the M06-2X/def2-TZVP level of theory were performed to confirm the absence of imaginary frequencies for minima and to obtain more precise Gibbs free energies. Solvation was included with the self-consistent reaction field (SCRF) approach, employing the IEFPCM model [11] with toluene parameters. For visualization and graphic rendering, VMD 1.9.3 [12] and CYLView20 [13] programs were used. The cartesian coordinates of relevant geometries are available in a supporting .zip file.

The Gibbs free energies of the isolated lewis acids (LA), their adducts with pyrrolidine in either the *cis* or *trans* configurations, and the corresponding *cis* and *trans* hydrides are shown in Table SX. In order to interpret the energy profiles, relative energies with respect to the isolated reactants (LA + pyrrolidine + H_2) were calculated and gathered in Table SX2. The most stable LA-(pyr) configurations are highlighted in yellow, while the most stable hydrides are shaded in blue.

Table S9. Gibbs free energies computed at the M06-2X-D3/def2-TZVP//IEFPCM (toluene) level of theory (in hartrees)

	LA	LA-(pyr) *cis*	LA-(pyr) *trans*	LA-H *cis*	LA-H *trans*
$[In(terpy)Cl_2]^+$	-1852,762359	-2065,233844	-2065,229287	-1853,483885	-1853,492789
$[Ga(terpy)Cl_2]^+$	-3587,566365	-3800,029585	-3799,993441	-3588,27341	-3588,28167
$[Al(terpy)Cl_2]^+$	-1905,190067	-2117,658543	-2117,650434	-1905,891789	-1905,89612
$[In(terpy)Br_2]^+$	-6080,763136	-6293,232048	-6293,230322	-6081,485476	-6081,494843
$[In(terpy)I_2]^+$	-1527,638145	-1740,10437	-1740,100168	-1528,358697	-1528,368352
$[In(^{Dipp}NNN)Cl_2]^+$	-2557,445041	-2769,888996	-2769,923811	-2558,172983	-2558,179327
$[In(^{Ph}NNN)Cl_2]^+$	-2086,062375	-2298,528937	-2298,545417	-2086,785412	-2086,798049
$[In(^{tBu}PN^{Et}N)Cl_2]^+$	-2305,910723	-2518,343815	-2518,360995	-2306,629202	-2306,626658
$[In(^{Cy}PNP)Cl_2]^+$	-2946,055717	-3158,501818	-3158,513461	-2946,774347	-2946,768838
pyrrolidine	-212,458204				
pyrrolidine-H^+	-212,862668				
H_2	-1,14506				

Table S10. Relative Gibbs free energies computed with respect to isolated reactants in kcal/mol

	LA-(pyr) *cis* + H_2	LA-(pyr) *trans* + H_2	LA-H *cis* + pyr-H^+	LA-H *trans* + pyr-H^+
$[In(terpy)Cl_2]^+$	-8,3	-5,5	12,0	6,4
$[Ga(terpy)Cl_2]^+$	-3,1	19,5	21,1	15,9
$[Al(terpy)Cl_2]^+$	-6,4	-1,4	24,4	21,7
$[In(terpy)Br_2]^+$	-6,7	-5,6	11,5	5,6
$[In(terpy)I_2]^+$	-5,0	-2,4	12,6	6,5
$[In(^{Dipp}NNN)Cl_2]^+$	8,9	-12,9	7,9	4,0
$[In(^{Ph}NNN)Cl_2]^+$	-5,2	-15,6	11,0	3,1

[In(^{tBu}PN^{Et}N)Cl_2]$^+$	15,8	5,0	13,9	15,5
[In(^{Cy}PNP)Cl_2]$^+$	7,6	0,3	13,8	17,2

10.4 Relevant Molecular Orbitals

The relevant unoccupied MOs for representative pincer catalysts are depicted in Figure S34. In each case, the virtual orbitals with significant contribution over the indium atom were identified. In all cases, both Mulliken and Ros-Schuit analyses pointed out that LUMO orbital exhibits *s* symmetry, while LUMO+n presents dominant *p* character.

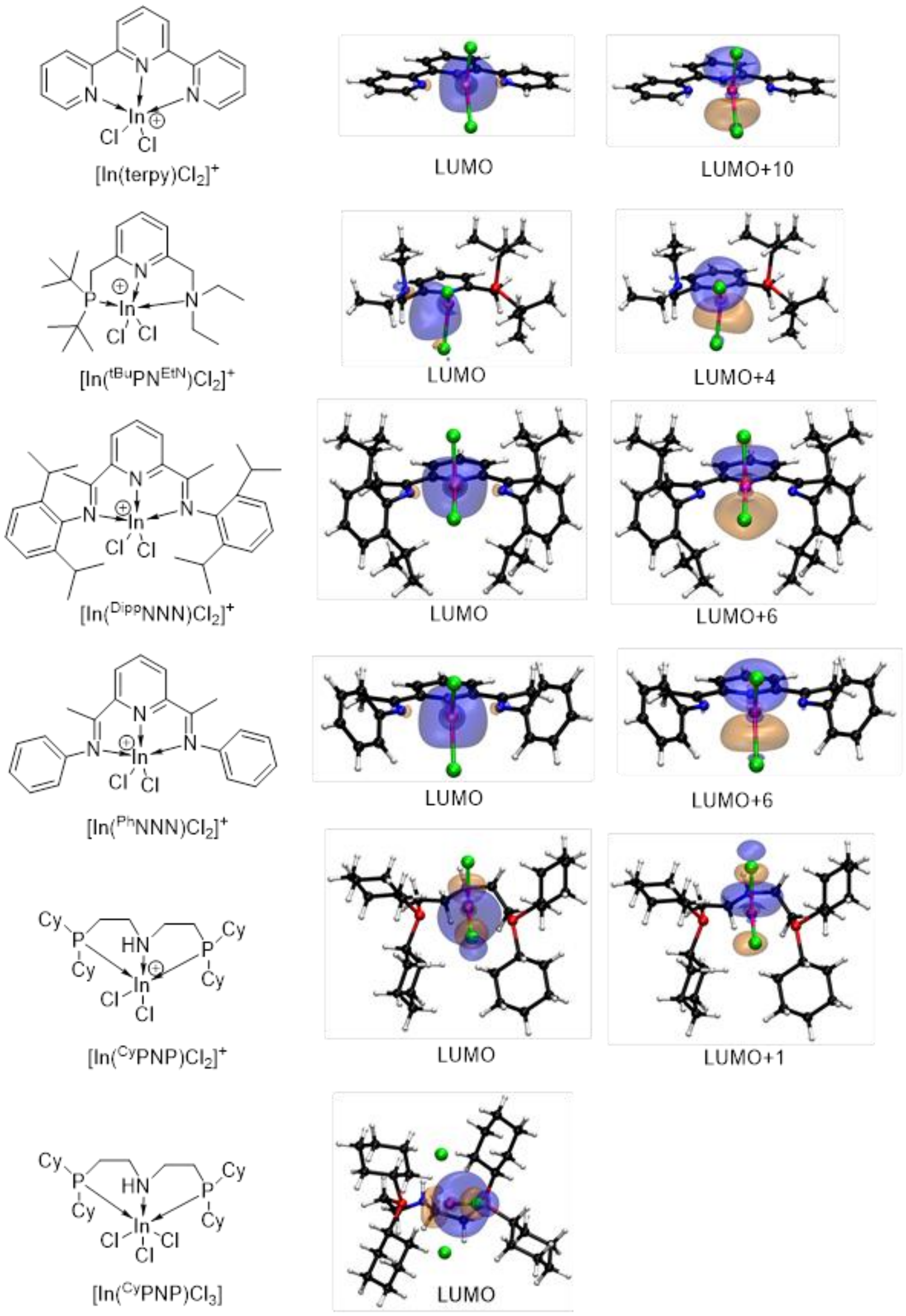

Figure S42: Indium-based unoccupied Mos

11. Substrate scope

11.1 General procedure for imine synthesis

Imines used for the catalyst testing and substrate scope were synthesized according to literature procedures.[14-15] Substituted benzaldehyde was added to a Schlenk flask followed by dropwise addition of two equivalents of the respective amine under N_2 atmosphere. The reaction was stirred overnight at room temperature in the presence of molecular sieves. Next day the excess amine was evaporated under reduced pressure. The produced imine was then kept under vacuum overnight to obtain analytically pure imine.

11.2 General procedure for amine extraction after hydrogenation

The amine products were extracted as their hydrochloride salts following a DCB–H_2O–HCl workup. The reaction mixture was transferred to a separating funnel and treated with 1 M HCl solution. This converted the amines into their corresponding hydrochloride salts, which were collected in the aqueous layer. Evaporation of the aqueous phase afforded the amine hydrochlorides in 15–99% yield.

N-benzyl-2-methylpropan-2-amine

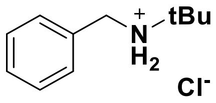

^{1}H NMR (300 MHz, cdcl$_3$) δ 7.67 – 7.58 (m, 2H), 7.33 (s, 3H), 3.86 (s, 2H), 1.34 (s, 9H).
ESI-MS(M+H): expected 163.3, found 162.0 m/z

N-(4-methoxybenzyl)-2-methylpropan-2-amine

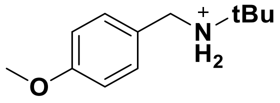

^{1}H NMR (400 MHz, DMSO) δ 7.63 – 7.48 (m, 2H), 7.04 – 6.88 (m, 2H), 4.09 – 3.91 (m, 2H), 3.78 (s, 3H), 1.38 (s, 9H); ESI-MS(M+H) =194.156 (found), 194.15 (expected)

N-(4-chlorobenzyl)-2-methylpropan-2-amine

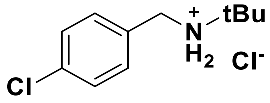

^{1}H NMR (300 MHz, CDCl$_3$) δ 7.66 – 7.56 (m, 2H), 7.32 (dt, J = 8.7, 2.3 Hz, 2H), 3.91 (s, 2H), 1.34 (s, 9H); ESI-MS(M+H) =198.106, expected-198.10

1-cyclohexyl-N-methylethan-1-amine

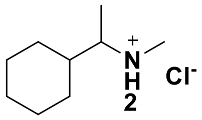

^{1}H NMR (400 MHz, DMSO) δ 3.01 – 2.92 (m, 1H), 1.85 – 1.50 (m, 9H), 1.26 – 1.15 (m, 2H), 1.11 (d, J = 6.8 Hz, 4H), 1.07 – 0.97 (m, 3H); ESI-MS(M+H)-142.16, Expected 142.15

N-(2,4-dimethoxybenzyl)-2-methylpropan-2-amine

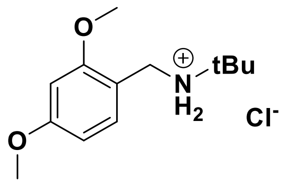

^{1}H NMR (400 MHz, DMSO) δ 9.15 (d, *J* = 6.6 Hz, 2H), 7.46 (d, *J* = 2.0 Hz, 1H), 7.10 (dd, *J* = 8.2, 1.9 Hz, 1H), 6.96 (d, *J* = 8.2 Hz, 1H), 4.03 – 3.97 (m, 2H), 3.77 (d, *J* = 14.6 Hz, 6H), 1.38 (s, 9H); ESI-MS(M+H) = found 224.16, expected 224.16

methyl 4-((tert-butylamino)methyl)benzoate

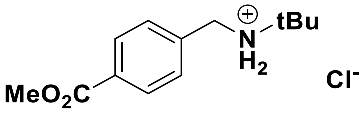

^{1}H NMR (400 MHz, DMSO) δ 9.46 (d, *J* = 9.2 Hz, 2H), 7.95 (d, *J* = 8.0 Hz, 2H), 7.81 (d, *J* = 8.0 Hz, 2H), 4.22 – 4.08 (m, 2H), 3.85 (s, 3H), 1.32 (d, *J* = 55.3 Hz, 9H); ESI-MS(M+H)= found 222.151, expected 222.15

2-methyl-N-(4-methylbenzyl)propan-2-amine

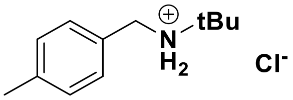

^{1}H NMR (400 MHz, DMSO) δ 7.61 – 7.46 (m, 2H), 7.24 (d, *J* = 7.8 Hz, 2H), 4.11 – 3.95 (m, 2H), 2.33 (s, 3H), 1.38 (s, 9H); ESI-MS(M+H)=Found 178.160, expected 178.15

N-benzylpropan-2-amine

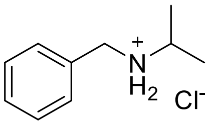

^{1}H NMR (400 MHz, $CDCl_3$) δ 7.72 – 7.64 (m, 2H), 7.47 – 7.35 (m, 3H), 4.10 (s, 2H), 3.25 (hept, *J* = 6.4 Hz, 1H), 1.51 – 1.47 (m, 6H); ESI-MS(M+H)=Found 149.932, expected 150.12

N-(tert-butyl)-2,2-dimethylpropan-1-amine

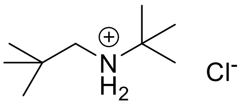

^{1}H NMR (300 MHz, $cdcl_3$) δ 2.76 (s, 2H), 1.56 (s, 9H), 1.19 (d, *J* = 1.7 Hz, 9H); ESI-MS (M+H) = Found 145.443, expected 144.17

N-(cyclohex-3-en-1-ylmethyl)-2-methylpropan-2-amine

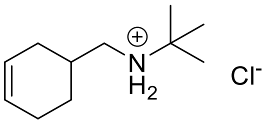

^{1}H NMR (400 MHz, $CDCl_3$) δ 8.66 (s, 2H), 5.66 (dddt, *J* = 14.3, 10.0, 6.2, 2.1 Hz, 2H), 2.86 (td, *J* = 11.9, 6.5 Hz, 2H), 2.55 – 2.46 (m, 1H), 2.33 (td, *J* = 8.5, 4.5 Hz, 1H), 2.18 – 2.08 (m, 2H), 1.92 – 1.81 (m, 1H), 1.55 (d, *J* = 2.1 Hz, 9H); ESI-MS(M+H) = Found 168.37, expected 168.17

N-(cyclohexylmethyl)-2-methylpropan-2-amine

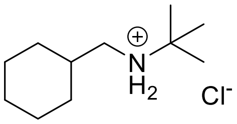

^{1}H NMR (400 MHz, $CDCl_3$) δ 8.58 (s, 1H), 2.81 – 2.70 (m, 2H), 2.20 – 2.10 (m, 2H), 1.99 (dddd, *J* = 14.4, 11.1, 5.6, 3.3 Hz, 1H), 1.77 – 1.64 (m, 3H), 1.54 (d, *J* = 1.3 Hz, 9H), 1.35 – 1.12 (m, 3H), 1.05 – 0.93 (m, 2H); ESI-MS(M+H)=Found 170.20 expected 170.18

N-methyl-1-phenylmethanamine

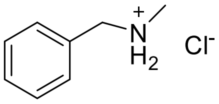

^{1}H NMR (400 MHz, $CDCl_3$) δ 7.66 – 7.58 (m, 2H), 7.49 – 7.40 (m, 3H), 4.13 (s, 2H), 2.56 (s, 3H); ESI-MS(M+H) = Found 122.37, expected 122.09

N-(2,4-dimethylbenzyl)-2-methylpropan-2-amine

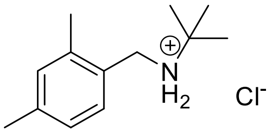

^{1}H NMR (400 MHz, $CDCl_3$) δ 7.59 (d, *J* = 7.6 Hz, 1H), 7.04 – 6.95 (m, 2H), 3.96 – 3.80 (m, 2H), 2.48 (s, 3H), 2.24 (s, 3H), 1.33 (s, 9H);

2-methyl-N-(pyridin-3-ylmethyl)propan-2-amine

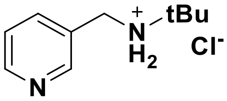

^{1}H NMR (400 MHz, $CDCl_3$) δ 8.61 – 8.48 (m, 3H), 7.75 (dt, *J* = 7.8, 2.0 Hz, 1H), 3.78 (s, 2H), 1.22 (s, 9H); ESI-MS(M+H) = Found 165.35 expected 165.13

Diphenylmethanamine

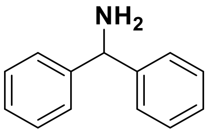

The corresponding imine was purchased from abcr (CAS 1013-88-3); only 5% in crude NMR sample, which was confirmed by mass spectrometry. Isolation was not successful and NMR could not be obtained.

ESI-MS(M+H)=Found 184, expected 184.1

12. References

(1) Saha, P.; Zábranský, M.; English, L. E.; Jupp, A. R.; Hulla, M. Cationic Indium Complexes Halve H_2 Partial Pressure in CO2 Reductive Coupling Reactions with Amines. *J. Catal.* **2025**, *452*, 116445. https://doi.org/10.1016/j.jcat.2025.116445

(2) Singh, A. P.; Roesky, H. W.; Carl, E.; Stalke, D.; Demers, J. P.; Lange, A. Lewis Base Mediated Autoionization of $GeCl_2$ and $SnCl_2$. *J. Am. Chem. Soc.* **2012**, *134,* 10, 4998–5003. https://doi.org/10.1021/ja300563g.

(3) Sheldrick, G. M. SHELXT – Integrated space-group and crystal-structure determination *Acta Cryst. A*, **2015**, 71, 3-8. https://doi.org/10.1107/S2053273314026370

(4) Sheldrick, G. M. Crystal structure refinement with SHELXL. *Acta Cryst. C*, **2015**, *71* (1), 3-8. https://doi.org/10.1107/S2053229614024218

(5) Spek, A. L. PLATON SQUEEZE: a tool for the calculation of the disordered solvent contribution to the calculated structure factors. *Acta Cryst. C*, **2015**, *71* (1), 9-18. https://doi.org/10.1107/S2053229614024929

(6) P. Erdmann, L. Greb, *ChemPhysChem*. **2021**, 22, 935–943. https://doi.org/10.1002/cphc.202100150

(7) P. Erdmann, J. Leitner, J. Schwarz, L. Greb, *ChemPhysChem*, **2020**, 21, 987 –994. https://doi.org/10.1002/cphc.202000244

(8) Neese, F. *The ORCA program system Wiley Interdiscip. Rev.: Comput. Mol. Sci.*, **2012**, *2*, *1*, 73–78. Update: Neese, F. *Software update: the ORCA program system – Version 6.0 Wiley Interdiscip. Rev.: Comput. Mol. Sci.*, **2025**, *15*, *2*, e70019

(9) D. J. Frisch, M. J.; Trucks, G. W.; Schlegel, H. B.; Scuseria, G. E.; Robb, M. A.; Cheeseman, J. R.; Scalmani, G.; Barone, V.; Petersson, G. A.; Nakatsuji, H.; Li, X.; Caricato, M.; Marenich, A. V.; Bloino, J.; Janesko, B. G.; Gomperts, R.; Mennucci, B.; Hratch, **2016**, Gaussian, Inc., Wallingford CT

(10) Y. Zhao, D. G. Truhlar, *Theor. Chem. Acc.* **2008**, *120*, 215–241. https://doi.org/10.1007/s00214-007-0310-x

(11) M. Cossi, V. Barone, R. Cammi, J. Tomasi, *Chem. Phys. Lett.* **1996**, *255*, 327–335. https://doi.org/10.1016/0009-2614(96)00349-1

(12) Humphrey, W., Dalke, A. and Schulten, K., "VMD - Visual Molecular Dynamics", J. Molec. Graphics, 1996, 14, 33-38. https://doi.org/10.1016/0263-7855(96)00018-5

(13) CYLview20; Legault, C. Y., Université de Sherbrooke, **2020** (http://www.cylview.org)

(14) J. Mlochowski, E. Kubicz, K. Kloc, M. Mordarski, W. Peczyńska and L. Syper, Liebigs *Ann Chem*, **1988**, 455-464. https://doi.org/10.1002/jlac.198819880515

(15) H. Nagae, Y. Shibata, H. Tsurugi, K. Mashima Aminomethylation Reaction of ortho-Pyridyl C-H Bonds Catalyzed by Group 3 Metal Triamido Complexes. *J. Am. Chem. Soc.* **2015**, *137*, 2, 640–643. https://doi.org/10.1021/ja511964k
